# Monovalent pseudo-natural products supercharge degradation of IDO1 by its native E3 KLHDC3

**DOI:** 10.1038/s41557-025-02021-5

**Published:** 2026-01-07

**Authors:** Elisabeth Hennes, Belén Lucas, Natalie S. Scholes, Xiu-Fen Cheng, Daniel C. Scott, Matthias Bischoff, Katharina Reich, Raphael Gasper, María Lucas, Teng Teng Xu, Sofia Rossini, Lisa-Marie Pulvermacher, Lara Dötsch, Hana Imrichova, Alexandra Brause, Siska Führer, Kesava Reddy Naredla, Sonja Sievers, Kamal Kumar, Petra Janning, Ciriana Orabona, Malte Gersch, Peter J. Murray, Brenda A. Schulman, Georg E. Winter, Slava Ziegler, Herbert Waldmann

**Affiliations:** 1 Max-Planck-Institut für Molekulare Physiologie, Abteilung Chemische Biologie, Dortmund, Germany; 2 Technische Universität Dortmund, Fakultät Chemie und Chemische Biologie, Dortmund, Germany; 3 CeMM Research Center for Molecular Medicine of the Austrian Academy of Sciences, Vienna, Austria; 4 Department of Structural Biology, St. Jude Children’s Research Hospital, Memphis, TN USA; 5 Compound Management and Screening Center Otto-Hahn-Str.11, Dortmund, Germany; 6 Max-Planck-Institut für Molekulare Physiologie, Zentrale Einheit für Kristallographie und Biophysik, Dortmund, Germany; 7 Instituto de Biomedicina y Biotecnología de Cantabria, Universidad de Cantabria-CSIC, Santander, Spain; 8 Immunoregulation Research Group, Max Planck Institute of Biochemistry, Martinsried, Germany; 9 Department of Medicine and Surgery, University of Perugia, Perugia, Italy; 10 Chemical Genomics Centre, Max-Planck-Institut für Molekulare Physiologie, Dortmund, Germany; 11 Department of Molecular Machines and Signaling, Max Planck Institute of Biochemistry, Martinsried, Germany; 12 AITHYRA Research Institute for Biomedical AI, Vienna, Austria

**Keywords:** Mechanism of action, Enzymes

## Abstract

Targeted protein degradation modulates protein function beyond the inhibition of enzyme activity or protein–protein interactions. Most degrader drugs function by directly mediating the proximity between a neosubstrate and a hijacked E3 ligase. Here we identify pseudo-natural products derived from (−)-myrtanol, termed iDegs, that inhibit and induce degradation of the immunomodulatory enzyme indoleamine-2,3-dioxygenase 1 (IDO1) by a distinct mechanism. iDegs boost IDO1 ubiquitination and degradation by the cullin-RING E3 ligase CRL2^KLHDC3^, which we identified to natively mediate ubiquitin-mediated degradation of IDO1. Therefore, iDegs increase IDO1 turnover using the native proteolytic pathway. In contrast to clinically explored IDO1 inhibitors, iDegs reduce the formation of kynurenine by both inhibition and induced degradation of the enzyme and thus also modulate the non-enzymatic functions of IDO1. This unique mechanism of action may open up alternative therapeutic opportunities for the treatment of cancer beyond classical inhibition of IDO1.

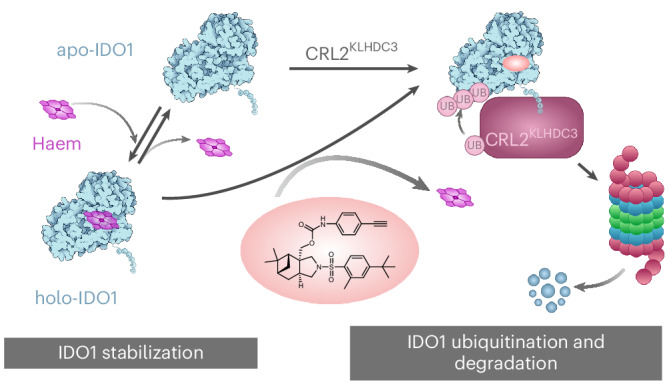

## Abstract (web summary)

In targeted protein degradation, a degrader molecule brings a neosubstrate protein proximal to a hijacked E3 ligase for its ubiquitination. Here, pseudo-natural products derived from (−)-myrtanol—iDegs—are identified to inhibit and induce degradation of the immunomodulatory enzyme indoleamine-2,3-dioxygenase 1 (IDO1) by a distinct mechanism. iDegs prime apo-IDO1 ubiquitination and subsequent degradation using its native proteolytic pathway.

## Main

Current strategies for small-molecule-mediated targeted protein degradation (TPD)^1^ rely on compound-mediated induction of proximity between a neosubstrate and an E3 ubiquitin ligase^2^. Such ‘degraders’ can either be heterobifunctional, harbouring distinct entities for the target and E3 (‘PROTACs’), or monovalent, binding to either the target or the ligase to adapt its surface and induce a cooperative tripartite assembly (molecular glue degraders, MGDs). Both degrader types have entered clinics^3^, and the discovery of alternative chemotypes that may unlock previously unexplored TPD strategies is highly desirable.

Natural products (NPs) and their analogues have yielded diverse inducers of protein degradation^4^, raising the possibility that new degrader chemotypes could be derived from NPs. Pseudo-natural products combine natural-product fragments in arrangements and combinations not observed in NPs. They retain the biological relevance of NPs but open new chemical space and therefore may have unexpected and novel targets^5,6^, so exploration of their bioactivity^5,7^ may identify novel small-molecule degrader chemotypes and E3 ligases.

The haem-binding enzyme indoleamine-2,3-dioxygenase 1 (IDO1) converts tryptophan (Trp) to kynurenine (Kyn), and Trp shortage and Kyn elevation are linked to reduced anti-tumour immunity, though by different mechanisms^8–12^. Moreover, IDO1 expression and Kyn levels are related to Epstein Barr virus (EBV)-associated lymphoma^13^ and to neurodegeneration^14,15^. In general, clinical exploration of different IDO1 inhibitors for cancer treatment has^16–22^, despite encouraging preclinical data^23,24^, met with limited success^25,26^. Plausible reasons include that IDO1 may have non-enzymatic functions^27–34^. Nevertheless, IDO1 inhibitors are in active clinical trials^35^. The limitations of enzymatic inhibitors^36^ may be overcome by IDO1 degradation, as initially supported by IDO1-directed proteolysis-targeting chimeras (PROTACs)^37–39^, but monovalent IDO1 degraders have yet to be identified.

We have identified a class of pseudo-natural products derived from (−)-myrtanol, termed iDegs, that both inhibit IDO1 and induce IDO1 degradation. iDegs induce structural changes in IDO1 that cause enhanced ubiquitination and augmented degradation by CRL2^KLHDC3^, a ligase we identified to also mediate the ubiquitination and native degradation of IDO1. Our work defines a unique mechanism of action, a previously not identified type of degrader, and reveals an E3-ligase previously not used for small-molecule-mediated protein degradation.

## Results

### Identification of IDO1 degraders

**Fig. 1:**
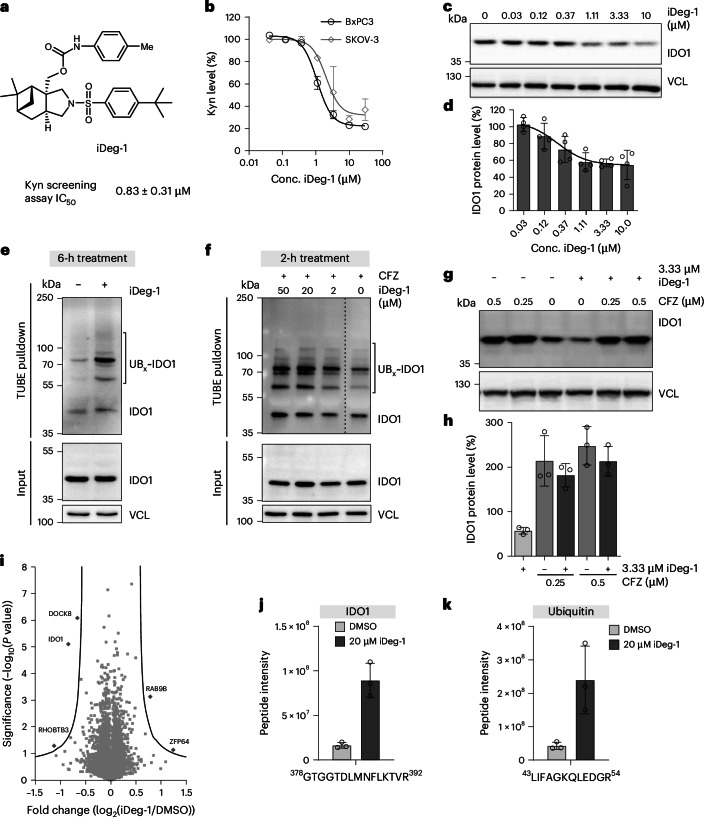
iDeg-1 reduces IDO1 protein levels via the UPS. **a**, Structure of screening hit iDeg-1 and IC_50_ value in the Kyn assay in BxPC3 cells. Data are presented as mean value ± s.d.; *n* = 3 biological replicates. **b**, Kyn assay in BxPC3 and SKOV-3 cells after treatment with iDeg-1 and 50 or 5 ng ml^−1^ IFN-γ, respectively, for 48 h before detection of Kyn levels utilizing *p*-DMAB. **c**,**d**, IDO1 protein levels in BxPC3 cells upon treatment with IFN-γ and iDeg-1 for 24 h. Representative immunoblots (**c**) and quantified band intensities from **c** (**d**). Data are presented as mean values ± s.d.; *n* = 4 biological replicates. VCL, vinculin. **e**,**f**, Detection of ubiquitinated IDO1 by means of a TUBE pulldown after treatment of IFN-γ-stimulated BxPC3 cells with iDeg-1 or DMSO. **e**, Cells were treated for 6 h with 50 µM iDeg-1 before the TUBE pulldown. Representative immunoblots of *n* = 3 biological replicates are shown. **f**, Cells were treated with 450 nM carfilzomib (CFZ) 60 min before the addition of iDeg-1 or DMSO for 2 h, followed by TUBE pulldown. Representative immunoblots of *n* = 3 biological replicates for IDO1 are shown. UB, ubiquitin. The dashed line indicates where lanes of the same blot were spliced together. **g**,**h**, HEK239T cells were electroporated with rhIDO1 protein. Cells were treated with CFZ for 30 min before the addition of 3.33 µM iDeg-1 and further incubation for 6 h. **g**, Representative immunoblot of *n* = 3 biological replicates. **h**, Quantified band intensities from **g**, representing samples treated with compound relative to DMSO (set to 100%). Data are presented as mean values ± s.d.; *n* = 3 biological replicates. **i**, Volcano plot of iDeg-1-induced changes in the global proteome. HEK239T cells were electroporated with rhIDO1 protein followed by treatment with 10 µM iDeg-1 or DMSO for 6 h and MS analysis. In total, 7,541 proteins were detected. Plot generated using Perseus. Statistical significance was assessed using both-sided *t*-test with error-corrected *P* values (S0 = 0.5 (non-linear cutoff), FDR = 0.01). **j**,**k**, IDO1 immunoprecipitation (IP) and identified peptide of IDO1 (**j**) or ubiquitin (**k**) with diGly modification. IFN-γ-stimulated BxPC3 cells were treated with 20 µM iDeg-1 or DMSO for 6 h before the IP. Data are presented as mean values ± s.d.; *n* = 3 biological replicates. Source data

**Extended Data Fig. 1:**
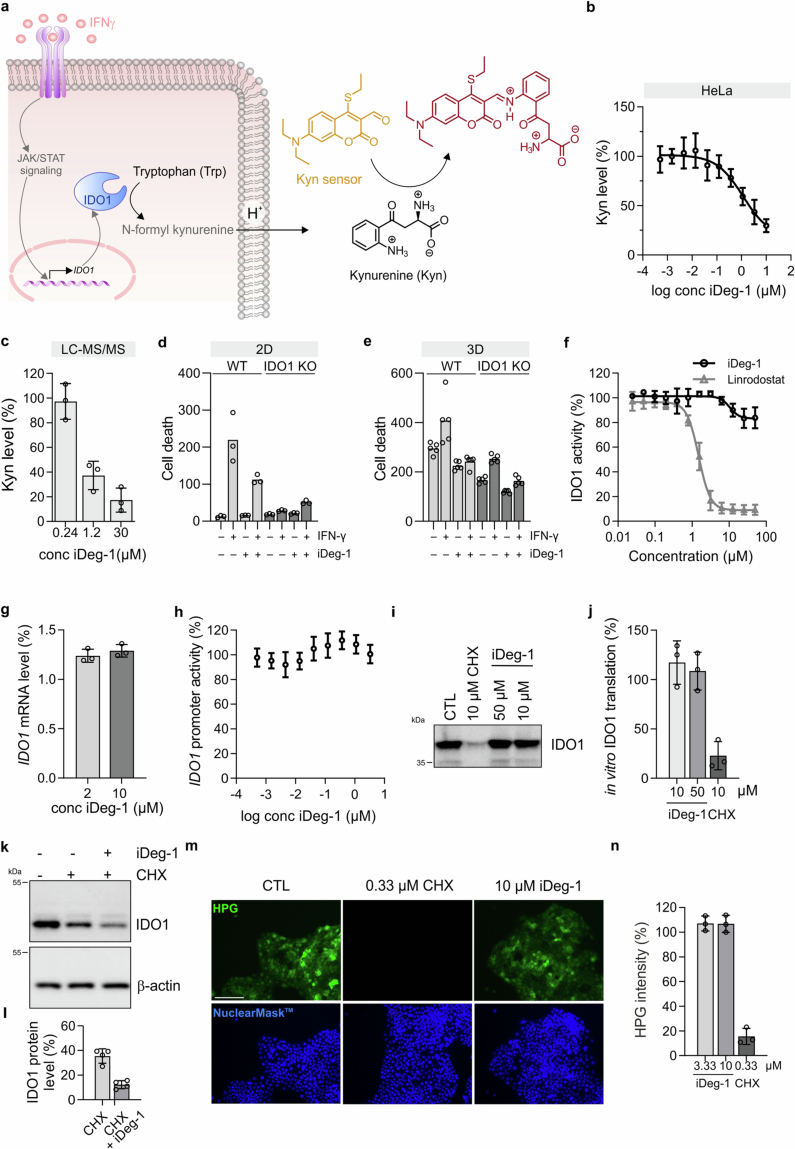
Validation of iDeg-1 as a small molecule reducing IDO1 protein levels. **a**, Schematic overview of the Kyn screening assay. BxPC3 cells were stimulated with IFN-γ to induce IDO1 expression via the JAK/STAT signaling pathway. After 48 h treatment with IFN-γ, Trp and the compounds, cells were hydrolysed and Kyn levels are quantified in the cell supernatant by means of the Kyn sensor. Fluorescence intensity (ex/em 535/595 nm) is proportional to the Kyn amount generated by the cells. **b**, Kyn assay in HeLa cells treated with IFN-γ, Trp and iDeg-1 for 48 h prior Kyn level detection utilizing *p*-DMAB. Mean values ± SD, n = 3 biological replicates. **c**, Kyn levels in BxPC3 cells treated with IFN-γ, Trp and iDeg-1 for 48 h. Kyn levels were detected using LC–MS/MS. Mean values ± SD, n = 3 biological replicates. **d,e**, Influence of iDeg-1 (5 µM) on cell death in SKOV-3 cells WT or IDO1 knockout (KO) cells grown in 2D (**d**) or 3D (**e**). Cell death was detected by means of CellTox Green in presence or absence of IFN-γ (1 ng/mL). Representative data of n = 3 biological replicates with N = 3 technical replicates for 96 h (**d**) or N = 5 technical replicates for 6 days (**e**). **f**, Influence of iDeg-1 on the *in vitro* IDO1 enzymatic activity. rhIDO1 was pre-incubated with the compounds at 37 °C for 90 min prior to detection of Kyn levels using *p*-DMAB. The apo-IDO1 inhibitor linrodostat was used as a control. Mean values ± SD, n = 3 biological replicates. **g**, *IDO1* mRNA expression levels. BxPC3 cells were treated with IFN-γ and iDeg-1 for 24 h prior to isolation of RNA and RT-qPCR analysis. Mean values ± SD, n = 3 biological replicates. **h**, Influence on the *IDO1* promoter activity. HEK293T cells were transfected with a construct encoding firefly luciferase (Fluc) under the control of the *IDO1* promoter and a plasmid for constitutive expression of *Renilla* luciferase (Rluc). Cells were treated with IFN-γ and the compounds for 24 h. Fluc/Rluc ratios were normalized to samples that were treated with IFN-γ only (set to 100%). Mean values ± SD, n = 3 biological replicates. **i**,**j**, *In vitro* IDO1 protein translation. IDO1 translation was analysed in cell lysates in the presence of iDeg-1, cycloheximide (CHX) or DMSO (CTL). Representative immunoblot (one of n = 3 biological replicates) (**i**) and quantified band intensities (**j**). **j**, Mean values ± SD, n = 3 biological replicates. **k, l**, CHX chase assay. BxPC3 cells were treated with IFN-γ for 24 h prior to addition of 5 µM CHX or 5 µM CHX and 10 µM iDeg-1. Representative immunoblot (one of n = 4 biological replicates) (**k**). (**l**) Normalized band intensities from **k**. Mean values ± SD, n = 4 biological replicates. **m, n**, Analysis of global protein translation in cells 24 h after compound treatment by means of HPG detection. Representative images of n = 3 biological replicates of HPG intensities displaying protein translation (green) and nuclear stain (blue). Scale bar: 100 µm. **n**, Normalized HPG signal intensities of the images in **m**. Mean values ± SD, n = 3 biological replicates. Source data

A library of 157,332 small molecules were screened in a cell-based assay (Kyn assay; Extended Data Fig. 1a) that quantified Kyn, the product of the IDO1 reaction. So as to screen under conditions that would be sensitive to IDO1 levels, we considered that IDO1 expression can be induced by interferon gamma (IFN-γ)^40^. We thus performed the screen in IFN-γ-stimulated BxPC3 cells^21^. The (−)-myrtanol-derived pseudo-natural product, hereafter termed iDeg-1 (see Supplementary Scheme 1 for the synthesis), inhibited Kyn formation with a half-maximal inhibitory concentration (IC_50_) of 0.83 ± 0.31 µM in the screening assay (Fig. 1a), which we confirmed using orthogonal Kyn assays in IFN-γ-stimulated BxPC3, SKOV-3 and HeLa cells (IC_50_ values of 1.1 ± 0.1 µM, 1.6 ± 0.3 µM and 1.7 ± 1.2 µM, respectively; Fig. 1b and Extended Data Fig. 1b,c). Further indication that iDeg-1 targets IDO1 came from a cell viability assay in two-dimensional (2D) and 3D cultures^41,42^: iDeg-1 reduced IDO1-dependent SKOV-3 cell death induced by IFN-γ (Extended Data Fig. 1d,e). iDeg-1 only slightly affected IDO1 enzymatic activity (Extended Data Fig. 1f), did not impair *IDO1* transcription (Extended Data Fig. 1g,h), but dose-dependently reduced IDO1 protein levels up to 45 ± 15% at 10 µM after 24 h (Fig. 1c,d) without inhibiting in vitro translation of IDO1 or global protein translation (Extended Data Fig. 1i–n).

**Extended Data Fig. 2:**
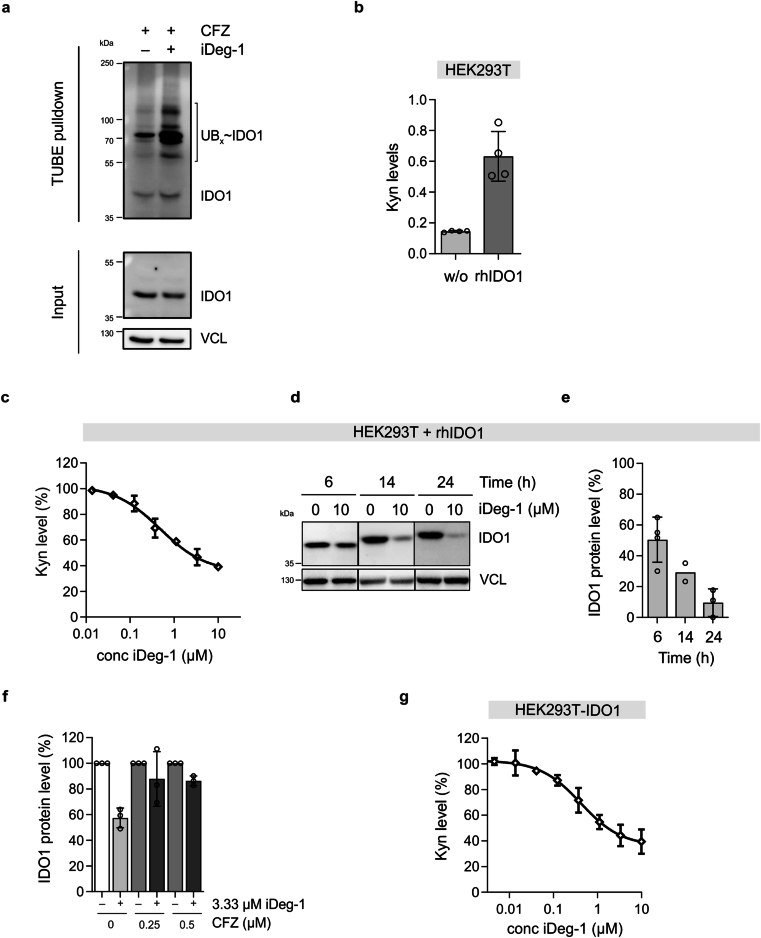
iDeg-1 inhibits Kyn production in cells by IDO1 degradation. **a**, TUBE pulldown after treatment of IFN-γ-stimulated BxPC3 cells with iDeg-1 or DMSO. Cells were pre-treated for 1 h with the proteasome inhibitor carfilzomib (CFZ, 450 nM) followed by addition of 50 µM iDeg-1 for 4 h prior to lysate preparation and TUBE pulldown. Representative immunoblot (one of n = 3 biological replicates). UB: ubiquitin. **b**, HEK293T cells were electroporated with and without IDO1 and treated with Trp. 24 h later, Kyn levels were detected using *p*-DMAB. Mean values ± SD, n = 4 biological replicates. **c**, HEK^rhIDO1^ cells treated with Trp and iDeg-1 for 24 h prior Kyn level detection using *p*-DMAB. Mean values ± SD, n = 3 biological replicates. **d,e**, HEK239T cells were electroporated with rhIDO1 protein followed by treatment with iDeg-1 (10 µM) for 6, 14 or 24 h. IDO1 protein levels were determined via immunoblotting (**d**). **e**, Normalized band intensities from d. Mean values ± SD, n = 2 (14 h), n = 3 (24 h), n = 4 (6 h) biological replicates. **f**, Normalized data from quantified band intensities from Fig. 1g. The effect of iDeg-1 in co-treatment conditions is represented relative to the treatment of carfilzomib (CFZ) alone (set to 100 %). Mean values ± SD, n = 3 biological replicates. **g**, HEK293T cells were transfected with an IDO1 expression plasmid 24 h prior the addition of Trp and iDeg-1. Kyn levels were determined 24 h after compound addition using *p*-DMAB. Mean values ± SD, n = 3 biological replicates. VCL: vinculin. Source data

To determine whether iDeg-1 induces degradation via the ubiquitin proteasome system (UPS), IFN-γ-stimulated BxPC3 cells were treated with iDeg-1 for 6 h (Fig. 1e), or for 2 and 4 h after pretreatment with the proteasome inhibitor carfilzomib (CFZ; Fig. 1f and Extended Data Fig. 2a). Increased polyubiquitination was detected with a tandem ubiquitin binding entity (TUBE) pulldown from cell lysates^43^. Ubiquitination was induced shortly after compound addition, but protein reduction was only detectable after 24 h in IFN-γ-stimulated BxPC3 cells (Fig. 1c,d). Because the capacity to induce degradation may be masked by the ongoing IFN-γ-stimulated production of IDO1, we directly introduced enzymatically active recombinant human IDO1 protein (rhIDO1) into HEK293T cells by electroporation (HEK^rhIDO1^ cells). iDeg-1 dose-dependently reduced Kyn amounts with an IC_50_ value of 0.45 ± 0.1 µM (Extended Data Fig. 2b,c) and lowered IDO1 protein after 6 h by 46% at a concentration of 10 µM (Extended Data Fig. 2d,e). CFZ inhibited degradation (Fig. 1g,h and Extended Data Fig. 2f), indicating involvement of the UPS. iDeg-1 also inhibited Kyn production in HEK293T cells, which transiently express IDO1, with an IC_50_ of 0.42 ± 0.2 µM (Extended Data Fig. 2g). Therefore, iDeg-1 depletes IDO1 independently of the effects of IFN-γ.

**Extended Data Fig. 3:**
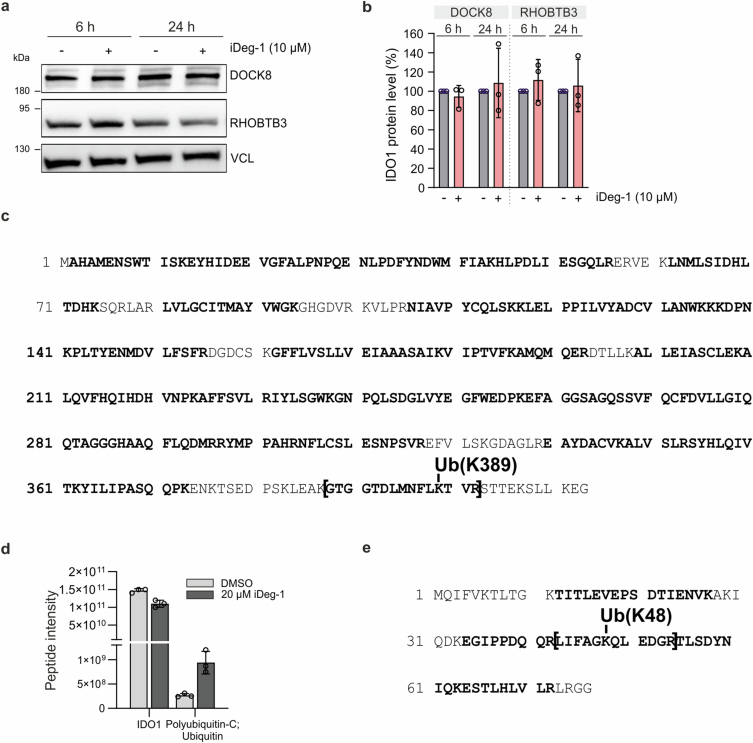
iDeg-1 causes selective IDO1 depletion by polyubiquitination of lysine 389 of IDO1. **a,b**, Influence of iDeg-1 on the protein levels of DOCK-8 and RHOBTB3 in HEK293T cells treated for 6 h or 24 h. Representative immunoblots (one of n = 3 biological replicates) (**a**) and quantification of band intensities from a (**b**). Mean values ± SD, n = 3 biological replicates. VCL: vinculin. **c-e**, IFN-γ-stimulated BxPC3 cells were treated with iDeg-1 or DMSO as a control for 6 h prior to lysate preparation. IDO1 was immunoprecipitated and the enriched fraction was digested and analysed by MS. **c**, IDO1 protein sequence. Amino acids in bold illustrate regions identified in the MS experiment. K389: the identified ubiquitination site of IDO1. Due to incomplete sequence coverage in the MS experiment, ubiquitination of additional lysines cannot be excluded. **d**, Measured relative peptide intensity for IDO1 and polyubiquitin-C. Mean values ± SD, n = 3 biological replicates. **e**, Ubiquitin protein sequence. Identified peptides are displayed in bold. The identified ubiquitination site K48 is indicated. K6, K11, K29 and K33 in ubiquitin could not be identified due to incomplete sequence coverage in the MS experiment. Source data

Global proteome profiling of HEK^rhIDO1^ cells after 6 h of treatment with iDeg-1 showed that, besides IDO1, reduced levels were detected only for DOCK-8 and RHOBTB3. However, these effects could not be validated (Fig. 1i, Extended Data Fig. 3a,b and Supplementary Table 1). Analysis of ubiquitinated proteins for a diglycine (diGly) attached to lysine residues (K-ε-diglycine) that were modified with ubiquitin^44^, after iDeg-1 treatment and IDO1 immunoprecipitation uncovered K389 of IDO1 as a site of ubiquitination (Fig. 1j and Extended Data Fig. 3c). In addition, we observed a 3.5-fold increase in ubiquitin levels in the IDO1 immunoprecipitate after treatment with iDeg-1, confirming iDeg-1-induced IDO1 ubiquitination (Extended Data Fig. 3d). DiGly analysis further revealed K48 linkages in the ubiquitin chains (Fig. 1k and Extended Data Fig. 3e), which are associated with protein degradation via the UPS^45^.

**Fig. 2:**
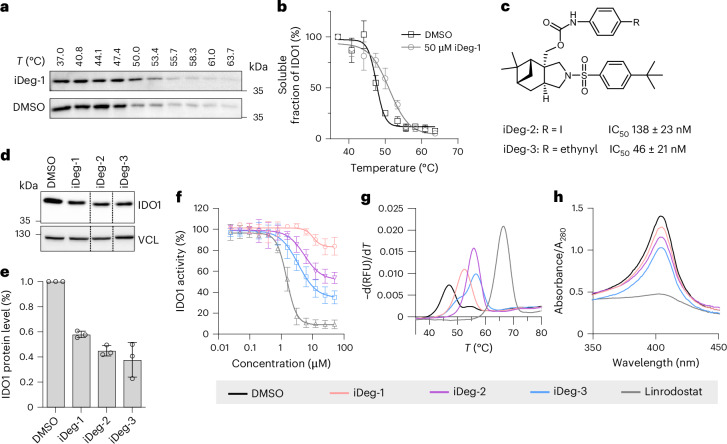
iDegs interact with IDO1. **a**,**b**, CETSA in intact SKOV-3 cells treated with 50 µM iDeg-1 or DMSO for 1 h followed by heat treatment and immunoblotting. **a**, Representative immunoblot for IDO1. **b**, Quantification of band intensities from **a**. Data are presented as mean values ± s.d.; *n* = 3. **c**, Structures of iDeg-2 and iDeg-3 and IC_50_ values in the Kyn assay in BxPC3 cells. Data are presented as mean values ± s.d.; *n* = 3 biological replicates. **d**,**e**, Influence of iDeg-1, iDeg-2 and iDeg-3 on IDO1 protein levels in BxPC3 cells. Cells were treated with IFN-γ and the compounds (3.33 µM) for 24 h before immunoblotting (**d**). Quantification of the band intensities is shown in **e**. Data are presented as mean values ± s.d.; *n* = 3 biological replicates. Dashed lines indicate where lanes of the same blot were spliced together. **f**, Influence on in vitro rhIDO1 activity. rhIDO1 was pre-incubated with the compounds at 37 °C for 90 min before the detection of Kyn levels using *p*-DMAB. Data are presented as mean values ± s.d.; *n* = 3 independent experiments. **g**, rhIDO1 thermal stability in the presence of 50 µM iDeg-1, iDeg-2 or iDeg-3 or DMSO and the apo-IDO1 inhibitor linrodostat (50 µM) using nanoDSF. rhIDO1 and the compounds were pre-incubated for 3 h at 37 °C before measurement. Representative results are shown (*n* = 3 independent experiments). **h**, Detection of haem-bound IDO1 by means of UV–vis spectroscopy in the presence of iDeg-1, iDeg-2 or iDeg-3 (100 µM), DMSO or linrodostat (100 µM). Incubation was performed at 37 °C for 3 h. Representative data are presented for *n* = 3 independent experiments. Source data

**Extended Data Fig. 4:**
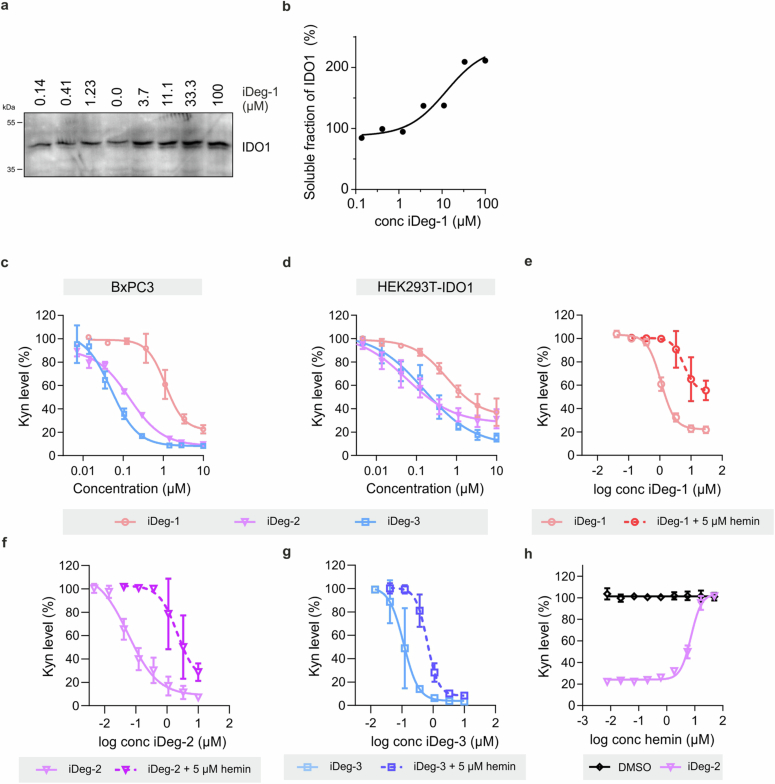
iDegs target IDO1. Validation of iDeg-2 and iDeg-3 as IDO1 degraders. **a, b**, Isothermal CETSA using iDeg-1. SKOV-3 cells were treated with iDeg-1 or DMSO as a control for 1 h at 37 °C prior to heat treatment at 50 °C, lysis and analysis of the soluble fraction via immunoblotting. Representative immunoblot (one of n = 3 biological replicates) for IDO1 (**a**) and respective melting curve (**b**). **c**, Kyn assay in BxPC3 cells treated with iDegs, Trp and IFN-γ for 48 h prior to detection of Kyn levels utilizing *p*-DMAB. Mean values ± SD, n = 3 biological replicates. **d**, HEK293T cells were transiently transfected with pCMV3-IDO1 24 h prior to compound addition. Another 24 h later, Kyn levels were detected utilizing *p*-DMAB. Mean values ± SD, n = 3 biological replicates. **e**-**g**, Influence of hemin on iDeg activity. Kyn assay in IFN-γ-stimulated BxPC3 cells for iDeg-1 (**e**), iDeg-2 (**f**) or iDeg-3 (**g**) in the presence or absence of 5 µM hemin. Kyn levels were detected after 48 h using *p*-DMAB. Mean values ± SD, n = 3 biological replicates for iDeg-1, 3 and n ≥ 2 biological replicates for iDeg-2. **h**, Dose-dependent influence of hemin on iDeg-2-mediated decrease of Kyn levels. IFN-γ-stimulated BxPC3 cells were incubated with different concentrations of hemin in presence or absence of 0.5 µM iDeg-2 for 48 h prior to detection of Kyn levels with *p*-DMAB. Mean values ± SD, n = 3 biological replicates. Source data

Direct engagement of IDO1 in cells was proven by a cellular thermal shift assay (CETSA)^46^, which showed that iDeg-1 stabilizes IDO1 with a shift in the melting temperature (Δ*T*_m_) of 3.5 ± 0.4 °C (Fig. 2a,b). An isothermal CETSA experiment at 50 °C (Extended Data Fig. 4a,b) proved that stabilization was dose-dependent.

Initial structure–activity exploration identified iDeg-2 (IC_50_ = 138 ± 23 nM) and iDeg-3 (IC_50_ = 46 ± 21 nM), which embody an iodine or an alkyne substituent, respectively, in the *para* position of the phenyl carbamate (Fig. 2c and Extended Data Fig. 4c,d). In BxPC3 cells exposed continuously to IFN-γ, iDeg-2 and iDeg-3 reduced IDO1 protein levels by 55 ± 3% and 62 ± 11%, respectively, as compared to 42 ± 2% by iDeg-1 at 3.33 µM (Fig. 2d,e). iDeg-2 and iDeg-3 also partially inhibited the enzyme in vitro (Fig. 2f) and stabilized rhIDO1, as determined by nano differential scanning fluorimetry (nanoDSF; Fig. 2g).

IDO1 binds haem as a cofactor in the active site, and exists in haem-free (apo) and haem-bound (holo) forms in cells^19^. UV–vis spectroscopic analysis revealed that in the presence of iDeg-1, iDeg-2 or iDeg-3, the specific Soret absorbance peak of haem-bound IDO1 (holo-IDO1; Fig. 2h) is reduced, showing that iDegs displace haem and, with different potencies, bind to apo-IDO1. Accordingly, addition of hemin reduced the potency of iDeg-1, iDeg-2 and iDeg-3 in the Kyn assay (Extended Data Fig. 4e–g) and dose-dependently elevated Kyn levels in the presence of iDeg-2 (Extended Data Fig. 4h). As we detected both enzymatic inhibition and degradation of IDO1 by iDeg-3, the compound class was termed iDeg.

### Crystal structure shows iDegs alter conformation of the IDO1 C-terminal region

**Fig. 3:**
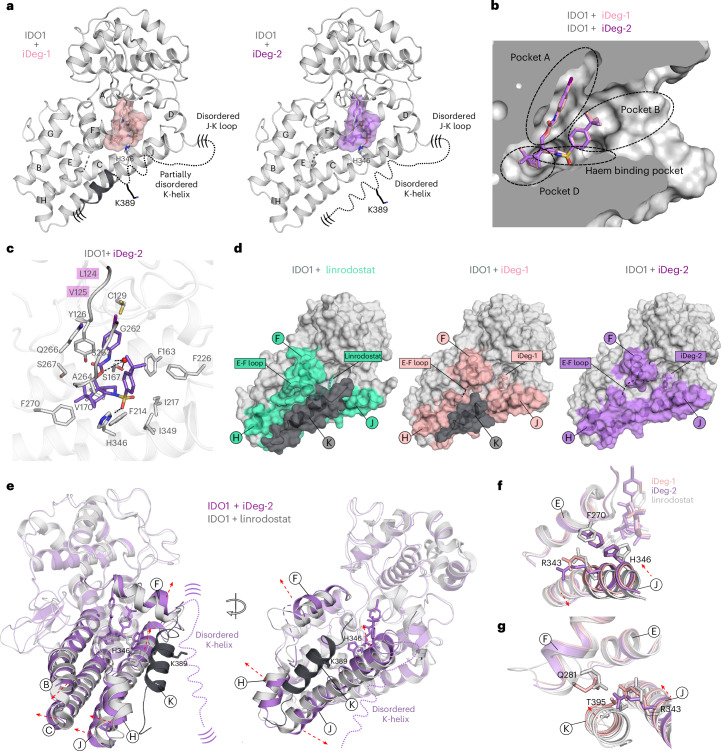
Co-crystal structures reveal that iDeg-1 and iDeg-2 bind to apo-IDO1 and induce conformational changes in its C-terminal region. **a**, Cartoon diagram of the IDO1-iDeg-1 and IDO1-iDeg-2 structures. Dashed lines indicate regions lacking electron density, including the E–F loop, J–K loop and K-helix, with parentheses denoting their proposed flexibility. The positions of ubiquitinated K389 within the K-helix and H346 are highlighted. The α-helices of the large domain (residues 121–403) are labelled A to K. **b**, Surface model (cut-in side view) showing iDeg-1 and iDeg-2 in the active site of the IDO1–iDeg-2 structure. Hydrophobic pockets A, B and D and the haem binding site are indicated. **c**, Zoom-in view of the amino acids involved in iDeg-2 binding. Hydrogen bonds are indicated by black dashed lines. **d**, Comparison of the IDO1 structures with linrodostat (PDB 6DPR-B), iDeg-1 and iDeg-2, illustrating the transition of the K-helix from a rigid to a dynamic conformation upon iDeg binding. Surface regions interacting with the K-helix are highlighted. **e**, Overlay of IDO1-iDeg-2 (violet) and IDO1–linrodostat (grey; PDB 6DPR-B) structures. iDeg binding triggers local and long-distance structural perturbations (red dashed arrows). **f**, iDeg-2 binding induces a conformational shift in H346, moving the J-helix towards the iDeg-2 binding pocket, including R343. F270 also adopts a shifted conformation. **g**, Residues T395, Q281 and R343, which are involved in stabilizing the K-helix through hydrogen bonding, similarly adopt shifted positions in the iDeg-bound state.

**Extended Data Fig. 5:**
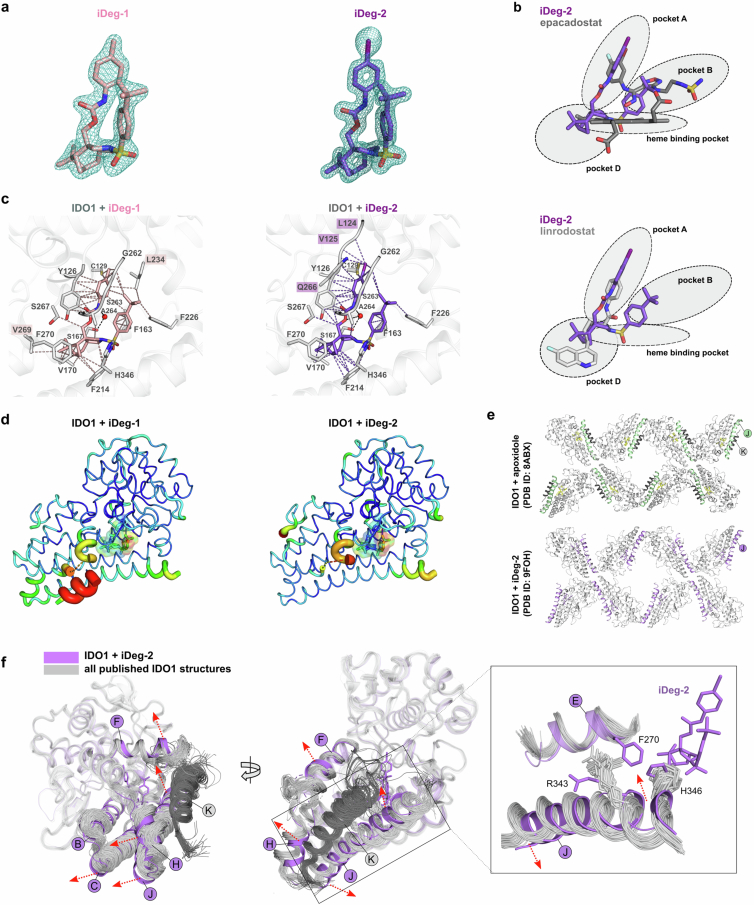
Co-crystal structures of IDO1 with iDeg-1 and iDeg-2. **a**, Electron density maps (2Fo-Fc) calculated with phases from the final refined models, contoured at 1.5 σ, showing the refined iDeg-1 and iDeg-2 structure in stick representation. **b**, Overlay of iDeg-2 (violet) with the holo-IDO1 inhibitor epacadostat in complex with haem (PDB ID: 5WN8, dark gray) and with the apo-IDO1 inhibitor linrodostat (PDB ID: 6DPRB, light gray). **c**, Cartoon representation of the iDeg-1 and iDeg-2 binding sites. Residues involved in hydrophobic interactions and H-bonds are indicated. Hydrophobic interactions between iDegs and IDO1-protein within a range of 4.0 Å are shown with dashed lines in the color corresponding to each iDeg. Hydrogen bonds are indicated as black dashed lines. **d**. Putty diagrams representation of the relative B-factor changes generated in PyMOL. Blue colors with small diameter tubes indicate lower B-factors and less mobility, while orange to red colors with large diameter tubes indicate regions of higher B-factors and increased mobility within the proteins. **e**, The highly similar crystal lattice packing of IDO1-iDeg-2 and IDO1-apoxidole indicates that the absence of electron density for the K-helix (dark gray) in the iDeg-2 structure is not an artefact of crystal packing. The J-helix is highlighted in green (8ABX) or purple (9FOH). Apoxidole and iDeg-2 are shown in yellow and purple, respectively. **f**, Overlay of the IDO1-iDeg-2 structure with all published IDO1 structures, excluding seven with high coordinate uncertainty. Shown are the front view, a 90° rotated side view, and a zoom-in view of the E- and J-helices, with the positions of H346 and R343 indicated. PDB IDs included: 7B1O, 7AH5, 7AH6, 7AH4, 6KOF, 8I7L, 6O3I, 8FUR, 7A62, 6MQ6A, 6MQ6B, 7P0N, 7P0R, 7M7D, 7NGE, 6F0A, 6R63, 7E0T, 5WN8, 5WHR, 6PU7, 5WMV, 5WMU, 5WMX, 5WMW, 6KPS, 6X5Y, 6WJY, 6UBP, 7E0P, 6PZ1, 7E0U, 7E0S, 6V52, 6WPE, 6KW7, 2D0T, 6E41, 7E0Q, 5EK3, 6DPQA, 6DPQB, 6E43, 6E46, 4U74, 6CXV, 5EK2, 5EK4, 5ETW, 6E45, 6E44, 6CXU, 6E40, 6E35, 6DPRA, 6DPRB, 4PK5, 4U72, 6AZU, 6E42, 8ABX, 7ZV3, 7RRD, 7Z2L, 7RRB, 7RRC, 7YXT and 8U5I.

The co-crystal structure of IDO1 in complex with iDeg-1 (PDB 9RIS) and iDeg-2 (PDB 9FOH) at 2.1 Å and 1.6 Å resolution, respectively (Supplementary Table 2 and Extended Data Fig. 5a), revealed that iDegs reside in the haem binding site in IDO1. The phenyl carbamate occupies lipophilic pocket A^47^ in the distal haem site, and the pyrrolidine and the sulfonyl group occupy the haem binding pocket. The monoterpene scaffold only slightly protrudes into the D-pocket, and the *tert*-butyl phenyl group is located in the solvent-exposed B-pocket (Fig. 3a,b). Interestingly, binding to this latter pocket has previously been observed mainly for holo-IDO1 inhibitors^47^ (Extended Data Fig. 5b). iDegs binding occurs through numerous hydrophobic interactions, a water-bridged hydrogen bond between the carbamate nitrogen and the hydroxyl group of S167 and a hydrogen bond between the sulfonyl oxygen of iDeg-2 and H346 (Fig. 3c). As many of the same IDO1 residues are responsible for haem coordination, this binding mode is mutually exclusive with haem and ensures that iDegs can only bind apo-IDO1. The iodine moiety of iDeg-2 establishes additional hydrophobic contacts within the distal region of the A-pocket, specifically with the main chain of L124 and V125 (Extended Data Fig. 5c). These interactions probably contribute to the enhanced activity of iDeg-2 compared to iDeg-1 (Fig. 2). Comparisons to all IDO1 structures revealed that iDeg-1 and iDeg-2 induce a striking conformational rearrangement (Fig. 3d,e). In the presence of iDeg-1, the electron density map allowed the building of a shorter C-terminal K-helix although with comparatively high B-factors. In contrast, in the iDeg-2-bound structure, no electron density for the K-helix was detected (Fig. 3d and Extended Data Fig. 5d). This absence is not attributed to crystal packing forces, as the K-helix is present in the crystal structure of IDO1 with the inhibitor apoxidole with identical space group, unit cell and similar crystallization conditions (PDB 8ABX; Extended Data Fig. 5e)^48^. An overlay of the structures of IDO1 bound to iDeg-2 or with the apo-IDO1 inhibitor linrodostat revealed that helices B, C, F, H and J are all reoriented and the J-helix is also remodelled in the iDeg complex (Fig. 3e). In all previously reported IDO1 structures, the K-helix is embraced through F, J and H helices and the E–F loop (Extended Data Fig. 5f). In contrast, in the iDeg-1- and iDeg-2-bound complexes, tethers to the K-helix are weakened due to cumulative movements of several amino acids within the J-helix. Specifically, a substantial rotation and translation of H346, which coordinates the central iron of the haem group in holo-IDO1, shifts the J-helix C terminus towards the iDeg-2 binding pocket. Residues R343, F270 and T395 also adopt an alternative conformation compared to all other published IDO1 structures (Fig. 3f,g and Extended Data Fig. 5f). Weakened interactions between the K-helix and surrounding helices F, J and H and the E–F loop due to their reorientation presumably increase the flexibility of the K-helix, accounting for its invisibility in the electron density map. As previously reported IDO1 inhibitors do not substantially alter the overall structure of IDO1 compared to holo-IDO1^47^ (Extended Data Fig. 5f), the observed conformational rearrangements represent a novel and unique binding mode of the iDegs.

Mechanistically, because iDegs bind deep in the active site, it is unlikely that they directly contact an E3 ligase. Instead, iDeg-induced degradation possibly involves increased accessibility of the C-terminal K-helix bearing the ubiquitinated K389, as compared to the conformations observed for all published IDO1 inhibitors.

**Fig. 4:**
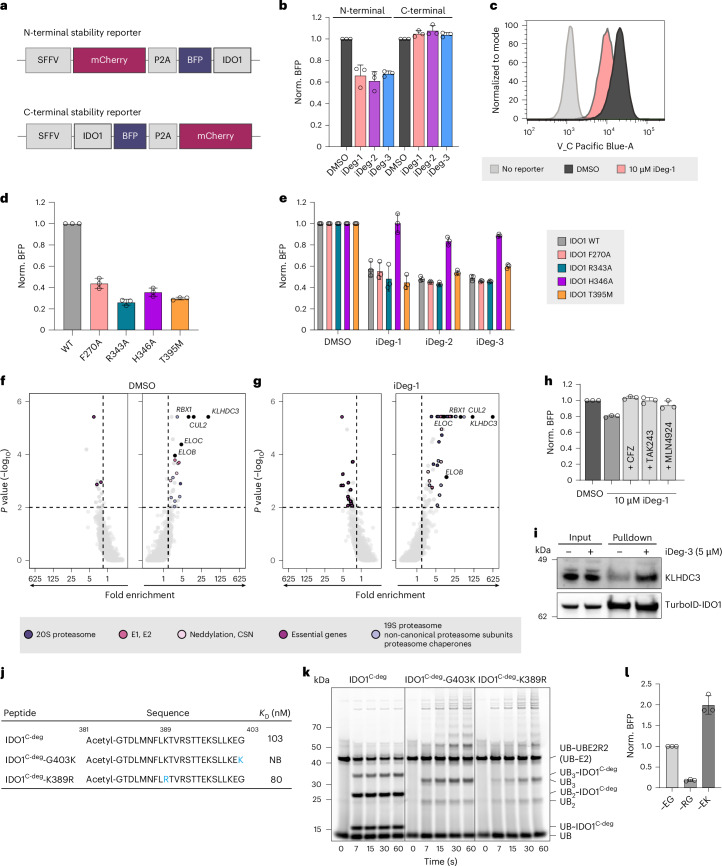
KLHDC3 is involved in iDeg-mediated IDO1 degradation. **a**, IDO1 stability reporter design. **b**, IDO1 levels detected by IDO1 stability reporters. KBM7 IDO1 reporter cells were treated with iDeg-1, iDeg-2 or iDeg-3 (1 µM) for 24 h before detection of IDO1 levels using flow cytometry. Normalized BFP-to-mCherry ratios (norm. BFP) were calculated per genotype, respectively. Data are presented as mean values ± s.d.; *n* = 3 biological replicates. **c**, Representative histogram for the iDeg-1-mediated depletion of BFP-IDO1 (24 h, 10 µM). **d**, Influence of IDO1 mutations on BFP-IDO1 protein levels. Data are presented as mean values ± s.d.; *n* = 3 biological replicates. **e**, Influence of IDO1 mutants on the degradation efficiency of iDegs (24 h, 10 µM). Data are presented as mean values ± s.d.; *n* = 3 biological replicates. **f**, Identification of genes required for native IDO1 degradation. **g**, Identification of genes required for iDeg-1-mediated IDO1 degradation. CSN, COP9 signalosome. In **f** and **g**, genes are highlighted for *P* < 0.05 (one-sided MAGeCK) and log_2_(fold-change, FC) > 1.585 (*n* = 2 biological replicates). **h**, IDO1 depletion is rescued by 10 h of co-treatment with either CFZ, TAK243 or MLN4924 (1 µM each). Data are presented as mean values ± s.d.; *n* = 3 biological replicates. **i**, Proximity labelling and enrichment of biotinylated KLHDC3 in HEK293T cells expressing IDO1–TurboID biotin ligase. The treatment time with iDeg-3 or DMSO was 2.5 h. A representative immunoblot is shown for *n* = 2 biological replicates, and 2% of the total protein input used for the pulldown was loaded in the input lane. **j**, KLHDC3 affinity towards WT (IDO1^C-deg^) and mutant (IDO1^C-deg^-G403K and IDO1^C-deg^-K389R) IDO1 C-terminal peptides determined using ITC. NB, no binding. **k**, Fluorescent ubiquitin transfer from neddylated CRL2^KLHDC3^-activated UBE2R2 to the indicated C-terminal IDO1 peptides over time. **l**, IDO1 stability reporter variants in KBM7 cells measured by flow cytometry and depicted normalized to the WT (that is, –EG) reporter. Data are presented as mean values ± s.d.; *n* = 3 biological replicates. See also Supplementary Fig. 1. Source data

To explore the contribution to IDO1-induced degradation of several amino acids that adopt substantially shifted positions in the iDeg-bound structures, we generated an IDO1 stability reporter in KBM7 cells harbouring an inducible Cas9 cassette^49^ (Fig. 4a). Supporting a role of the C-terminal region of IDO1 in degradation, only N-terminally fused BFP enabled iDeg-induced reporter degradation (Fig. 4b). Alanine substitutions were introduced at H346, R343 and F270, which interact with haem in the holo-IDO1 structure^50^ and adopt different conformations in the iDeg-bound state compared to all other published IDO1 structures. In addition, we tested the T395M mutation, as T395 located in the K-helix contributes to its stabilization through hydrogen bonding with R343 (J-helix) and Q281 (E–F loop). All four mutants displayed lower protein stability in cells, indicating their role in maintaining a stable IDO1 conformation (Fig. 4d). Degradation induced by iDeg-1, iDeg-2 or iDeg-3 in cells expressing these IDO1 mutants was rescued only by the H346A mutation, highlighting their interaction with H346 as being crucial for degradation (Fig. 4e).

### iDegs promote IDO1 ubiquitination by KLHDC3

**Extended Data Fig. 6:**
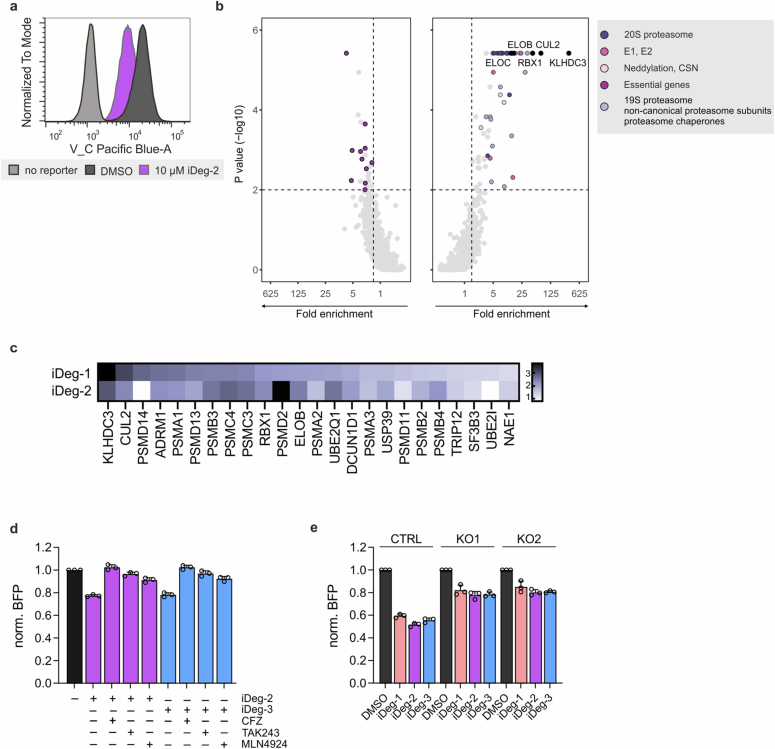
iDeg-mediated IDO1 depletion is dependent on KLHDC3. **a**, Representative flow-cytometric histogram for iDeg-2 mediated depletion of BFP-IDO1 (24 h, 10 µM) in KBM7 iCas9 cells. **b**, Identification of genes required for iDeg-2-mediated IDO1 degradation. CSN: COP9 signalosome. **c**, Heatmap of the gene enrichment in the CRISPR screen calculated as fold-change between the DMSO control and iDeg-1 and iDeg-2 treatment. **d**, Detection of IDO1 levels using the IDO1 stability reporter. KBM7 IDO1 reporter cells were treated with iDeg-2 or 3 (10 µM) +/- indicated co-treatments for 10 h prior to detection of IDO1 levels using flow cytometry. CFZ: 1 µM; TAK243: 1 µM, MLN4324: 1 µM. Mean values ± SD, n = 3 biological replicates. **e**, IDO1 protein levels in KBM7-BFP-IDO1 CTRL or KBM7-BFP-IDO1 KLHDC3 KO1 or KO2 cells upon treatment with iDeg-1, 2 or 3 (1 µM) for 24 h. Values were normalized to the respective DMSO control, which was set to 1. Mean values ± SD, n = 3 biological replicates. Source data

To identify the E3 responsible for iDeg-mediated IDO1 degradation, we conducted a fluorescence-activated cell sorting (FACS)-based CRISPR–Cas9 screen using an sgRNA library targeting 1,301 ubiquitin-associated genes (six sgRNAs per gene)^51^. Cas9 expression was induced for 72 h before 14 h of compound treatment, then the cells were enriched for increased or decreased BFP levels using FACS and the corresponding sgRNAs quantified by deep sequencing (Fig. 4f,g, Extended Data Fig. 6b and Supplementary Fig. 1), revealing the genes functionally required for iDeg-induced degradation.

As expected, knockout of proteasome subunits or of genes involved in neddylation counteracted iDeg activity (Fig. 4f,g and Extended Data Fig. 6b,c), thus phenocopying the IDO1 stability reporter behaviour upon chemical perturbation of the UPS (Fig. 4h and Extended Data Fig. 6d). Importantly, we further identified the cullin-RING ligase (CRL) complex, including cullin2 (CUL2), RBX1, elongin B/C (EloB and EloC) and the Kelch domain containing protein 3 (KLHDC3), as required for IDO1 degradation.

Unexpectedly, genetic disruption of the CRL2^KLHDC3^ complex also affected baseline IDO1 turnover under vehicle (dimethyl sulfoxide, DMSO) treatment. In the presence of iDegs, however, knockout of these genes led to an even higher enrichment of the corresponding sgRNAs (Fig. 4g and Extended Data Fig. 6b,c). This indicated that the compounds could function by enhancing the efficiency of IDO1 degradation. To validate these findings, we employed the TurboID approach and expressed IDO1 fused to the TurboID biotin ligase in HEK293T cells followed by enrichment of biotinylated proteins. Indeed, KLHDC3 was slightly biotinylated in the control condition, which increased upon treatment of cells with iDeg-3 (Fig. 4i), thus confirming that IDO1 and KLHDC3 interact in cells, and that this interaction is enhanced in the presence of the degrader. Contrary to classical degrader modalities such as PROTACs or molecular glue degraders, which typically function by inducing proximity between an E3 and a target that is functionally inconsequential in the absence of the small molecule, iDegs thus appear to promote a native route for IDO1 turnover.

### IDO1 is a natural substrate of CRL2^KLHDC3^

The CRL substrate receptor KLHDC3 has not yet been employed for small-molecule-induced protein degradation. KLHDC3 recognizes C-degrons^52,53^, which explains why the C-terminally fused IDO1-BFP reporter was not degraded, but the BPF-IDO1 reporter was (Fig. 4b). The C-terminal EG sequence of human IDO1 is consistent with the distinguishing feature of a KLHDC3 C-degron, which is a C-terminal glycine^52–54^. In agreement, a peptide IDO1^C-deg^ corresponding to the C-terminal region (residues 381–403) bound KLHDC3 with a *K*_D_ of 103 nM (Fig. 4j and Supplementary Fig. 2a). Substituting the C-terminal glycine with lysine (peptide IDO1^C-deg^-G403K) abolished binding to KLHDC3. Replacing the ubiquitination site (peptide IDO1^C-deg^-K389R) did not impact E3-degron complex formation (Fig. 4j and Supplementary Fig. 2b).

To test whether IDO1 is a direct substrate of CRL2^KLHDC3^, we biochemically reconstituted ubiquitination. Because KLHDC3-EloB/C can form an autoinhibited self-assembly^55^, we used a monomeric version of KLHDC3 (C-terminal Gly-to-Lys mutant). Assays were performed in the ‘pulse-chase’ format. The pulse reaction generates a thioester-linked E2 conjugate with fluorescently labelled ubiquitin. Next, E3 and substrate are added, and fluorescent ubiquitin transfer from E2 (UBE2R2) to the substrate and subsequently to a substrate-linked ubiquitin is observed over time. The IDO1^C-deg^ peptide was ubiquitinated in vitro in a CRL2^KLHDC3^-dependent manner, whereas the mutant versions were not (Fig. 4k and Supplementary Fig. 2b). In cells, mutation of the IDO1 C-terminal glycine increased abundance, whereas mutation of the non-optimal –EG degron to an optimal –RG C terminus reduced IDO1 abundance (Fig. 4l). These findings demonstrate that IDO1 is a substrate of KLHDC3 and that the C-terminal glycine-based degron is essential for interaction with the E3 ligase.

**Fig. 5:**
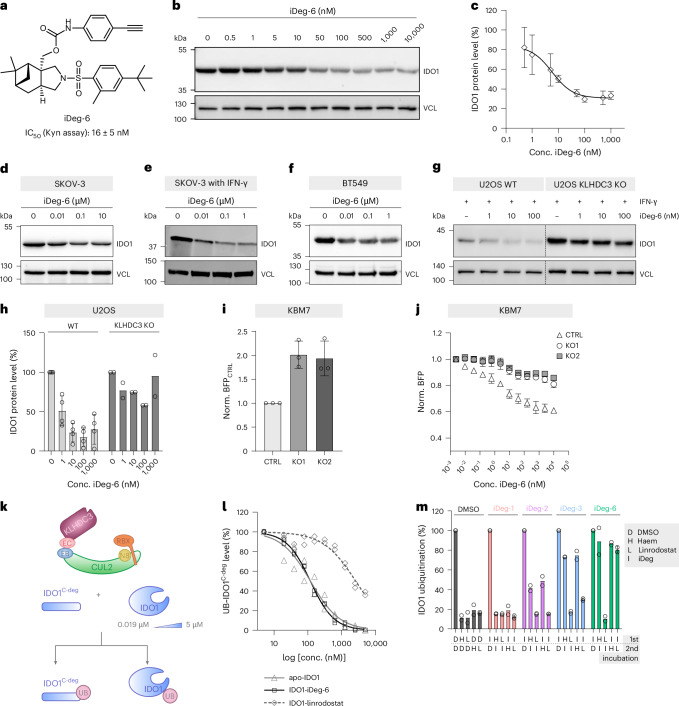
Validation of KLHDC3 as an E3 ligase regulating IDO1. **a**, Structure of iDeg-6 and the IC_50_ value in a Kyn assay in BxPC3 cells. Data are presented as mean value ± s.d.; *n* = 3 biological replicates. **b**,**c**, Reduction of IDO1 protein levels by iDeg-6. Degradation efficiency was assessed in a modified set-up including IFN-γ washout before compound addition. BxPC3 cells were treated with 50 ng ml^−1^ IFN-γ for 24 h before washout, addition of iDeg-6 for 24 h, then immunoblotting (**b**) and quantification of the IDO1 protein levels (**c**) from **b**. Data are presented as mean values ± s.d.; *n* = 4 biological replicates, except for 50 and 500 nM (*n* = 3 biological replicates). **d**–**f**, IDO1 levels in SKOV-3 (**d**), SKOV-3 cells stimulated with IFN-γ for 24 h followed by a washout (**e**) or BT549 (**f**) cells. Cells were treated with iDeg-6 for 24 h before immunoblotting. Representative immunoblots are shown for *n* = 3 biological replicates for SKOV-3 and SKOV-3 with IFN-γ or *n* = 4 biological replicates for BT549. **g**,**h**, IDO1 protein levels in wild-type (WT) U2OS or KLHDC3 knockout (KO) U2OS cells. Cells were stimulated with 5 ng ml^−1^ IFN-γ for 24 h before washout, treatment with iDeg-6 or DMSO for 24 h, and immunoblotting (**g**). Representative data of *n* = 4 biological replicates (U2OS WT) or *n* = 2 biological replicates (KO cells) are shown. **h**, Quantification of the band intensities from **g** and Extended Data Fig. 8h. Data are presented as mean values ± s.d.; *n* = 3 biological replicates (U2OS WT) or *n* = 2 biological replicates (U2OS-KLHDC3 KO). **i**,**j**, IDO1 protein levels in KBM7-BFP-IDO1 (CTRL) or KBM7-BFP-IDO1 KLHDC3 KO1 or KO2 cells in the absence (**h**, normalized to CTRL) or presence of iDeg-6 (**i**, normalized to the respective genotype). The treatment time was 24 h. Data are presented as mean values ± s.d.; *n* = 3 biological replicates. **k**, Schematic representation of the IDO1 competition ubiquitination assay. EB, elongin B; EC, elongin C; N8, neddylation; UB, ubiquitin. **l**, Quantification of KLHDC3-dependent IDO1 C-terminal peptide ubiquitination (IDO1^C-deg^) upon titrating increasing concentrations of competing full-length apo-IDO1, iDeg-6-IDO1 or linrodostat-bound IDO1 to measure IC_50_ values. *n* = 2 independent experiments. **m**, Apo-IDO1 ubiquitination after two sequential 20-min incubations (first and second) with 42 µM of each indicated compound, followed by the addition of NEDD8-CUL2^KLHDC3^ to initiate ubiquitination. Data are presented as mean values; *n* = 2 independent experiments. See also Supplementary Fig. 4. Source data

**Extended Data Fig. 7:**
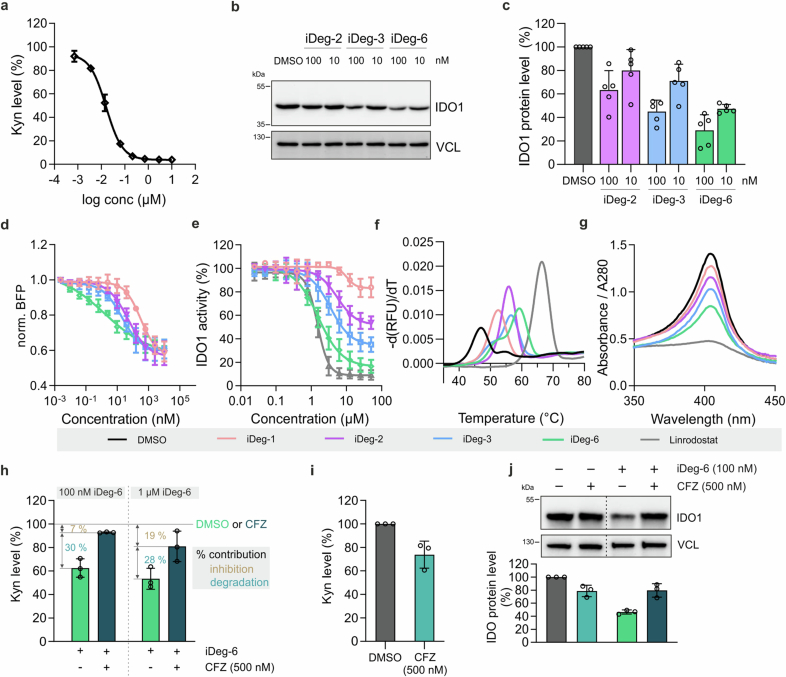
Characterization of iDeg-6. **a**, Kyn assay in BxPC3 cells after treatment with iDeg-6 and IFN-γ, respectively, for 48 h prior to detection of Kyn levels utilizing *p*-DMAB. Mean values ± SD, n = 3 biological replicates. **b,c**, IDO1 protein levels in BxPC3 cells upon treatment with IFN-γ for 24 h followed by washout and addition of iDeg-2, 3 or iDeg-6 for 24 h. Representative immunoblot (one of n = 5 biological replicates) for IDO1 and vinculin as a loading control. **c**, Quantified band intensities from b. Mean values ± SD, n = 5 biological replicates. **d**, Reduction of IDO1 levels determined in the BFP-IDO1 reporter cell line. Mean values ± SD, n = 3 biological replicates. **e**, *In vitro* IDO1 enzymatic activity assay in presence of iDeg-1-6 or linrodostat. rhIDO1 was pre-incubated with the compounds at 37 °C for 90 min prior to detection of Kyn levels using *p*-DMAB. Mean values ± SD, n = 3 independent experiments. **f**, rhIDO1 thermal stability in presence of iDeg-1, 2, 3 or iDeg-6 (50 µM), or DMSO or linrodostat (50 µM) determined by means of nanoDSF. rhIDO1 and the compounds were pre-incubated for 3 h at 37 °C prior to the measurement. Representative data of n = 3 independent experiments. **g**, Detection of haem-bound IDO1 by means of UV/Vis spectroscopy in presence of iDeg-6 (100 µM) as compared to iDeg-1, 2 and 3, DMSO or linrodostat (100 µM). Incubation at 37 °C for 3 h. Representative data (n = 3 independent experiments). **h-j**. Contribution of IDO1 degradation to the reduction in Kyn levels. BxPC3 cells were stimulated with IFN-γ for 24 h followed by a washout and co-treatment with carfilzomib (CFZ, 500 nM) and iDeg-6 (100 nM or 1 µM) for 7 h. Kyn levels (**h-i**) and IDO1 protein levels (**j**) were determined. Kyn levels were normalized to the respective control (DMSO or CFZ alone in h and DMSO in i). Mean values ± SD, n = 3 biological replicates. VCL: vinculin. Source data

To benchmark the degradation potency of iDeg-1, iDeg-2 and iDeg-3 and subsequently the identified inhibitor and degrader iDeg-6 (IC_50_ of 16 ± 5 nM in the Kyn assay; Fig. 5a and Extended Data Fig. 7a), IDO1 levels were detected after stimulation of cells for 24 h with IFN-γ followed by a washout (to avoid IDO1 expression) and compound addition. iDeg-6 most potently depleted IDO1 in the cells, with a maximal achievable degradation (*D*_max_) of 70% at 100 nM and a half-maximal degradation concentration (DC_50_) for IDO1 degradation of 6.5 ± 3 nM (Fig. 5a–c and Extended Data Fig. 7b,c). Also, in the BFP-IDO1 reporter cell lines iDeg-1 was the least and iDeg-6 the most potent compound (Extended Data Fig. 7d). In vitro iDeg-6 inhibited IDO1 activity nearly completely and more potently than iDeg-1 to 3 with an IC_50_ of 1.6 ± 0.3 µM (Extended Data Fig. 7e). Direct binding of iDeg-6 to IDO1 was detected using isothermal titration calorimetry (ITC; Supplementary Fig. 3). The compound induced thermal stabilization of the protein and haem displacement to a higher extent as compared to iDeg-1, iDeg-2 and iDeg-3 (Extended Data Fig. 7f,g). We therefore used iDeg-6 for further validation.

We estimated the contribution of IDO1 inhibition and IDO1 degradation by iDeg-6 to the total reduction of Kyn levels in the presence or absence of the proteasome inhibitor CFZ (Extended Data Fig. 7h–j and Supplementary Table 3). IDO1 degradation accounted for 81% or 59% of the achieved Kyn level decrease at 100 nM or 1 µM, respectively. However, the cellular response to proteasomal inhibition is rather complex and can induce compensatory degradation mechanisms that can impact total IDO1 levels.

**Extended Data Fig. 8:**
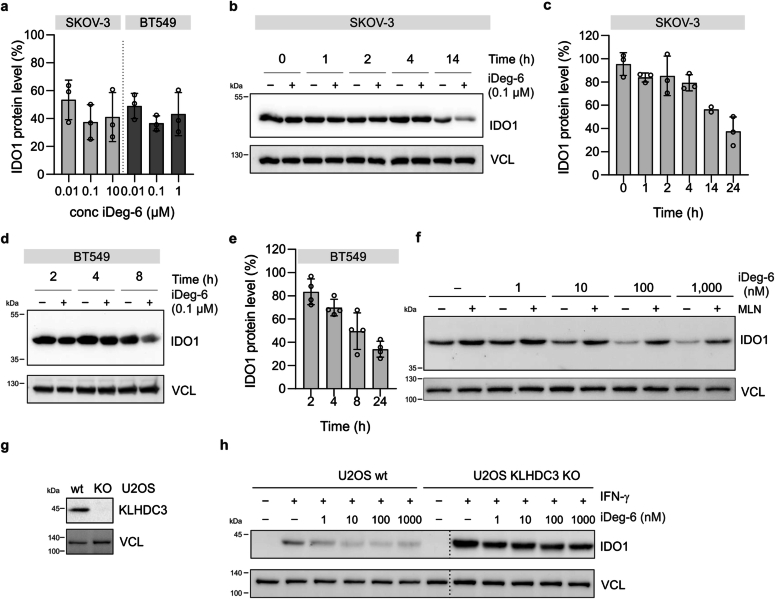
Influence of iDeg-6 on IDO1 protein levels in different cell lines. **a**, IDO1 protein levels in SKOV-3 and BT549 cells. Quantification of band intensities from Fig. 5d and f. Mean values ± SD, n = 3 biological replicates. **b-e**, IDO1 protein levels in SKOV-3 (b and c) or BT549 cells (d and e) after treatment with iDeg-6 for different time. IDO1 and vinculin (VCL) were detected by means of immunoblotting. Quantification of band intensities from b and d is shown in c and e, respectively. Mean values ± SD, n = 3 biological replicates except for 14 h (n = 2 biological replicates for SKOV-3 and n = 4 biological replicates for BT549). **f**, Influence of neddylation inhibition on IDO1 protein levels in BxPC3 cells. BxPC3 cells were stimulated with IFN-γ for 24 h prior to washout, treatment with 500 nM MLN4924 (MLN) and iDeg-6 for 16 h and immunoblotting. Representative blot (one of n = 3 biological replicates). **g**, Detection of CRISPR–Cas9-mediated KLHDC3 knockout in U2OS cells. Levels of KLHDC3 and vinculin were detected by means of immunoblotting. Representative blot (one of n = 3 biological replicates). **h**, IDO1 protein levels in U2OS and U2OS cells carrying a KLHDC3 knockout. Representative blot (one of n = 3 biological replicates). VCL: vinculin. Source data

**Extended Data Fig. 9:**
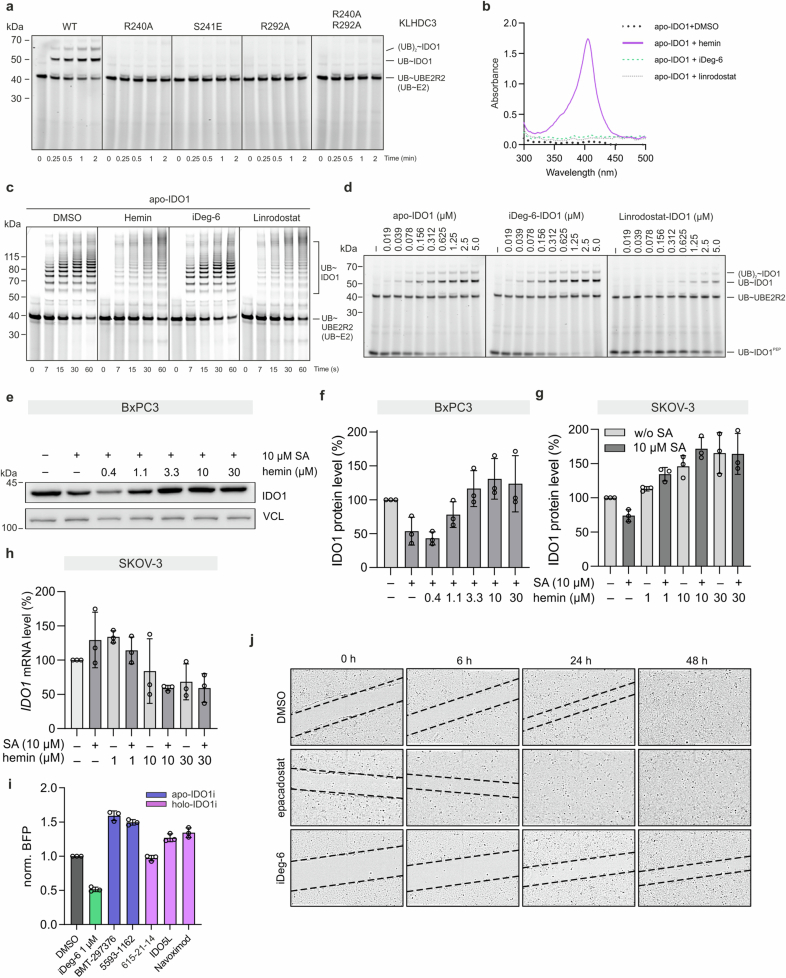
Ubiquitination of IDO1 by CRL2^KLHDC3^ and influence of succinyl acetone (SA) and hemin on IDO1 levels. **a**, Fluorescent scan of pulse-chase assay monitoring ubiquitination of iDeg-6-IDO1 by KLHDC3 mutants. iDeg6-IDO1 was incubated with CRL2^KLHDC3^ E3 complex and ubiquitination reactions were initiated by the addition of the pre-formed thioester-linked E2~ubiquitin conjugate (UBE2R2 ~ UB) with the ubiquitin fluorescently-labeled. UB: ubiquitin. **b**, Detection of haem load for the conditions in c detected by means of UV/Vis spectroscopy. UB: ubiquitin. **c**, Fluorescent scan of pulse-chase assay monitoring ubiquitination of apo-IDO1 with or without supplementation with small molecules by CRL2^KLHDC3^ (**b**). Apo-IDO1 was incubated with the indicated compounds (30 µM) for 90 min at 37 °C prior to incubation with CRL2^KLHDC3^ E3 complex. Ubiquitination reactions were initiated by the addition of the pre-formed thioester-linked E2~ubiquitin conjugate (UBE2R2 ~ UB) with the ubiquitin fluorescently-labeled. Representative data for n = 2 independent experiments. **d**, KLHDC3-dependent IDO1 C-terminal peptide ubiquitination (IDO1^C-deg^) upon titrating increasing concentrations of competing full-length apo-IDO1, iDeg-6-IDO1, or linrodostat-bound IDO1 to measure IC_50_ values (n = 2 independent experiments). **e,f**, BxPC3 cells were treated with 50 ng/mL IFN-γ with or without 10 µM SA for 24 h followed by the addition of hemin for further 24 h. **f**, Quantification of band intensities from e. Mean values ± SD, n = 3 biological replicates. VCL: vinculin. **g**, Quantification of band intensities from Fig. 6d. SKOV-3 cells were treated with SA for 48 h prior to the addition of hemin for another 24 h and immunoblotting (see Fig. 6d). Mean values ± SD, n = 3 biological replicates. **h**, Detection of *IDO1* mRNA levels in SKOV-3 cells upon treatment with succinyl acetone (SA) for 24 h followed by addition of hemin for another 24 h. Mean values ± SD (n = 3 biological replicates). **i**, Influence of selected IDO1 inhibitors (10 µM) in comparison to iDeg-6 (1 µM) on IDO1 protein levels in KBM7-BFP-IDO1 cells. Cells were treated with the compounds for 24 h followed by quantification of IDO1 using flow cytometry. Mean values ± SD, n = 3 biological replicates. **j**, Wound healing assay using SKOV-3 cells in the presence of epacadostat (1 µM), iDeg-6 (1 µM) or DMSO as a control. Prior to performing the wound healing assay, cells were pre-conditioned for 24 h with the same concentrations of the compounds. For each time point, one representative image of three is shown. Source data

iDeg-6 also depleted IDO1 protein in SKOV-3 and BT549 cells in the absence of or upon stimulation with IFN-γ in a dose- and time-dependent manner (Fig. 5d–f and Extended Data Fig. 8a–e). The neddylation inhibitor MLN4924 rescued iDeg-6-induced IDO1 depletion (Extended Data Fig. 8f) and increased IDO1 in the absence of iDegs, demonstrating that neddylation is required for both native and iDeg-induced IDO1 degradation. KLHDC3 knockout in U2OS or KBM7-BFP-IDO1 cells increased IDO1 amounts and rescued iDeg-6-dependent degradation (Fig. 5g–j and Extended Data Fig. 8g,h; also Extended Data Fig. 6e for iDeg-1, iDeg-2 and iDeg-3). IDO1 ubiquitination in the presence of iDeg-6 was abolished for KLHDC3 variants carrying mutations in the degron recognition motif (R240A, S241E, R292A)^54^ (Extended Data Fig. 9a).

Surprisingly, in vitro, apo-IDO1 was rapidly ubiquitinated by CRL2^KLHDC3^. IDO1 was also ubiquitinated upon treatment of apo-IDO1 with iDeg-6 (Extended Data Fig. 9b,c). In contrast, both haem and the apo-IDO1 inhibitor linrodostat suppressed IDO1 ubiquitination under these conditions (Extended Data Fig. 9b,c). Hence, biochemically, apo-IDO1 is a better KLHDC3 substrate than holo-IDO1, consistent with structural data showing haem and lindrodostat determining the arrangement of the K-helix, as well as quantitative insights from competitive ubiquitination assays. Briefly, ubiquitination of IDO1 C-terminal peptide (IDO1^C-deg^) was examined while titrating apo-IDO1 or compound-bound IDO1. If a full-length IDO1 binds KLHDC3, then substrate preference shifts from peptide to protein along the titration, allowing calculation of IC_50_ values for inhibiting C-terminal peptide ubiquitination. Apo-IDO1 and iDeg-6-bound IDO1 show IC_50_ values of 119 and 118 nM, respectively, matching the affinity towards the isolated C-terminal peptide measured by ITC (Figs. 4j and 5k,l and Extended Data Fig. 9d). In contrast, the IDO1 bound to linrodostat showed a >20-fold greater IC_50_ value (2.6 µM). Thus, apo-IDO1 and iDeg-6-bound IDO1 are high-affinity KLHDC3 substrates, whereas linrodostat binding renders IDO1 less suitable for ubiquitination.

Next, ubiquitination assays were designed to elucidate the biochemical function of the iDegs. Apo-IDO1 was incubated first with an iDeg, or first with hemin or linrodostat (which secure the IDO1 C-terminal helix). Afterwards, hemin or linrodostat were added to the iDeg-treated IDO1, or vice versa (Fig. 5m and Supplementary Fig. 4). Although apo-IDO1 and iDeg-bound IDO1 were readily ubiquitinated in the absence of haem, when the iDeg treatment was first, ubiquitination proceeded after haem addition. Due to its superior occupation of the haem-binding pocket, linrodostat prevented ubiquitination in all assays if added first. Although iDeg-1 showed little effect in these assays, iDeg-2 through iDeg-6 increasingly competed with haem and allowed ubiquitination, trending with their IC_50_ towards IDO1 activity in vitro and efficacy at eliciting IDO1 degradation in cells.

**Fig. 6:**
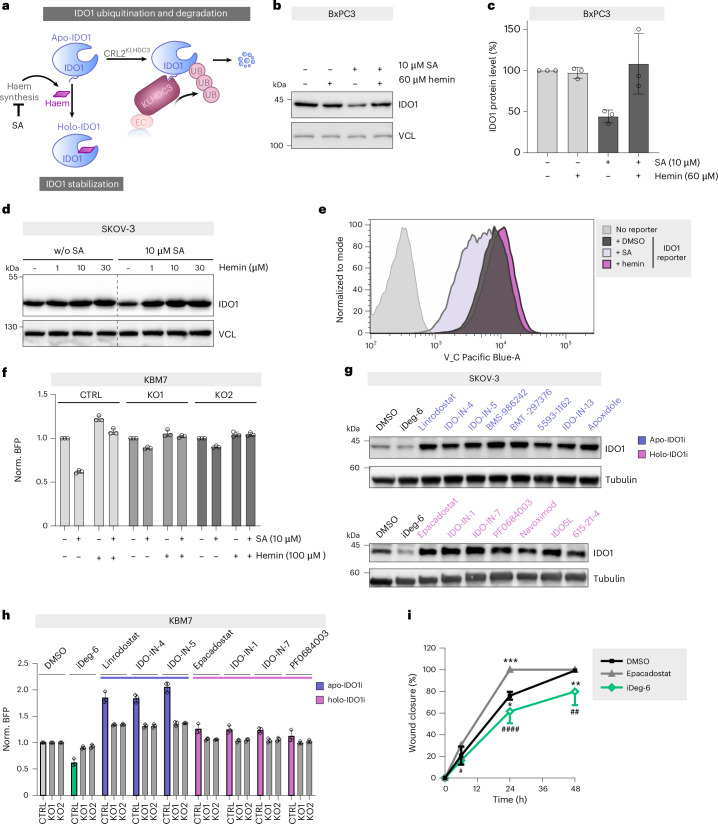
Apo-IDO, not holo-IDO1, is the preferential substrate for degradation. **a**, Regulation of IDO1 by haem synthesis and succinyl acetone (SA) as an inhibitor of haem synthesis. **b**,**c**, Influence of SA and haem on IDO1 protein levels in BxPC3 cells, detected using immunoblotting. Cells were treated with 50 ng ml^−1^ of IFN-γ with or without 10 µM SA for 24 h, followed by the addition of hemin for a further 24 h. **c**, Quantification of the band intensities from **b**. Data are presented as mean values ± s.d.; *n* = 3 biological replicates. **d**, Influence of SA and haem on IDO1 levels in SKOV-3 cells. Cells were treated with SA for 48 h before the addition of hemin for another 24 h and immunoblotting. Representative data of *n* = 3 biological replicates are shown. **e**,**f**, Influence of SA and hemin on IDO1 abundance in KBM7-BFP-IDO1 (**e**) or KBM7-BFP-IDO1 KLHDC3 KO cells (**f**). Cells were pre-treated with 10 µM SA for 24 h followed by a treatment for 48 h with 100 µM hemin or DMSO as a control. IDO1 levels were quantified using flow cytometry. Data are presented as mean values ± s.d.; *n* = 3 biological replicates. **g**, Influence of known apo-IDO inhibitors (apo-IDO1i) and holo-IDO1 inhibitors (holo-IDO1i) on IDO1 protein levels in SKOV-3 cells. Cells were incubated with the compounds (5 µM) for 48 h before immunoblotting. Representative data of *n* = 3 biological replicates are shown. **h**, Influence of selected IDO1 inhibitors (IDO1i, 1 µM) on IDO1 protein levels in KBM7-BFP-IDO1 or KBM7-BFP-IDO1 KLHDC3 KO cells. Cells were treated with the compounds for 24 h, followed by quantification of IDO1 using flow cytometry. Data are presented as mean values ± s.d.; *n* = 3 biological replicates. **i**, Influence of iDeg-6 on wound closure using SKOV-3 cells in the presence of epacadostat (1 µM), iDeg-6 (1 µM) or DMSO as control. Before performing the wound healing assay, cells were pre-conditioned for 24 h with the same compound concentration. Data are displayed as the percentage of wound closure with respect to time 0. Data are presented as mean values ± s.d.; *n* = 3 biological replicates. The data were analysed by two-way analysis of variance (ANOVA) followed by a post hoc Bonferroni’s test. Statistical analysis of treated versus DMSO control is indicated by asterisks (*P = 0.0368, **P = 0.004, ***P = 0.0005), and comparison between epacadostat- and iDeg-6-treated cells is indicated by hash symbols (^#^*P* = 0.0415, ^##^*P* = 0.0028, ^####^*P* < 0.0001). See Extended Data Fig. 9j for the corresponding images. Source data

These findings suggest a possible model for the regulation of IDO1. De novo translated apo-IDO1 is rapidly cleared in cells by degradation mediated by KLHDC3. In the presence of haem, apo-IDO1 is charged with haem to form holo-IDO1, and the holo-pool may thus escape degradation. Support for such a mechanism in cells (Fig. 6a) was obtained by treatment with haem synthesis inhibitor succinyl acetone to deplete the haem and shift the equilibrium to the apo-IDO1 form. In IFN-γ-stimulated BxPC3 cells, succinyl acetone reduced IDO1 protein levels, and the addition of hemin dose-dependently increased IDO1 (Fig. 6b,c and Extended Data Fig. 9e,f).

Similar observations were made in SKOV-3 cells and KBM7-BFP-IDO1 stability reporter cells (Fig. 6d,e and Extended Data Fig. 9g). Addition of only hemin dose-dependently increased IDO1 protein levels in SKOV-3 cells, which is in agreement with the majority of IDO1 being in the apo-form^19^. Changes in the IDO1 protein level did not result from altered *IDO1* mRNA levels (Extended Data Fig. 9h). Knockout of KLHDC3 counteracted succinyl acetone-mediated IDO1 depletion, indicating that apo-IDO1 degradation is dependent on KLHDC3 (Fig. 6f). These results support involvement of KLHDC3 in the regulation of apo-IDO1 turnover, both under native conditions and in the presence of iDegs. The mechanism of action relies on further pushing IDO1 towards the degradation-sensitive state.

**Extended Data Fig. 10:**
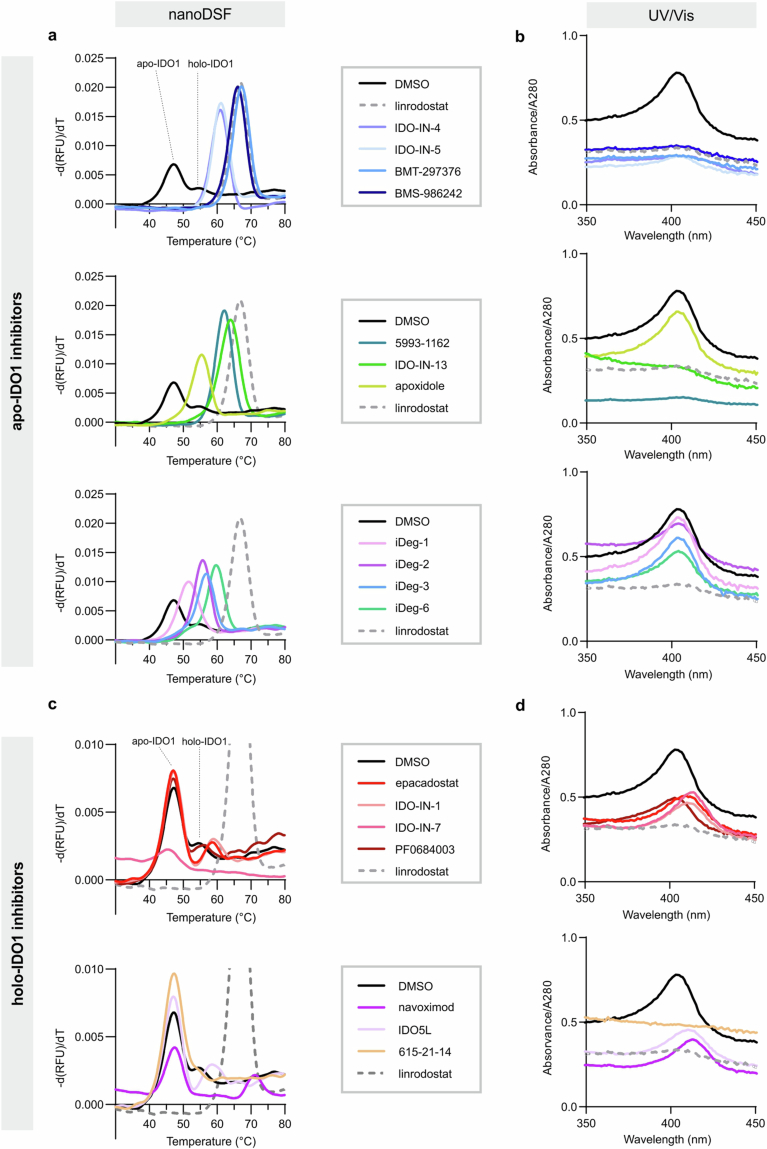
Influence of apo- and holo-IDO1 inhibitors on the melting behavior and haem binding of IDO1. **a** and **c**, rhIDO1 thermal stability in presence of 50 µM of the compounds determined by means of nanoDSF. rhIDO1 and the compounds were pre-incubated for 3 h at 37 °C prior to the measurement. Representative data of n = 3 independent experiments. **b** and **d**, Detection of haem-bound IDO1 by means of UV/Vis spectroscopy in presence of the compounds (50 µM). Incubation at 37 °C for 3 h. Representative data of n = 3 independent experiments. Source data

We then asked whether other IDO1 inhibitors might control IDO1 abundance. All investigated inhibitors increased IDO1 protein levels in the cells, which was partially reverted in KLHDC3 knockout cells (Fig. 6g,h and Extended Data Fig. 9i). These findings are in agreement with the biochemical data showing lower ubiquitination of IDO1 in the presence of linrodostat (Fig. 5m), and our structural data showing iDegs bind a distinct conformation of IDO1. Hence, the IDO1-KLHDC3 degradation circuit can be modified in both directions by liganding common residues, albeit with distinct overall structural consequences. We analysed the influence of these IDO1 inhibitors on the melting temperature of IDO1 and on haem displacement to assess whether these assays could be used for predicting IDO1 degrader activity. Apo-IDO1 inhibitors shifted the melting curve to higher temperatures, whereas most holo-IDO1 inhibitors shifted the peak corresponding to the haem-bound state (Extended Data Fig. 10). Both types of inhibitors affected the UV–vis absorption: holo-IDO1 inhibitors shifted the absorbance peak to a higher wavelength, whereas apo-IDO1 inhibitors decreased the absorbance at the Soret band. These biophysical assays, however, cannot be used to predict IDO1 degraders as iDegs change the *T*_m_ and the Soret band intensity in a similar manner as other apo-IDO1 inhibitors.

Finally, we assessed the ability of iDeg-6 to suppress the non-enzymatic activity of IDO1, which has recently been shown to promote tumour proliferation and migration^33,34^. Specifically, in SKOV-3 cells, IDO1 enzymatic inhibition by epacadostat stabilizes a non-enzymatic IDO1 protein, which results in faster cell migration. In contrast, IDO1 knockdown decreases the migratory capacity of SKOV-3 cells^34^. We thus compared cell migration, using a wound healing assay, with SKOV-3 cells exposed to iDeg-6 and epacadostat. iDeg-6 significantly slowed down wound closure compared to the control and cells that were treated with epacadostat, demonstrating that iDegs can also affect the non-enzymatic protumorigenic function of IDO1 (Fig. 6i and Extended Data Fig. 9j).

## Discussion

Clinical investigation of IDO1 inhibition has had limited success so far^47,56^, possibly due to its non-enzymatic signalling function^29,32^. This limitation could be addressed by the targeted degradation of IDO1. We identified a pseudo-natural product class termed iDegs that potently induce IDO1 ubiquitination and degradation through the native degradation pathway mediated by the E3 ligase CRL2^KLHDC3^. iDegs bind to apo-IDO1, which is either present in cells or generated by intrinsic haem loss, leading to loss of enzymatic activity. More importantly, iDegs induce a conformational shift of the C-terminal helix and lock IDO1 in a state favouring ubiquitination by KLHDC3. As degraders, iDegs are unique, as other apo-IDO1 modulators increase IDO1 protein abundance. Our data demonstrate that apo-IDO1, but not haem-bound IDO1, is preferentially ubiquitinated and degraded, suggesting also that apo-IDO1 can readily adopt a ubiquitination-sensitive conformation. In contrast, haem binding prevents IDO1 turnover. Therefore, targeting the same binding pocket with the haem cofactor or small-molecule ligands has a completely opposite impact on the IDO1-KLHDC3 degradation circuit and IDO1 fate.

So far, degraders employing native degradation mechanisms have largely been overlooked, and only a few examples (targeting BCL6 or EZH2^57,58^) support the notion that monovalent degraders in a more general sense may regulate the native mechanisms of protein homeostasis for the respective targets^2^. Scholes and colleagues have identified supercharging of endogenous degradation pathways as a frequently observed mechanism of inhibitor-induced kinase degradation^59^. Functionally differentiated, iDegs represent a group of small molecules that act as a switch to induce an IDO1 conformational state that shifts the equilibrium to the degradation-sensitive state, thereby channelling the IDO1-iDeg complex to the native degradation mechanism. This route to protein degradation complements current degradation strategies that are based on PROTACs or MGDs, as observed for oestrogen and androgen receptors, and may also apply to other proteins^60,61^.

Compared to IDO1-directed PROTACs^37–39^, iDegs are smaller and more drug-like and hijack the native ligase, making them broadly applicable to all cells and tissues expressing IDO1.

Due to their dual mode of action, iDegs impair all IDO1 functions, and, unlike other apo-IDO1 inhibitors, they induce a decrease in protein levels. Because the non-enzymatic functions of the protein and an inhibitor-mediated increase in IDO1 concentration^32,38^ may be linked to failure of IDO1 inhibitors in the clinic, small-molecule-induced inhibition and degradation may open up alternative therapeutic opportunities^13–15^.

## Methods

### Material

BxPC3, HEK293T and HeLa cells were purchased from DSMZ, and BT549, SKOV-3 and U2OS cells were obtained from ATCC. U2OS KO cells were obtained from the St. Jude Center for Advanced Genome Engineering (CAGE). KBM7 iCas9 cells were a gift from J. Zuber (IMP, Vienna). Lenti-X 293T lentiviral packaging cells were obtained from Clontech. pCMV3-IDO1 was obtained from Sino Biological US. pSpCas9(BB)-2A-GFP (PX458) plasmid was a gift from Feng Zhang (Addgene 48138, ref. ^62^). pCMVR8.74 was a gift from D. Trono (Addgene 22036). pMD2.G was a gift from D. Trono (Addgene 12259). Fetal bovine serum (FBS), penicillin/streptomycin, anti-IDO1 (14-9750-80) and anti-vinculin (V9131) antibodies were obtained from Thermo Fisher. Anti-KLHDC3 (HPA030131) was obtained from Sigma-Aldrich. Anti-rabbit horseradish peroxidase (HRP; 7074) was obtained from Cell Signaling Technology. Anti-IDO1 (ab211017) and anti-β-actin (ab8227) antibodies were obtained from Abcam. Mouse Thy1.1 antibody was purchased from BioLegend. Anti-DOCK-8 (11622-1-AP) and anti-RHOBTB3 (13945-1-AP) were obtained from Proteintech. Anti-α-tubulin antibody (ab18251) was obtained from Abcam. HRP-conjugated secondary goat anti-rabbit antibody (31460) was obtained from Pierce Biotechnology. IRDye-conjugated secondary antibodies were obtained from LI-COR Biosciences. Dulbecco’s modified Eagle medium (DMEM), McCoy’s medium, sodium pyruvate and non-essential amino acids were obtained from PAN Biotech. Where indicated, DMEM was obtained from Gibco. Iscove’s modified Dulbecco’s medium (IMDM) was obtained from Gibco. L-tryptophan was obtained from Sigma. IFN-γ was purchased from PeproTech. Succinyl acetone was obtained from TCI. Doxycycline was obtained from PanReac AppliChem. IDO-IN-1 (19402) and IDO-IN-5 (33178) were obtained from Cayman Chemical. IDO-IN-4 (HY-18769), IDO-IN-7 (HY-13983), PF-06840003 (HY-101111), linrodostat (BMS 986205, HY-101560) and epacadostat (HY- 15689) were obtained from MedChem Express. IDO5L (HY-15683) and BMT-297376 (HY-139205) were obtained from Hycutelc. Navoximod (BD630231), BMS-986242 (BD01401323) and 615-21-4 (BD4825) were obtained from BLD. IDO-IN-13 (TM-T11616) was obtained from CymitQuimica, and 5593-1162 (CAS-no. 1024595-87-6; 5593-1162) from Chemdiv.

### Cell culture

BxPC3 cells were cultured in RPMI 1640 medium (PAN Biotech) containing 10% heat-inactivated FBS. BT549, HeLa and HEK293T cells were cultured in DMEM (PAN Biotech) supplemented with 10% FBS, sodium pyruvate and non-essential amino acids. SKOV-3 and U2OS WT and KO cells were cultured in McCoy’s 5A medium (PAN Biotech) containing 10% heat-inactivated FBS. Lenti-X 293T lentiviral packaging cells were cultured in DMEM (Gibco) supplemented with 100 U ml^−1^ penicillin/streptomycin and 10% heat-inactivated FBS. IDO1-deficient SKOV-3 cells were produced using the CRISPR–Cas9 system. For this, 100,000 cell well^−1^ in six-well plates were transfected using Lipofectamine 3000 with PX458 plasmid containing three separated sgRNA located in *IDO1*. sgRNA sequences were 5′-CACACGCTATGGAAAACTCC-3′; 5′-AGCCCACTTCTTCATCAATA-3′; 5′-TACCATATTGATGAAGAAGT-3′. After 48 h, the cells were sorted for single cells based on green fluorescent protein (GFP) positivity. Single clones were expanded and successful KO of *IDO1* was verified using immunoblotting. KBM7 iCas9 cells were cultured in IMDM supplemented with 100 U ml^−1^ penicillin/streptomycin, 10% heat-inactivated FBS and 1 mM L-tryptophan. Cell lines were maintained in a humidified atmosphere at 37 °C and 5% CO_2_. Regular mycoplasma testing confirmed cell lines as mycoplasma-negative.

### Automated Kyn screening assay

The Kyn screening assay was performed as previously described^21^. BxPC3 cells were seeded in phenol red-free RPMI 1640 medium in 1,536-well or 384-well plates (Greiner 782086, Corning 3770) 24 h before the addition of the compounds, IFN-γ (50 ng ml^−1^, PeproTech) and L-Trp (380 µM, Sigma-Aldrich). After 48 h of incubation, initial cell viability was accessed by the addition of Hoechst 33342. Afterwards, trichloroacetic acid (TCA, Sigma-Aldrich) was added to a final concentration of 7% and incubated for 10 min before a centrifugation step of 10 min at 1,620*g*. Kyn levels were determined by means of the Kyn sensor^21^ at a final concentration of 17.5 µM in sensor buffer (50 mM H_3_PO_4_ and 120 mM NaCl pH 1). Fluorescence intensity (excitation, 535 nm; emission, 595 nm) was measured using a Spectramax Paradigm reader (Molecular Devices). Data were normalized to the values for cells treated with DMSO. All HTS data were analysed with the Quattro Software Suite (Quattro Research).

### Manual Kyn assay

For the manual assay, BxPC3 or SKOV-3 cells were seeded in clear 96-well or 384-well plates, respectively, 24 h before compound treatment and IFN-γ (50 or 5 ng ml^−1^, respectively) and L-Trp (380 µM) addition, as described in ref. ^21^. At 48 h after treatment, TCA was added and Kyn levels were detected using 2% wt/vol of *para*-dimethylaminobenzaldehyde (*p*-DMAB; Ehrlich reagent) in acetic acid. The absorbance of the Ehrlich reagent was detected at 492 nm and at 650 nm as background control on a Spark multimode microplate plate reader (Tecan). Kyn levels were determined by subtracting the absorbance value at 650 nm and presented relative to the DMSO control. Dose–response curves and IC_50_ values were generated and fitted with GraphPad Prism 9.2 using a four-parameter variable-slope nonlinear regression curve fit.

For label-free Kyn detection, the cell supernatant was analysed by LC–MS/MS following the addition of 30% TCA. Kyn levels were measured by HPLC–MS/MS using an LTQ Velos Pro and Dionex HPLC system (Thermo Fisher Scientific). Data were analysed using Thermo Xcalibur (Thermo Fisher Scientific) and presented with GraphPad Prism 9.2. Values are shown relative to the DMSO control.

### Live-cell analysis

SKOV-3 WT and IDO1-deficient cells were seeded in 48-well plates (8,000 cells well^−1^; Corning, 3548) for 2D experiments or in 96 ultralow-attachment plates (5,000 cells well^−1^, S-bio, MS-9096UZ) for 3D experiments. After seeding, cells were treated with 10 ng ml^−1^ (2D) or 1 ng ml^−1^ (3D) of IFN-γ and CellTox Green (Promega, G8731) and added to the culture medium (1:6,000). The following day, the cells were treated with either DMSO or 5 μM iDeg-1. iDeg-1 was freshly added every three days. The cells were monitored by live phase-contrast microscopy using an IncuCyte S3 system (Sartorius) with a 10x objective, capturing images every day up to four days (2D) and six days (3D). Cell death was quantified by counting the numbers of green fluorescence-positive cells (CellTox Green positive) normalized to the confluence of each respective image (2D) or by measuring the green signal mean intensity of each spheroid (3D).

### Immunoblotting

To analyse IDO1 levels, IFN-γ-stimulated BxPC3 cells (50 ng ml^−1^ IFN-γ), U2OS cells (5 ng ml^−1^ IFN-γ), HEK^rhIDO1^ cells (HEK293T cells electroporated with rhIDO1), SKOV-3 cells (stimulated with IFN-γ or not) and BT549 cells were treated with compounds for the indicated times followed by cell lysis (150 mM sodium chloride in 50 mM Tris pH 8.0, 1% NP-40 alternative, protease and phosphatase inhibitors). Protein concentrations were determined by a DC protein assay (Bio-Rad), followed by the addition of 1× sodium dodecyl sulfate (SDS) loading buffer and protein separation by 10% SDS–polyacrylamide gel electrophoresis (PAGE). Separated protein samples were transferred to a polyvinylidene difluoride (PVDF) membrane. IDO1 in IFN-γ-stimulated cells as well as vinculin, β-actin or α-tubulin as loading controls were detected by blocking the membranes with Intercept blocking buffer (LI-COR Biosciences, 927-70001) and using the primary antibodies anti-IDO1 (1:2,500, 14-9750-82, Thermo Fisher), anti-vinculin (1:10,000, V9131, Thermo Fisher), anti-β-actin (1:2,500, ab8227, Abcam) or anti-α-tubulin (1:2,500, ab18251, Abcam), and IRDye-conjugated secondary antibodies (IRDye 800CW goat anti-mouse immunoglobulin-G (IgG) secondary antibody (1:5,000, 926-32210), IRDye 680CW goat anti-rabbit IgG secondary antibody (1:5,000, 926-68071) and IRDye 680RD donkey anti-mouse (1:5,000, 926-68072)). For the detection of IDO1 protein levels in BT549 and SKOV-3 cells, the membranes were blocked with 5% milk in PBS + 0.1% Tween-20 (PBS-T), followed by incubation with anti-IDO1 (1:5,000, ab211017, Abcam) and HRP-conjugated secondary antibody (1:5,000, 31460, Pierce Biotechnology). For the detection of DOCK-8 and RHOBTB3 protein levels in HEK293T cells, the membranes were blocked with 5% milk in PBS-T, followed by incubation with anti-DOCK-8 (1:500, 11622-1-AP, Proteintech) or RHOBTB3 (1:800, 13945-1-AP) and HRP-conjugated secondary antibody (1:5,000, 31460, Pierce Biotechnology). KLHDC3 protein levels in U2OS, U2OS KO or HEK293T cells were detected by blocking the membranes with 5% milk in PBS-T and using anti-KLHDC3 antibody (1:1,000, HPA030131, Atlas) and anti-rabbit HRP-conjugated secondary antibody (1:3,000, 7074, Cell Signaling Technology). Primary antibodies were incubated at 4 °C overnight. Washing steps were performed with PBS-T. Chemiluminescence was detected using SuperSignal West Dura Extended Duration Substrate (Thermo Fisher Scientific). Protein bands were visualized using a ChemiDoc MP system (Bio-Rad). Band intensities were quantified using Image Lab software 6.0 (Bio-Rad).

### Reverse transcription-quantitative PCR

BxPC3 cells (100,000 cells well^−1^) or SKOV-3 cells (150,000 cells well^−1^) were seeded in a 24-well plate or 12-well plate, respectively. BxPC3 cells were incubated for 24 h before treatment with the indicated compound concentrations and 50 ng ml^−1^ IFN-γ. SKOV-3 cells were directly treated with or without 10 µM succinyl acetone for 24 h before the addition of hemin for a further 24 h. Total RNA was isolated using an RNeasy Mini Kit as described by the manufacturer (Qiagen). The obtained RNA was reverse-transcribed into complementary DNA (cDNA) using a QuantiTect reverse transcription kit according to the manufacturer’s instructions (Qiagen) before the quantitative polymerase chain reaction (qPCR) using a QuantiFast SYBR green PCR kit (Bio-Rad). Gene-specific primers for *IDO1* (forward: 5′-GCCTGATCTCATAGAGTCTGGC-3′ and reverse: 5′-TGCATCCCAGAACTAGACGTGC-3′)^30^ and *GAPDH* (forward: 5′- GTCTCCTCTGACTTCAACAGCG-3′ and reverse: 5′-ACCACCCTGTTGCTGTAGCCAA-3′) as a reference gene were used. qPCR was performed using a CFX96 real-time PCR detection system (Bio-Rad). Data were analysed using CFX Manager (Bio-Rad). *IDO1* expression levels in each sample were normalized to the levels of the reference gene *GAPDH*. Relative quantification was performed using the 2^−ΔΔCt^ method^63^.

### IDO1 promoter-dependent reporter gene assay

For the assay, the pXPG-IDO1 plasmid containing a firefly luciferase (Fluc) gene under the control of the full-length *IDO1* promoter (kindly provided by G. M. Doody) was used as a reporter. The *Renilla* (Rluc) luciferase expression plasmid pRL-TK vector (Promega Corporation) was used as a control. HEK293T cells were transiently reverse-transfected with the plasmids using Lipofectamine 2000 (Invitrogen) following a modified protocol of the manufacturer’s guidelines. The Fluc plasmid (4 μg) and 300 ng of the Rluc plasmid were mixed in serum-free Opti-MEM in a 1:3 ratio with Lipofectamine 2000. The mixture was added to 9 ml of cell suspension at a cell density of 2.78 × 10^5^ cells ml^−1^, then 25,000 cells were seeded per well in a clear 96-well plate and incubated for 24 h. The next day, *IDO1* promoter-mediated Fluc expression was induced by the addition of 50 ng ml^−1^ IFN-γ with simultaneous compound treatment. The cells were incubated for 48 h before determining the activity of Fluc and Rluc using the Dual-Glo Luciferase assay system with a Spark multimode microplate reader (Tecan). Fluc/Rluc ratios were normalized to the values of the DMSO control (set to 100%).

### CHX pulse assay

BxPC3 cells (200,000 cells well^−1^) were seeded in a 12-well plate, and 50 ng ml^−1^ IFN-γ was added. After 24 h, 5 µM cycloheximide (CHX) was added 30 min before the addition of 5 µM iDeg-1, followed by cell lysis preparation and immunoblotting.

### TUBE pulldown

Tandem ubiquitin binding entity (TUBE) protein was expressed from pGex-6P-1-GST-4xUBA-His (UBA, ubiquitin-associated domains) in *Escherichia coli* and purified by affinity purification using a Ni-NTA column. TUBE protein expression was induced by isopropyl β-D-1-thiogalactopyranoside (IPTG) for 4 h at 30 °C. Afterwards, the cells were lysed in buffer containing 20 mM sodium phosphate (pH 7.4), 500 mM NaCl, 20 mM imidazole, 1 mM PMSF (phenylmethylsulfonylfluoride; 39107.01, Serva) and 1 mM 2-mercaptoethanol, and the TUBE protein was separated using a Ni-NTA affinity chromatography. The TUBE protein was eluted using 20 mM sodium phosphate (pH 7.4) buffer containing 500 mM imidazole and 1 mM 2-mercaptoethanol and subsequently applied to a size exclusion column (HiLoad 16/600 Superdex 200 pg) in 20 mM potassium phosphate buffer (pH 7.4, supplemented with 300 mM NaCl, 1 mM dithioerythritol (DTE) and 2% glycerol) to yield pure TUBE protein.

For the TUBE pulldown, BxPC3 cells were seeded in either 10- or 15-cm^2^ cell culture dishes and after cell attachment were treated with 40 ng ml^−1^ IFN-γ to induce IDO1 expression overnight. Next, the medium was aspired and medium containing the respective concentration of iDeg-1 was added and incubated for 6 h at 37 °C and 5% CO_2_. For proteasome inhibition, 450 nM CFZ (ab216469, abcam) was added 1 h before the addition of iDeg-1. After compound incubation, cell lysates were prepared in TUBE lysis buffer (20 mM Na_2_HPO_4_, 20 mM NaH_2_PO_4_, 1% NP-40 Alternative, 2 mM ethylenediaminetetraacetic acid (EDTA) and 10 mM *N*-ethylmaleimide (NEM), freshly prepared) supplemented with 100 μg ml^−1^ TUBE protein. The samples were kept on ice for 30 min, followed by a centrifugation step for 30 min at 16,000*g* and 4 °C. Protein concentrations were determined to adjust the protein concentration before the addition of 40 µl of GST magnetic beads (Thermo Fisher). The samples were rotated for 2 h at 4 °C, followed by four washing steps using ice-cold PBS-T for 5 min. To elute ubiquitinated protein species, 20 µl of 1× SDS sample buffer was added to the beads and incubated for 15 min at 55 °C. Afterwards, all samples were boiled for 5 min at 98 °C before separation by SDS–PAGE and immunoblotting for IDO1 and vinculin as a control.

### Electroporation of rhIDO1 into HEK293T cells

HEK239T cells (3,000,000 cells per electroporation) were electroporated with 80 µg of rhIDO1 utilizing a NeonTM transfection kit according to the manufacturer’s instructions (Thermo Fisher). For electroporation, 1,000 V (two pulses for 35 ms) were applied using a Neon transfection system pipette station. Afterwards, the cells were washed in pre-warmed PBS followed by resuspension in 3 ml of trypsin/EDTA solution, and were then incubated for 3 min at 37 °C. The electroporated cells were washed again and resuspended in phenol red-free DMEM growth medium (900,000 cells ml^−1^). For the Kyn assay in HEK^rhIDO1^ cells, the cells were plated in 96-well plates and supplemented with 150 µM L-Trp and the respective compound concentrations for 24 h before readout as described above for the manual Kyn assay. For immunoblotting, electroporated cells were seeded in a 12-well plate and treated with the indicated compounds for 6, 14 or 24 h before immunoblotting as described above. For co-treatment, the cells were pre-treated with the proteasome inhibitor CFZ or the neddylation inhibitor MLN4924 for 30 min before iDeg-1 addition.

### HEK293T-based Kyn assay

HEK293T cells (270,000 cells ml^−1^) were transfected with 0.5 µg of pCMV3-IDO1 (Sino Biological US) using Lipofectamine 2000 (Thermo Fisher) while seeding in a 96-well plate, followed by incubation overnight. L-Trp (500 µM) and compounds at the indicated concentrations were then added, and the cells were incubated for 24 h before the detection of Kyn levels using *p*-DMAB as described above for the manual Kyn assay.

### Proteome profiling

The HEK239T cells were electroporated with rhIDO1 as described above and treated with 10 µM iDeg-1 or DMSO for 6 h before lysate preparation by means of four freeze–thaw cycles in PBS containing 0.4% NP-40 Alternative. The protein concentration was determined, and 200 μg of protein lysates were subjected to sample preparation and MS analysis. Protein lysates were dissolved in an equal volume of 100 mM triethylammonium bicarbonate (TEAB) buffer supplemented with 7.5 µl of 200 mM tris(2-carboxyethyl)phosphine (TCEP) and incubated at 55 °C for 1 h. Afterwards, 7.5 µl of 375 mM iodoacetamide were added and the samples incubated for another 30 min at room temperature in the dark before acetone-based protein precipitation (incubation at −20 °C overnight). The next day, the samples were centrifuged at 8,000*g* and 4 °C, the supernatant was aspired, and protein pellets were dried at room temperature. The pellets were dissolved in 50 mM TEAB containing trypsin/LysC (25:1 protein:protease ratio (wt/wt), pre-dissolved in 1 mM HCl (trypsin/LysC mix, MS grade, 5× from Promega Corporation)) and incubated at 37 °C overnight while shaking. The next day, the samples were dried in a vacuum concentrator at 30 °C and subjected to MS.

### Mass spectrometry

All solvents used during MS measurements were LC–MS grade. Digested samples were first dissolved in 120 μl of 20 mM ammonium formate (NH_4_COOH) at pH 11 in an ultrasonicator for 15 min, followed a 15-min incubation step while rotating, and a subsequent centrifugation step (8,000*g* for 3 min at room temperature). Each sample (50 μl) was separated into ten fractions using an XBridge C18 column (130 Å, 3.5 μm, 1 mm × 150 mm) and a U3000 capHPLC system (Thermo Fisher Scientific) under high pH conditions to reduce the sample complexity and increase the number of quantified proteins. Separation was performed at a flow rate of 50 μl min^−1^ starting with 95% solvent A (20 mM NH_4_COO pH 11 in H_2_O)/5% solvent B (40% 20 mM NH_4_COO pH 11, 60% acetonitrile in H_2_O), isocratic for the first 10 min, followed by a linear gradient increasing solvent B up to 25% over 5 min. Afterwards, a second linear gradient was applied, increasing solvent B up to 65% in 60 min, followed by a third gradient increasing solvent B up to 100% over 10 min. Fractions were detected at a wavelength of 214 nm.

The ten fractions were collected at 15 and 100 min (30 s per fraction, circular fractionation using ten vials) and dried in a SpeedVac unit at 30 °C until complete dryness for final analysis by nanoHPLC–MS/MS. The dried samples were then dissolved in 10 μl of 0.1% trifluoroacetic acid (TFA) in water before injection of 9 μl onto an UltiMateTM 3000 RSLCnano system (Thermo Fisher Scientific), coupled online to a Q Exactive HF Hybrid Quadrupole-Orbitrap mass spectrometer equipped with a nanospray source (Nanospray Flex Ion Source, Thermo Fisher Scientific).

To detect the peptides within the samples, a mass range of *m*/*z* 300–1,650 was achieved with a resolution of 60,000 for a full scan, before 15 high energy collision dissociation (HCD) MS/MS scans of the most intense at least doubly charged ions using a resolution of 15,000 and a nominal collision energy (NCE) of 27%. The obtained data were analysed using MaxQuant software (v.1.6.17.0)^64^ including the Andromeda search algorithm and using the human reference proteome of the UniProt database. A full enzymatic trypsin cleavage search was conducted allowing for two miscleavages. Carbamidomethylation was chosen as a fixed modification, and oxidation of methionine and acetylation of the N terminus as variable modifications.

For the first analysis, the mass accuracy for the full mass spectra was set to 20 ppm, and in the second search it was set to 4.5 ppm and for the MS/MS spectra to 20 ppm. A false discovery rate (FDR) of 1% was selected for peptide and protein identification, and only proteins for which at least two peptides were quantified were chosen for further validation. Relative protein quantification was achieved by means of the label-free quantification algorithm implemented in MaxQuant.

To consider the differences in IDO1 concentration after electroporation of IDO1 into the HEK293T cells, all label-free quantification (LFQ) intensities were normalized to those of the DMSO control of the same biological replicate by dividing the LFQ intensity of the sample by that of the corresponding DMSO control (resulting in a value of 1 for the DMSO controls themselves).

Statistical data analysis was carried out using Perseus^65^ v.1.6.14.0. The normalized LFQ intensities were log-transformed (log_2_) and grouped. Proteins were only retained for further analysis when quantified at least twice in at least one of the groups. Missing values were ascribed as ‘0’ and a both-sided *t*-test with error-corrected *P* values (FDR = 0.01, S0 = 0.5) was conducted. Results were exported into an Excel file (xlsx) and visualized using Perseus. The detailed analysis procedure is described in refs. ^64,65^.

### IDO1 immunoprecipitation followed by MS analysis

BxPC3 cells were seeded in 10-cm^2^ dishes and stimulated with 40 ng ml^−1^ IFN-γ overnight at 37 °C and 5% CO_2_. The next day, fresh medium was added containing 20 µM of iDeg-1 or DMSO as a control, and incubated for 8 h before cell detachment and cell lysis using immunoprecipitation lysis buffer (20 mM Tris-HCl pH 7.5, 100 mM NaCl, 0.1 mM EDTA and 0.5% NP-40 Alternative) supplemented with fresh *N*-ethylmaleimide as well as phosphatase (PhosSTOP phosphatase inhibitors, Sigma-Aldrich) and protease inhibitors (cOmplete protease inhibitor cocktail, Sigma-Aldrich). Three freeze–thaw cycles were performed for cell lysis, followed by centrifugation at 14,000*g* for 20 min at 4 °C and determination of the protein concentration (DC protein assay, Bio-Rad). The lysate (800 µg) was subjected to immunoprecipitation.

For the following steps, a magnetic rack was used to separate magnetic beads from the supernatant. First, the cell lysates were pre-cleared using 25 µl of Pierce Protein A/G magnetic beads per sample for 1 h at 4 °C while rotating. The lysates were then added to pre-coated Pierce Protein A/G magnetic beads with IDO1 antibody (ab211017, Abcam), followed by continuous rotation for 2 h at 4 °C. The beads were washed three times with immunoprecipitation wash buffer (50 mM PIPES pH 7.4, 150 mM NaCl, 5 mM MgCl_2,_ 0.5 mM ethyleneglycol-bis-(2 aminoethylether)-N,N.N’,N’-tetraacetic acid (EGTA), 0.1% NP-40 Alternative, 0.1% Triton X-100, 0.1% Tween 20 and 1 mM dithiothreitol (DTT)) for 5 min while rotating. Subsequently, the beads were transferred to a fresh sample tube after being washed three times with 500 µl of PBS, before further washing steps with 500 µl of PBS (thrice) to remove detergents. The beads were resuspended in 50 µl of denaturation buffer (50 mM Tris pH 7.5, 8 M urea and 1 mM DTT) and incubated for 30 min while shaking before the addition of 5.55 μl of 50 mM chloroacetamide solution for another 30 min while shaking. For on-bead digestion, 1 μl of LysC (stock solution of 0.5 μg μl^−1^ in water) was added for 1 h at 37 °C while shaking. The LysC digestion supernatant was transferred into a fresh sample tube, and to the remaining beads, 165 μl of a 50 mM Tris (pH 7.5) solution containing 0.25 μg trypsin was added and incubated for another 1 h at 37 °C while shaking. The two digestion solutions were combined and supplemented with an additional 0.5 μg of trypsin, and the digestion was continued overnight at 37 °C while shaking. The remaining beads were discarded. The next day, the addition of 20 μl of 10% TFA stopped the digestion, and the samples were desalted by stage tip purification. A double layer of C18 chromatography matrix was placed into a 200-µl pipette tip and the matrix was activated by adding 100 μl of 100% methanol. The matrix was washed once with buffer A (0.1% formic acid, 80% acetonitrile in H_2_O) and twice with buffer B (0.1% formic acid in H_2_O). For each washing step, centrifugation in a tip centrifuge was performed. Afterwards, the samples were subjected to tip washing once with buffer B, before sample elution, twice, with 20 μl of buffer A. The samples were dried in a vacuum concentrator at 30 °C, and analysed by MS.

All solvents used during MS measurements were LC–MS grade. The samples were dissolved in 20 μl of 0.1% TFA in water and 3 μl were subjected to nanoHPLC–MS/MS analysis. The samples were injected into an UltiMateTM 3000 RSLCnano system (Thermo Fisher Scientific), coupled online to a Q Exactive Plus Hybrid Quadrupole-Orbitrap mass spectrometer equipped with a nanospray source (Nanospray Flex Ion Source, Thermo Scientific). Samples were desalted by means of a pre-column cartridge (5 μm, 100 Å, 300-μm ID × 5 mm; Dionex) using 0.1% TFA in water and a flow rate of 30 μl min^−1^ for 5 min, with eluent flow to waste followed by back-flushing of the sample during the whole analysis from the pre-column to the PepMap100 RSLC C18 nanoHPLC column (2 μm, 100 Å, 75 μm ID × 50 cm, nanoViper, Dionex). A linear gradient starting with 95% solvent A (0.1% formic acid in H_2_O) and 5% solvent B (0.1% formic acid in acetonitrile) was used, which was increased to 30% solvent B over 90 min (flow rate 300 nl min^−1^). Afterwards, the column was washed (95% solvent B as the highest acetonitrile concentration) and re-equilibrated to the starting conditions. Finally, to detect the peptides within the samples, the nanoHPLC was coupled online to the Quadrupole-Orbitrap mass spectrometer using a standard uncoated SilicaTip (ID 20 μm, Tip-ID 10 μM, New Objective). A mass range of *m*/*z* 300–1,650 was achieved with a resolution of 70,000 for the full scan, before ten HCD MS/MS scans of the most intense at least doubly charged ions using a resolution of 17,500 and an NCE energy of 25%.

The obtained data were analysed using MaxQuant software (v.1.6.17.0)^64^ including the Andromeda search algorithm and using a database containing the IDO1 (UniProt accession no. P14902) or the ubiquitin (UniProt accession no. P0CG48) sequence, together with typical contaminants included in the MaxQuant software. The search was performed for full enzymatic trypsin cleavages allowing two miscleavages. For the protein modifications, carbamidomethylation was chosen as fixed, and oxidation of methionine, acetylation of the N terminus, and GlyGly of lysine as variable modifications. The mass accuracy for the full mass spectra was set to 20 ppm (first search) and 4.5 ppm (second search), respectively, and for the MS/MS spectra to 20 ppm. The FDRs for peptide and protein identification were set to 1%. Results were exported into a txt file and visualized using Prism 6.0 (GraphPad). Details of the analysis procedure are described in ref. ^64^.

### Cellular thermal shift assay in intact cells (InCell CETSA)

SKOV-3 cells were seeded into a T-75 flask (or a six-well plate for the isothermal dose–response experiment) 48 h before compound addition. On day 3, the indicated iDeg-1 concentrations or DMSO as a control were added for 1 h and incubated at 37 °C and 5% CO_2_ before cell detachment using trypsin. The cells were washed twice with PBS (4 °C, 300*g*), then the cell pellet was resuspended in PBS and equally divided into ten different PCR sample tubes that were subjected to gradient heat treatment (3 min) using a Mastercycler EP gradient system (37, 40.8, 44.1, 47.4, 50.0, 53.4, 55.7, 56.3, 61.0 and 63.7 °C).

For the isothermal dose–response analysis of iDeg-1, the cells were heat-treated at a constant temperature of 50 °C. Afterwards, the SKOV-3 cells were placed on ice and cell lysis was performed by adding 0.4% NP-40 before freeze–thaw lysis. The samples were then centrifuged at 100,000*g* at 4 °C for 20 min to separate denaturated protein. The supernatants were collected and subjected to SDS–PAGE and immunoblotting, followed by immunostaining for IDO1. The obtained band intensities were quantified using Image Lab software and normalized to the intensities of the bands at 37 °C. Melting temperatures in the presence or absence of the test compound were determined via nonlinear regression using Prism 6.0 (GraphPad).

### Protein translation assay in cells

BxPC3 cells (100,000 cells well^−1^) were seeded in a 96-well plate and incubated at 37 °C and 5% CO_2_ overnight before the addition of 25 ng ml^−1^ IFN-γ and the respective compounds for 24 h. Afterwards, the cells were washed three times with pre-warmed PBS followed by protein translation analysis using a Click-iT HPG Alexa Fluor 488 protein synthesis assay kit (Thermo Fisher). Fresh medium containing the respective compounds as well as 50 µM L-homopropargylglycine (L-HPG) to tag newly synthesized proteins were added to the cells, followed by incubation for 45 min at 37 °C and 5% CO_2_. The cells were then fixed using 4% *para*-formaldehyde, and the L-HPG was fluorescently labelled with Alexa 488 reagent provided by the assay kit using a click reaction as described by the manufacturer (Click-iT HPG Alexa Fluor 488 protein synthesis assay, Thermo Fisher). Cell nuclei were stained using the nuclei stain provided in the assay kit. Images were acquired at ×10 magnification using an Axiovert 200M microscope (Zeiss). The images were analysed using MetaXpress software. The L-HPG-mediated fluorescence intensity as well as a cell count analysis were performed, and the obtained data were normalized to the cell count and visualized by Fiji ImageJ. Data were plotted using Prism 6.0 (GraphPad).

### In vitro IDO1 translation assay

Full-length IDO1 cDNA was cloned into the pT7CFE vector (pT7CFE1-IDO1) and used in the in vitro translation assay with a 1-Step Human Coupled DNA IVT kit (Thermo Fisher). To obtain the proteins required for protein translation, HeLa cell lysate was prepared according to the manufacturer’s instructions. The obtained lysates were combined with pT7CFE1-IDO1 plasmid and supplemented with the reagents provided with the 1-Step Human Coupled DNA IVT kit (Thermo Fisher). The reaction mixture was incubated for 3 h at 30 °C followed by immunoblotting for IDO1.

### Cloning and purification of recombinant IDO1 protein and KLHDC3-EloB/C complex

Full-length human IDO1 cDNA was cloned into the pGEX6p-2rbs vector using EcoRI and SalI restriction sites. For protein translation assays, full-length IDO1 cDNA was cloned into the pT7CFE vector (pT7CFE1-IDO1). Human recombinant IDO1 protein was expressed in *E. coli* according to ref. ^66^, yielding pure and active IDO1 protein. IDO1 protein expression in *E. coli* was induced by the addition of 300 µM IPTG, and the cells were incubated at 20 °C overnight. Harvested cells were resuspended in lysis buffer (50 mM Tris-HCl pH 7.4, 100 mM NaCl and 1 mM PMSF, DNase), disrupted by sonication and the lysate was centrifuged at 13,000*g* for 35 min at 10 °C. The supernatant was applied to a GST Trap HP column to enrich GST-tagged IDO1 equilibrated in 50 mM Tris-HCl, 150 mM NaCl and 1 mM DTE, pH 7.0. To obtain untagged full-length IDO1, GST was cleaved from IDO1 overnight using PreScission protease. Eluted IDO1 protein was further purified using a size exclusion column (26/60 G75 HiLoad) in 50 mM Tris-HCl pH 7.4, 100 mM NaCl to yield pure rhIDO1.

For hIDO1crystallization, a truncated version (5–400) of IDO1 was cloned into the pGEX6p-2rbs vector using the EcoRI and SalI restriction sites. Haem was removed by overnight incubation with 166 mM 2-mercaptoethanesulfonate (MESNA) to obtain apo-IDO1. Haem occupancy was determined by UV–vis spectrophotometry.

Expression constructs for KLHDC3-EloB/C (both monomeric and tetrameric versions) are described in ref. ^55^. Briefly, KLHDC3, EloB and His-TEV-EloC were first cloned into pLib via Gibson assembly. Cassettes were then generated via PCR as described in ref. ^67^ and Gibson-assembled into pBig1a to generate a single vector for co-expression of all components. Proper assembly into pBig1a was confirmed by PmeI and SwaI restriction digestion.

For the ubiquitination assays, IDO1 (residues 5-C) was cloned into a pRSF Duet based vector with an N-terminal His-TEV tag and purified as described above.

KLHDC3-EloB/C was expressed in Hi5 insect cells with an N-terminal 6XHis tag on EloC as described in ref. ^55^. Tagged proteins were purified from cell lysates by Ni affinity chromatography. Nickel elutions were directly loaded onto a HiTrap Q-HP column and eluted with a gradient of 2–50% NaCl. Protein-containing fractions were pooled, concentrated and further purified by size exclusion chromatography in 25 mM HEPES, 200 mM NaCl and 1 mM DTT pH 7.5.

### Protein modifications

To introduce a cysteine for fluorescent labelling of UB and K0UB, we mutated the protein kinase A site in the pGEX2TK backbone converting the PKA site from RRASV to RRACV^68^. UB or UBK0 purified from this expression construct was labelled with AlexaFluor-647-maleimide or fluorescein-5-maleimide, respectively, as described in ref. ^68^. Briefly, DTT was added to UB or UBK0 at a final concentration of 10 mM and incubated on ice for 20 min to completely reduce the cysteines for labelling. DTT was removed by buffer exchange over an NAP-5 column (GE Healthcare) in labelling buffer (25 mM HEPES, 200 mM NaCl). Labelling reactions consisted of UB or UBK0 at 150 µM final concentration and were initiated by the addition of 600 µM AlexaFluor-647-maleimide or fluorescein-5-maleimide (4× excess over labelling target and <5% final DMSO concentration). Reactions were incubated at room temperature for 2 h and quenched by the addition of DTT to 10 mM. Quenched reactions were desalted over a PD-10 column in labelling buffer containing 1 mM DTT to remove unreacted probe. Desalted protein was concentrated and further purified over a Sephadex SD75 column.

### IDO1 enzymatic assay

Full-length rhIDO protein (1 µM) in 50 mM potassium phosphate buffer (50 mM KH_2_PO_4_ and 50 mM K_2_HPO_4_, pH 6.5) was incubated with the respective compounds for 90 min at 37 °C before the addition of 10 mM ascorbic acid, 10 µM methylene blue, 2 mM Trp and 100 µg ml^−1^ catalase (Sigma-Aldrich, C100). The samples were incubated for 60 min, followed by the addition of TCA to a final concentration of 7% and further incubation for 30 min at 37 °C. Kyn levels were detected by the addition of an equal volume of 2% (wt/vol) *p*-DMAB in acetic acid, and absorbance was measured at 492 nm and 650 nm as a background control. Values are presented relative to the DMSO control. Dose–response curves and IC_50_ values were generated and fitted with GraphPad Prism 9.2.0 (GraphPad software) using a four-parameter, variable-slope nonlinear regression curve fit.

### nanoDSF

Full-length rhIDO1 (10 µM) in 100 mM potassium phosphate buffer (pH 6.5) was incubated with the respective compound or DMSO as a control at 37 °C for 3 h, followed by a centrifugation step for 10 min at 1,000*g*. Afterwards, the protein solution was loaded onto a capillary, followed by the measurement of fluorescence intensities at 350 nm and 330 nm from 20 °C up to 90 °C using a Prometheus Panta device (NanoTemper Technologies). Melting curves were visualized by GraphPad Prism 9.2.0 (GraphPad software).

### UV–vis analysis

Full-length rhIDO1 (10 μM) in 100 mM potassium phosphate buffer (pH 6.5) was incubated with the respective compounds or DMSO as a control for 3 h at 37 °C followed by a centrifugation step for 10 min at 1,000*g*. Subsequently, UV–vis spectra of the samples were monitored between 250 and 650 nm in 1-nm increments using UV-transparent microplates (UV-STAR, Greiner) and a Spark multimode microplate reader (Tecan).

### ITC

All experiments were performed on a MicroCal AutoITC200 set-up. KLHDC3-EloB/C was buffer-exchanged by desalting over an NAP-5 column in ITC buffer (25 mM HEPES, 150 mM NaCl, 1 mM 2-mercaptoethanol, pH 7.5). Lyophilized peptides were dissolved in ITC buffer to a final concentration of 1 mM. The titrations used KLHDC3-EloB/C in the sample cell at 24 µM, and 240 µM test peptide in the syringe. Titrations were performed at 25 °C with one injection of 0.4 µl, followed by 12 injections of 3 µl. Experiments were performed *n* = 2 times, and data were evaluated with MicroCal PEAQ software.

To evaluate the binding affinity of iDegs to IDO1, IDO1 protein was dissolved in ITC buffer (50 mM KH_2_PO_4_, 50 mM K_2_HPO_4_ and 1 mM TCEP, pH 6.57) to a final concentration of 125 µM in the syringe, while the sample cell contained iDeg-6 at 50 µM. Titrations were performed at 25 °C with one injection of 0.4 µl, followed by 18 injections of 2 µl. Experiments were performed *n* = 3 times in a MicroCal PEAQ-ITC system (Malvern), and the data were evaluated with MicroCal PEAQ software.

### Co-crystallization of Apo-IDO1 protein with iDeg-1 and iDeg-2

Truncated apo-IDO1 protein (residues 5–400; 10 mg ml^−1^, 225 µM) was incubated with 660 µM iDeg-1 (1.3% vol/vol DMSO) or 450 µM iDeg-2 (1.8% vol/vol DMSO) in binding buffer (25 mM Tris pH 8.0, 100 µM TCEP) at 37 °C for 2 h. The soluble protein fraction was separated from the precipitate by centrifugation at 20,000*g* for 15 min at 20 °C. Crystallization drops were prepared by mixing 0.4 µl of the rhIDO1:iDeg solution with 0.2 µl of precipitant solution in a sitting drop set-up (iQ plates, SPT Labtech) at 20 °C. Crystals were obtained in wells with precipitant solution containing 41% vol/vol PEG200 in 50 mM MES pH 6.66 (iDeg-1) and 39% (vol/vol) PEG200 in 100 mM MES pH 6.95 (iDeg-2). The crystals appeared in one day as thin bars. After five days, the crystals were collected and flash-frozen in liquid nitrogen using the precipitant solution as cryoprotectant. X-ray diffraction data were collected at the Swiss Light Source (SLS, Villigen, Switzerland) on beamline X10SA (iDeg-1) and at the European Synchrotron Radiation Facility (ESRF, Grenoble, France) on beamline ID30B (iDeg-2). Data were processed with XDS and scaled using XSCALE. The iDeg-2 structure was solved by molecular replacement with Phaser^69^ from the Phenix suite^70^ using as initial model the coordinates of the IDO1-apoxidole structure (PDB 8ABX). The iDeg-1 structure was solved in the same manner using the determined IDO1-iDeg-2 structure (PDB 9FOH) as the initial model. Iterative refinement cycles were carried out using phenix.refine and COOT^71^. Figures were generated using the PyMOL Molecular Graphics system (version 2.5.4, Schrödinger). PDB ID codes, data collection and refinement statistics are presented in Supplementary Table 2.

### Ubiquitination assays

The use of pulse-chase assays allowed comparison of the paths of UB transfer starting from UBE2R2 or UBE2D2. First, UBE2R2 or UBE2D2 was pulse-labelled by incubating a mixture of UBA1 (0.3 µM), E2 (10 µM) and fluorescently labelled UB or KOUB (15 µM) in 25 mM HEPES, 100 mM NaCl, 100 mM MgCl_2_, 2 mM ATP, pH 7.5 at room temperature for 13 min. Pulse-loading reactions were quenched by the addition of EDTA to 50 mM and incubated on ice for 5 min. Ubiquitination chase reactions consisted of mixing the E2~*UB thioester conjugate (0.4 µM final concentration) with the indicated pre-equilibrated NEDD8~CUL2-RBX1-KLHDC3-EloB/C (0.3 µM final concentration) with a threefold excess of IDO1 variants in 25 mM HEPES, 100 mM NaCl, 50 mM EDTA, 0.5 mg ml^−1^ bovine serum albumin, pH 7.5 at room temperature. Experiments following loading of apo-IDO1 with small molecules consisted of incubating IDO1 (15 µM final concentration) with a twofold excess of the indicated test molecules for 1.5 h at 37 °C. Mixtures were pelleted for 10 min at 14,000 r.p.m. to remove insoluble material. A UV–vis spectrum of the resulting supernatant confirmed haem incorporation, and the samples were directly diluted into ubiquitination reactions. Reactions were quenched at the indicated times with 2× SDS–PAGE sample buffer and separated on 4–12% Bis-Tris gradient gels and scanned for fluorescence on a Typhoon imager. All experiments, except where indicated, utilized a monomeric version of KLHDC3-EloB/C.

To assess the relative binding strength of apo-IDO1, or small-molecule-bound variants of IDO1, we employed a competition pulse-chase ubiquitination assay. First, KLHDC3-EloB/C (50 nM) was equilibrated at room temperature for 15 min with 200 nM IDO1^C-deg^ peptide and varied concentrations of full-length apo-IDO1, iDeg-6-bound IDO1 or linrodostat-bound IDO1. Chase reactions were then initiated by the addition of the pre-formed fluorescent UBE2R2~UB thiolester conjugate (100 nM final concentration). Reaction aliquots were removed at 30 s and quenched with 2× SDS–PAGE sample buffer, separated on 4–12% Bis-Tris gradient gels, and scanned for fluorescence on a Typhoon imager. Data were quantified and visualized as the loss of UB~IDO1^C-deg^ ubiquitination upon increasing concentrations of full-length IDO1 variants, yielding apparent IC_50_ values for KLHDC3 binding by the differing IDO1 full-length variants.

Biochemical assays testing the order of addition of small molecules as shown in Fig. 5m, were performed by first incubating apo-IDO1 (14 mM) with the indicated small molecules (42 µM) for 20 min at room temperature. A second test molecule was then added (42 µM) and incubated for an additional 20 min at room temperature. The mixtures were then diluted to a final concentration of 500 nM of IDO1 containing NEDD8~CUL2^KLHDC3^ (200 nM final concentration). Ubiquitination chase reactions were initiated by the addition of UBE2R2~UB^R7^ thioester conjugate (300 nM). Reactions were quenched at the indicated times with 2× SDS–PAGE sample buffer and separated on 4–12% Bis-Tris gradient gels and scanned for fluorescence on a Typhoon imager.

### Reporter and sgRNA generation

The N-terminal stability reporter was generated starting from the previously reported C-terminal stability vector^49,72^. The backbone was digested using SalI and MluI (NEB), and the mCherry and TagBFP inserts were amplified using Phusion High-Fidelity DNA polymerase following the manufacturer’s instructions and using the primers indicated in Supplementary Table 3. Fragments were subsequently assembled using an NEBuilder HiFi DNA Assembly cloning kit. The MluI restriction site was maintained and utilized for insertion of the various IDO1 constructs amplified with the primers indicated in Supplementary Table 3 and again assembled using the NEBuilder HiFi kit. As starting material for the genetic versions of IDO1, Addgene plasmid 187026 (pcDNA3.1-IDO1-p2a-eGFP gifted by G. van den Bogaart^73^) was used. The custom-made sgRNA library targeting elements of the ubiquitin proteasome system^51^ was generated as previously described in ref. ^49^. Single sgRNAs were designed using the VBC score^74^ and ordered as custom oligos (Sigma). After phosphorylation (T4 PNK, NEB) and annealing of the oligos, an NEBridge Golden Gate assembly kit (BsmBI-v2) was used for insertion of the respective sgRNAs (sequences are provided in Supplementary Table 4). sgRNAs were cloned into pLenti-U6-sgRNA-IT-EF1αs-THY1.1-P2A-NeoR^49^. Insertion of the correct DNA sequence was verified by Sanger sequencing (Microsynth).

For IDO1 F270, R343A, H346A and T395M, pENTR223 IDO1 was mutagenized using Q5 site-directed mutagenesis (NEB), followed by Gibson assembly to yield the respective N-terminal stability reporters. The used oligunucleotides were as follows:

**Table:** 

F270A_fw	gcgCAGTGCTTCGACGTGC
F270A_rev	CACGCTGCTCTGGCC
R343A_fw	gcgAGCTACCACCTGCAGATC
R343A_rev	CAGGCTCACCAGGGC
T395A_fw	GCGgagaagagcctgctgaaggag
T395A_rev	ggtgctgcgcacggtc
T395M_fw	ATGgagaagagcctgctgaaggag
T395M_rev	ggtgctgcgcacggtc
H346A_fw	gcgCTGCAGATCGTGACCAAG
H346A_rev	GTAGCTGCGCAGGCTC

### Virus production, transductions and cell line generation for the CRISPR–Cas9-based screen

Lenti-X cells were transfected at 70% confluence with the reporter or sgRNA plasmids as well as the two packaging plasmids (pCMVR8.74 helper and pMD2.G envelope) using polyethylenimine (PEI MAX *M*_w_ 40,000, Polysciences). pCMVR8.74 was a gift from D. Trono (Addgene plasmid 22036). pMD2.G was a gift from D. Trono (Addgene 12259). The virus was collected and filtered using a 0.45-μm poly-ethersulfone filter. One million KBM7 iCas9 cells or KBM7 iCas9 IDO1 N-terminal stability reporter cells per 2 ml were transduced with varying volumes of virus solution and incubated with 8 µg ml^−1^ polybrene for 24 h before cell expansion. Cells were selected either via FACS using a CytoFLEX SRT Benchtop Cell Sorter (for reporter cell lines, BFP and mCherry positive cells, Extended Data Fig. 7c) or using G418 (1 mg ml^−1^, Gibco) for sgRNA-harbouring cell lines, which was added 72 h after transduction. KO cell lines were generated by selection of the respective sgRNA (sgAAVS1 = CTRL or sgRNA1/2 KLHDC3 for KO1/KO2) for at least 14 days with G418 followed by subsequent induction with doxycycline (0.4 μg ml^−1^, PanReac AppliChem) and cell recovery.

### Flow-cytometric IDO1 reporter assay

IDO1 KBM7 reporter cells were treated for the times, compounds and concentrations indicated in the respective figure legends or as specified in the text. All compounds were diluted starting from DMSO stock solutions. Blue fluorescent protein (BFP) and mCherry levels were measured using an LSR Fortessa (BD Biosciences) with BD FACSDiva software (v9.0). To quantify the changes across conditions, the mean BFP and mCherry values were exported after gating for singlets and reporter positive cells (for the gating strategy see Supplementary Fig. 1a,c) utilizing FlowJo 10.6.2. Normalization was performed either against the respective *wt IDO1* reporter cells treated with DMSO (Fig. 5h), their respective co-treatment (Fig. 4f and Extended Data Fig. 6d) or for each respective genotype (Figs. 4b,i, 5i and 6f,i and Extended Data Fig. 6e).

### FACS-based CRISPR–Cas9 screen

The screen, library preparation and sequencing analysis were performed as described in ref. ^49^. In brief, 120 million KBM7 iCas9 IDO1 N-terminal stability reporter cells were transduced at a multiplicity of infection (MOI) of ~0.12 sgRNA-positive cells (>1,000-fold library representation, Extended Data Fig. 7e) with the customized ubiquitin focused library. Transduced cells were selected with G418 (1 mg ml^−1^, Gibco) for 14 days, expanded, and Cas9 expression was induced with doxycycline (0.4 μg ml^−1^, PanReac AppliChem). Three days after Cas9 induction, 50 million cells per condition and replicate were treated with DMSO, iDeg-1 or iDeg-2. The experiment was performed in two biological replicates. Cells were washed with PBS, stained with allophycocyanin (APC)-conjugated anti-mouse Thy1.1 antibody (1:400, 202526, BioLegend) and human TruStain FcX Fc receptor blocking solution (1:1,000, 422302, BioLegend) for 5 min at 4 °C. Subsequently, the cells were fixed with BD fixation buffer 4% (Thermo Scientific Pierce) for 45 min at 4 °C. All steps were performed protected from light. Cells were washed twice with PBS and stored in FACS buffer (PBS, 5% FBS and 1 mM EDTA).

For sorting, cells were strained using a 35-μm nylon mesh and sorted on a BD FACSAria Fusion instrument (70-µm nozzle, BD Biosciences) with BD FACSDiva software (v8.0.2). Singlets, Cas9 and sgRNA-positive cells were selected following the scheme shown in Extended Data Fig. 7a–d. Next, fractions with different levels of BFP were enriched: the 5% highest or lowest BFP expressing cells were used for the respective HIGH and LOW fractions, and 30% of the population’s centre was selected for the MID fractions. For each replicate, cells corresponding to at least a 1,000-fold library representation were sorted.

To quantify the sgRNA levels per each fraction and sample, next-generation sequencing (NGS) libraries were prepared. Genomic DNA was isolated by cell lysis (10 mM Tris-HCl, 150 mM NaCl, 10 mM EDTA, 0.1% SDS) and proteinase K treatment (New England Biolabs) overnight at 55 °C, with shaking at 1,200 r.p.m., followed by 2 h of DNAse-free RNAse (Thermo Fisher Scientific) incubation at 37 °C. Next, DNA was extracted by two rounds of phenol extraction and subsequent isopropanol precipitation. Barcoded NGS libraries for each sorted population were generated using a two-step PCR protocol using AmpliTaq Gold polymerase (Invitrogen). The resulting PCR products were purified after each step using Mag-Bind TotalPure NGS beads (Omega Biotek). The final NGS libraries were pooled and sequenced on a NovaSeq 6000 platform (Illumina).

Sequencing reads were trimmed using fastx-toolkit (v0.0.14), aligned using Bowtie2 (v2.4.5), and quantified using featureCounts (v2.0.1). The utilized workflows are available at https://github.com/ZuberLab/crispr-process-nf/tree/566f6d46bbcc2a3f49f51bbc96b9820f408ec4a3 and https://github.com/ZuberLab/crispr-mageck-nf/tree/c75a90f670698bfa78bfd8be-786d6e5d6d4fc455. To calculate gene-level enrichment, the sorted populations (HIGH or LOW) were compared to the MID populations using the median-normalized read counts.

### TurboID -based proximity labelling and streptavidin pulldown

To generate the TurboID-IDO1 construct, TurboID was N-terminally fused to IDO1 via a flexible 5xGGGS linker. The IDO1 coding sequence was PCR-amplified from pCMV3-IDO1 (Sino Biological US) using the following primer pair:

5′ cggcggagggagtggaggaggcagcATGGCACACGCTATGGAAAACTCC-3′ and 5′- ggatctacgtaatacgactcactatagTTAACCTTCCTTCAAAAGGGATTTCTCAGTTG-3′

The pCD-betaG-Flag-TurboID vector (Addgene 124646) was amplified using the following primer pair:

5′-CTATAGTGAGTCGTATTACGTAGATCCAGACATGATAAGATAC-3′ and 5′-ccgccaccgctcccgcctccCTTTTCGGCAGACCGCAGACTGATTTC-3′

The amplified PCR fragments were assembled together with the primer 5′-GGAGGCGGGAGCGGTGGCGGctctggtgggggaagCGGCGGAGGGAGTGGAGGAGGCAGC-3′ to incorporate the 5xGGGS linker using an NEBuilder HiFi DNA assembly cloning kit.

Ten million HEK293T cells were transiently transfected with 4.2 µg of Flag-TurboID-IDO1 using Lipofectamine 2000 (Thermo Fisher) according to the manufacturer’s instructions. At 48 h after transfection, the cells were treated with CFZ for 1 h, followed by treatment with either DMSO or 5 µM iDeg-3 for 2.5 h. During the final 15 min of the incubation, 500 μM biotin (Sigma-Aldrich) was added to the cells to induce biotinylation of proximal proteins. The cells were subsequently washed four times with cold PBS, pelleted at 300*g* for 5 min and lysed in urea lysis buffer (4 M urea, 50 mM Tris, 150 mM NaCl, 1% NP-40 Alternative, 2 mM EDTA, 5% glycerol, 20 mM *N*-ethylmaleimide, protease inhibitor cocktail, pH 7.5) for 1 h on ice, followed by sonication. Lysates were cleared by centrifugation at 17,000*g* for 15 min at 4 °C), then 500 µg of protein from each condition in 500 µl of lysis buffer was incubated with 60 µl of pre-washed streptavidin magnetic beads (Pierce) (washing buffer 1: 10 mM Tris, 150 mM NaCl, 0.1% Tween-20, 1 mM EDTA, pH 7.5) overnight at 4 °C with gentle rotation. The next day, the beads were washed three times with 1 ml of 4 M urea washing buffer 2 (4 M urea, 10 mM Tris, 150 mM NaCl, 0.1% Tween-20, 1 mM EDTA, pH 7.5) followed by a final wash with PBS. Bound proteins were eluted by resuspending the beads in 20 µl of elution buffer (25 mM biotin, 10 mM Tris, 150 mM NaCl, 1 mM EDTA, pH 7.5) and heating at 95 °C for 5 min. This elution procedure was repeated using 10 µl of 2× LDS buffer at 95 °C, and both eluates were combined. The eluates were then dried in a SpeedVac for 2 h, redissolved in 10 µl of Milli-Q water, and the complete eluate was applied to SDS gel. Input and eluted proteins were analysed by immunoblotting against IDO1 and KLHDC3.

### Scratch wound healing assay

The scratch wound healing assay was performed as described in ref. ^34^. SKOV-3 cells were seeded at 0.2 × 10^6^ cells ml^−1^ in 2 ml in six-well plates and cultured overnight to reach a confluence of over 80%. After 24 h of pre-conditioning with epacadostat, iDeg-6 or vehicle, a 10-µl sterile pipette tip was used to make a scratch line on the monolayer of confluent cells at the bottom of the well. The cells were washed twice to remove cellular debris, re-exposed to stimuli or vehicle, and then incubated at 37 °C in a humidified 5% CO_2_ incubator for 48 h. Over this incubation time, the wound healing was continuously observed using an EVOS M5000 imaging system (Thermo Fisher Scientific) with ×10 magnification. Pictures were acquired at different time points. The area of the wound healing was determined using the MRI wound healing tool (ImageJ software, NIH) and the data are reported as percentage of wound closure in relation to time 0.

### Reporting Summary

Further information on research design is available in the Nature Portfolio Reporting Summary linked to this Article.

## Online content

Any methods, additional references, Nature Portfolio reporting summaries, source data, extended data, supplementary information, acknowledgements, peer review information; details of author contributions and competing interests; and statements of data and code availability are available at 10.1038/s41557-025-02021-5.

## Supplementary information

Supplementary Information Supplementary Figs. 1–4, Tables 1–4, Methods and NMR spectra.

Reporting Summary

Supplementary Data 1 Measurement of KLHDC3 binding to C-terminal IDO1 peptides using ITC.

Supplementary Data 2 Measurement of IDO1 binding to iDeg-6 using ITC.

## Source data

Source Data Fig. 1 Statistical source data.

Source Data Fig. 1 Unprocessed western blots.

Source Data Fig. 2 Statistical source data.

Source Data Fig. 2 Unprocessed western blots.

Source Data Fig. 4 Statistical source data.

Source Data Fig. 4 Unprocessed western blots.

Source Data Fig. 5 Statistical source data.

Source Data Fig. 5 Unprocessed western blots.

Source Data Fig. 6 Statistical source data.

Source Data Fig. 6 Unprocessed western blots.

Source Data Extended Data Fig. 1 Statistical source data.

Source Data Extended Data Fig. 1 Unprocessed western blots.

Source Data Extended Data Fig. 2 Statistical source data.

Source Data Extended Data Fig. 2 Unprocessed western blots.

Source Data Extended Data Fig. 3 Statistical source data.

Source Data Extended Data Fig. 3 Unprocessed western blots.

Source Data Extended Data Fig. 4 Statistical source data.

Source Data Extended Data Fig. 4 Unprocessed western blots.

Source Data Extended Data Fig. 6 Statistical source data.

Source Data Extended Data Fig. 7 Statistical source data.

Source Data Extended Data Fig. 7 Unprocessed western blots.

Source Data Extended Data Fig. 8 Statistical source data.

Source Data Extended Data Fig. 8 Unprocessed western blots.

Source Data Extended Data Fig. 9 Statistical source data.

Source Data Extended Data Fig. 9 Unprocessed western blots.

Source Data Extended Data Fig. 10 Statistical source data.

## Data Availability

Data that support the findings of this study have been deposited in MassIVE under accession codes MSV000094270, PXD050474 (global proteome profiling) and MSV000094271, PXD050475 (IDO1 immunoprecipitation). The crystal structures of IDO1 with iDeg-1 and iDeg-2 have been deposited in the PBD under accession nos. 9RIS and 9FOH. Source data are provided with this paper.
