# Supplementary material for: Monovalent pseudo-natural products supercharge degradation of IDO1 by its native E3 KLHDC3
Source: Nat Chem. 2026 Jan 7;18(3):585–96. doi: 10.1038/s41557-025-02021-5 (PMC12962974; doi:10.1038/s41557-025-02021-5)
Supplement: Supplementary file 1 — Supplementary Figs. 1–4, Tables 1–4, Methods and NMR spectra. [file 41557_2025_2021_MOESM1_ESM.pdf]

nature chemistry

Supplementary information

https://doi.org/10.1038/s41557-025-02021-5

Monovalent pseudo-natural products supercharge degradation of IDO1 by its native E3 KLHDC3

In the format provided by the authors and unedited

Table of contents

Supplementary Figures 2
Supplementary Tables 7
Chemistry 11
NMR-spectra 25
References 35

Supplementary Figures

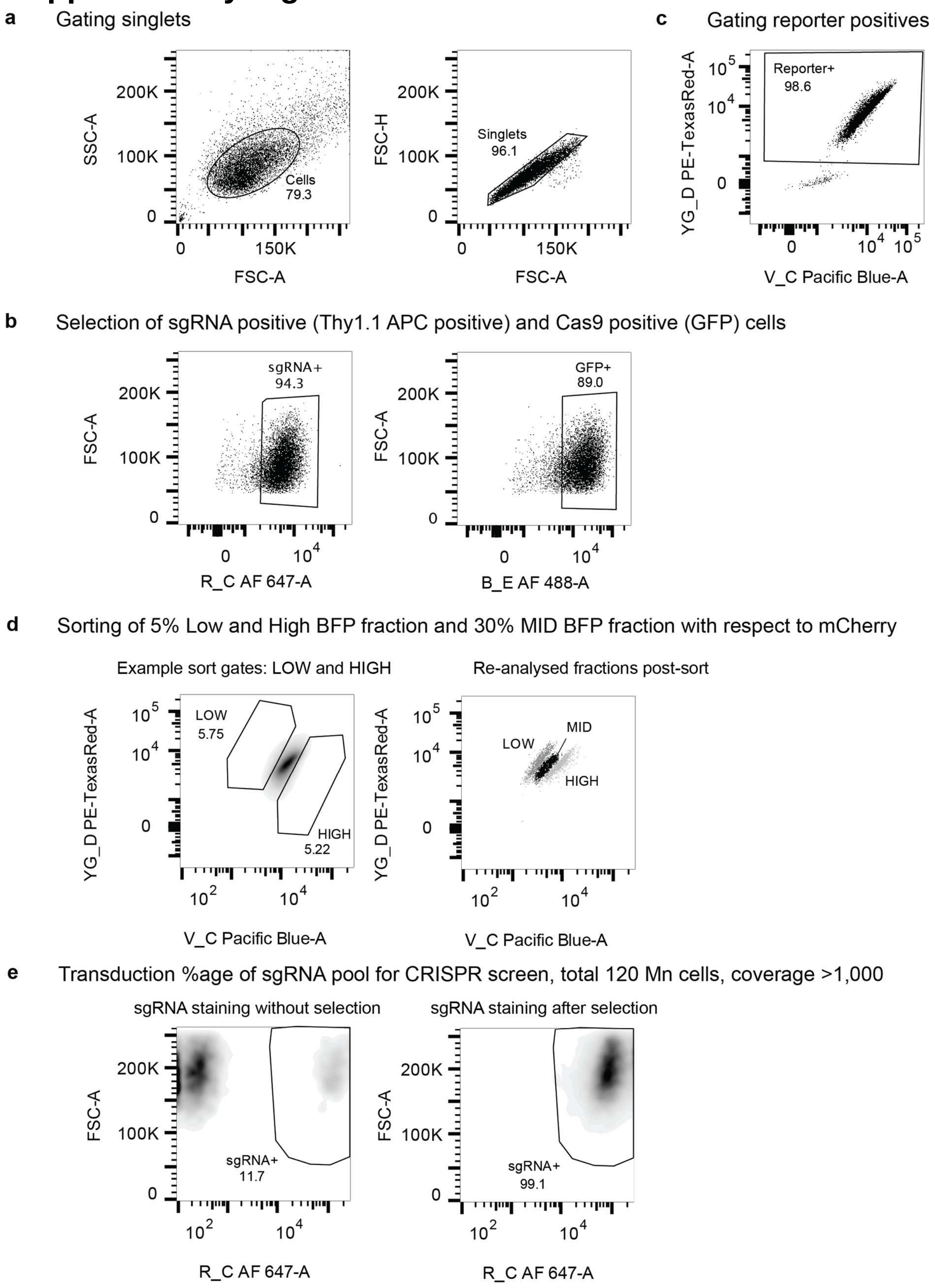

Supplementary Fig. 1: Gating strategy for flow cytometric assays and FACS. a, The identification of viable cells was performed using the forward and side scatter area (FSC-A and SSC-A, respectively).

Singlets were selected using the FSC-A and FSC-H (height) as indicated. **b,** sgRNA transduction was assessed by staining for the Thy1.1 surface antigen for both single and pooled sgRNA experiments. GFP expression monitored Cas9 induction. **c,** Reporter-positive cells were selected in the BFP and mCherry channel. **d,** For the CRISPR-Cas9 screen the highest (HIGH) and lowest 5% (LOW) of BFP expressing cells were sorted and compared against the 30 % mid-fraction (MID). Re-analysis of sorted fractions was routinely performed to confirm sorting accuracy. **e,** The sgRNA pool for the CRISPR-Cas9 screen was transduced at 11.7 % for a total of 120 million cells and selected with G-418 (see Materials and Methods) prior to screening.

a

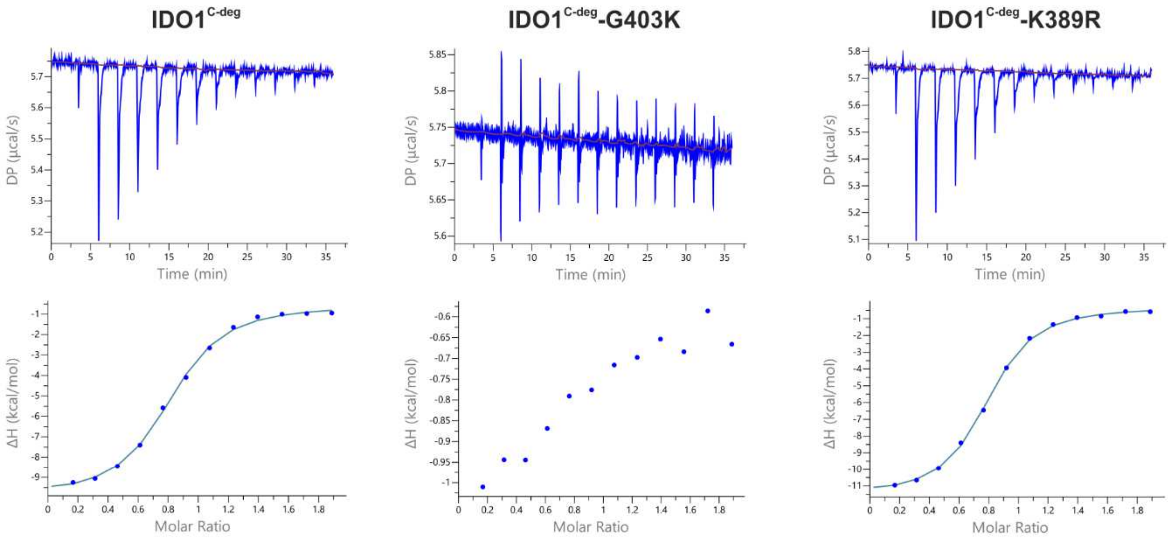

b

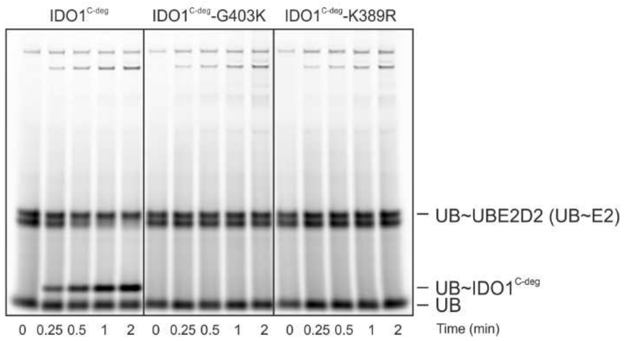

Supplementary Fig. 2: Binding and ubiquitination of C-terminal IDO1 peptides. a, Measurement of KLHDC3 binding to C-terminal IDO1 peptides using ITC. **b,** Fluorescent scan of pulse-chase assay monitoring ubiquitination of C-terminal IDO1 peptides by UBE2D2. UBE2D2 was "pulse" loaded with fluorescent ubiquitin, and the resulting UBE2D2~UB thioester was added to NEDD8~CRL2^{KLHDC3} incubated with the indicated peptides. Samples were quenched with SDS sample buffer at the indicated timepoints. UB: ubiquitin.

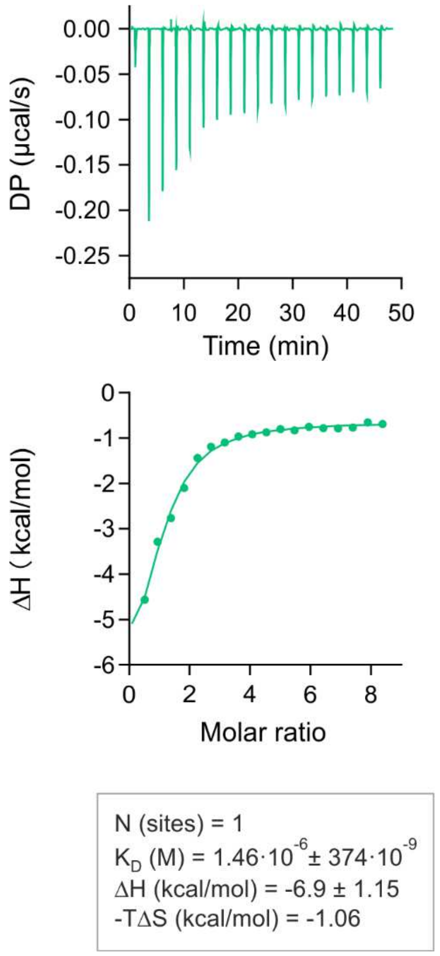

Supplementary Fig. 3: Binding of iDeg-6 to IDO1. Measurement of IDO1 binding to iDeg-6 using ITC. Representative data for n =3 independent experiments. K_D (n =3) = 2.09 ± 0.77 µM.

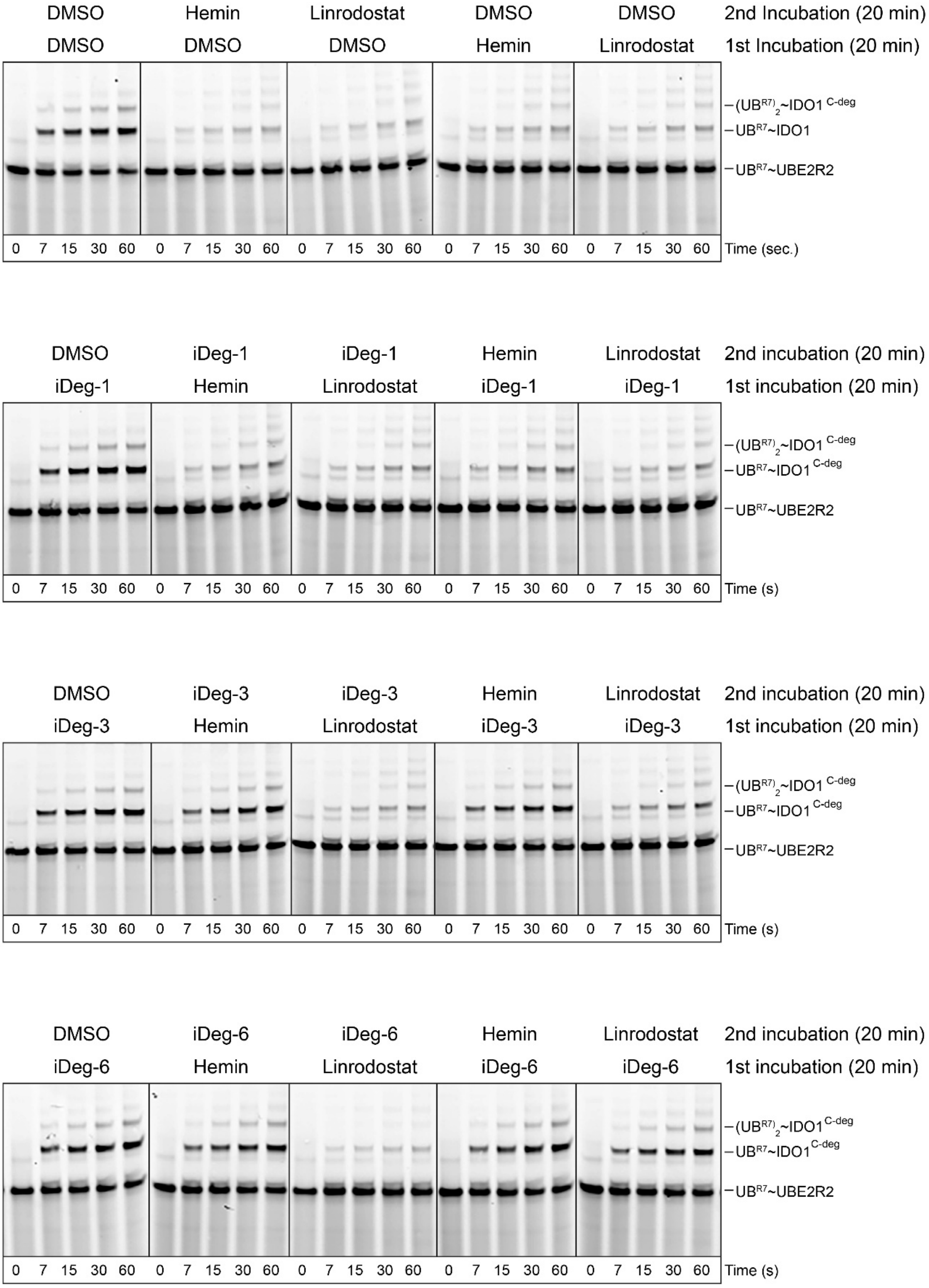

Supplementary Fig. 4: Competition ubiquitination assays monitoring the ability of full-length IDO1 bound to linrodostat or iDeg-1-6 to inhibit UBE2R2 mediated ubiquitination of the IDO1 C-terminal peptide (IDO1$^{C\text{-}deg}$) by CRL2^{KLHDC3} (n = 2). Apo-IDO1 was incubated in two sequential steps for 20 min with the indicated compounds prior to the addition NEDD8~CUL2^{KLHDC3} and initiation of the ubiquitination chase reactions using UBE2R2~UB^{R7} thioster conjugate.

Supplementary Tables

Supplementary Table 1. Changes in protein levels after treatment with iDeg-1. HEK239T cells were electroporated with rhIDO1 protein prior to treatment with 10 µM iDeg-1 or DMSO for 6 h and MS analysis.

-log(P-value)	Difference	Protein names	Gene names
6.08	-0.66	Dedicator of cytokinesis protein 8	DOCK8
**5.10**	**-0.85**	**Indoleamine 2,3-dioxygenase 1**	**IDO1**
3.13	0.78	Ras-related protein Rab-9B	RAB9B
1.29	-1.13	Rho-related BTB domain-containing protein 3	RHOBTB3
1.13	1.24	Zinc finger protein 64 homolog, isoforms 3 and 4	ZFP64

Supplementary Table 2: Data collection and refinement statistics of IDO1-iDeg-1 and IDO1-iDeg-2 structures. Values in parentheses correspond to the highest-resolution shell.

	**IDO1- iDeg-1 (PDB: 9RIS)**	**IDO1- iDeg-2 (PDB: 9FOH)**
**Crystal parameters**		
Space group	$P\ 2_1\ 2_1\ 2$	$P\ 2_1\ 2_1\ 2$
Cell dimensions		
a, *b*, *c* (Å)	105.36<br>110.21<br>36.15	104.84<br>110.42<br>36.14
α, β, γ (°)	90 90 90	90 90 90
Copies per AU[a]	1	1
**Data collection**		
Beamline	SLS X10SA	ESRF ID30B
Resolution range (Å)	47.53 – 2.1 (2.18 - 2.10)	34.73- 1.60 (1.66 - 1.60)
Total reflections	340,487 (36,211)	715,765 (62,798)
Unique reflections	47,389 (5,233)	56,429 (5,574)
R_{merge}	0.205 (1.620)	0.133 (2.317)
R_{meas}	0.221 (1.752)	0.139 (2.428)
Mean $I / \sigma I$	7.35 (1.23)	11.83 (0.99)
$CC_{1/2}$	1.00 (0.66)	1.00 (0.47)
Completeness (%)	99.71 (99.60)	99.96 (99.95)
Redundancy	7.2 (6.9)	12.7 (11.3)
**Refinement**		
Resolution (Å)	2.1	1.6
No. reflections	25,380 (2,760)	56,419 (5,572)
R_{work}	0.226 (0.400)	0.171 (0.342)
R_{free}	0.252 (0.403)	0.189 (0.366)
No. of non H-atoms	3,097	3272
Protein	2,982	2927
Ligand/ion	57	231
Water	58	243
Average B-factor	48.92	29.13
Protein	49.02	27.87
Ligand	46.60	44.16
Water	46.04	38.04
R.m.s.d.		
Bond lengths (Å)	0.009	0.12
Bond angles (°)	0.58	1.67
Ramachandran		
Favored (%)	96.12	98.00
Allowed (%)	3.88	2.00
Outliers (%)	0.00	0.00
Clashscore	3.28	4.27
Rotamer outliers (%)	1.20	0.91

Supplementary Table 3: Contribution of IDO1 degradation and IDO1 inhibition to the decrease in Kyn levels. BxPC3 cells were stimulated with IFN-γ for 24 h followed by a washout and co-treatment with carfilzomib (CFZ, 500 nM) and iDeg-6 (0.1 or 1 µM) for 7 h.

	**Total reduction in Kyn level**	**Contribution to Kyn level decrease**		**Contribution to Kyn level decrease** (Kyn level decrease set to 100%)	
		**Degradation**	**Inhibition**	**Degradation**	**Inhibition**
CFZ+iDeg-6 (0.1 µM)	37	30	7	81	19
CFZ+iDeg-6 (1 µM)	46	27	19	59	41

Supplementary Table 4: Oligo sequences for stability reporter generation and single sgRNA cloning.

Oligo	Sequence (5' ➔ 3')	Reporter
mCherry_fw	ctcggcgcgccagtcctccgagtcgacccacc atggtgagcaagggcgaggag	N-terminal stability reporter design
mCherry_rev	cacatctccggcctgcttcagcaggctgaagtt ggtggcgccgctgcctttatacaattcatccatg ccgc	N-terminal stability reporter design
BFP_fw	ctgctgaagcaggccggagatgtggaggaga accctggccccagcgagctgattaaggagaa c	N-terminal stability reporter design
BFP_rev	cagaggttgattgttccagacgcgtattaagctt gtgccccagtttg	N-terminal stability reporter design
IDO1_cterm_fw	cggcgcgccagtcctccgagtcgacgccacc atggcccacgccatgg	C-terminal IDO1 stability reporter
IDO1_cterm_rev	cacctcccgagccacctccggatcctccaccg ccctccttcagcaggctc	C-terminal IDO1 stability reporter
IDO1_nterm_fw	tactgcgacctccctagcaaactggggcacaa gcttaatacgcgtatggcccacgccatgg	N-terminal IDO1 stability reporter
IDO1_nterm_WT_rev	ctttcacaaattttgtaatccagaggttgattgttc cagatcagccctccttcagcaggctcttctcggt ggtg	N-terminal IDO1 stability reporter
IDO1_nterm_RG_rev	ctttcacaaattttgtaatccagaggttgattgttc cagatcagccTCTcttcagcaggctcttctcg gtggtg	Degron mutant "-RG" N-terminal IDO1 stability reporter
IDO1_nterm_EK_rev	ctttcacaaattttgtaatccagaggttgattgttc cagatcaCTTctccttcagcaggctcttctcgg tggtg	Degron mutant "-EK" N-terminal IDO1 stability reporter
sgRNA_KLHDC3_1_fw	caccGCATCGGGTATACTCCTTC G	Exon 2 targeting sgRNA for KLHDC3 (corresponds to KO1)
sgRNA_KLHDC3_1_rev	aaacCGAAGGAGTATACCCGATG C	Exon 2 targeting sgRNA for KLHDC3 (corresponds to KO1)
sgRNA_KLHDC3_2_fw	caccGGTGTCAAAGACTCGAATG	Exon 6 targeting sgRNA for KLHDC3 (corresponds to KO2)
sgRNA_KLHDC3_2_rev	aaacCATTCGAGTCTTTGACACC	Exon 6 targeting sgRNA for KLHDC3 (corresponds to KO2)
sgRNA_AAVS1_fw	caccGCTGTGCCCCGATGCACAC	CTRL sgRNA
sgRNA_AAVS1_rev	aaacGTGTGCATCGGGGCACAGC	CTRL sgRNA

Chemistry

General Experimental Procedures

Reactions were performed in heat gun dried flasks under an argon atmosphere. All reagents purchased from commercial sources were used directly without further purification.
Thin-layer chromatography was performed on pre-coated silica gel F-254 plates from Merck. The spots were visualized by UV light and/or by staining of the TLC plate with potassium permanganate stain followed, if necessary, by heating with a heat gun. For column chromatography, silica gel 60 from Merck with a particle size of 0.040-0.063 mm was used.
^{1}H- and ^{13}C-NMR spectra were recorded on Bruker AV 400 Avance III HD (400 MHz), Bruker AV 500 Avance III HD (500 MHz), Bruker AV 600 Avance III HD (600 MHz) and Bruker AV 700 Avance III HD (700 MHz) spectrometers at room temperature.
HPLC-MS analyses were performed with an Agilent 1100 Series connected to a Thermo LCQ Advantage mass spectrometer using a C18 HPLC column 3 µm from Macherey Nagel.
High resolution mass spectra were recorded on a QLTOrbitrap mass spectrometer coupled to an Acceka HPLC-System (HPLC column: Hypersyl GOLD, 50 mm × 1 mm particle size, 1.9 µm).

For the synthesis of iDegs, (1*R*)-(−)-myrtenal (**1**, 218243, Sigma-Aldrich) was subjected to a 1,3-dipolar cycloaddition with the azomethine ylide generated *in situ* from *N*-(methoxymethyl)-*N*-(trimethylsilylmethyl) benzylamine by treatment with trifluoroacetic acid to yield the corresponding pyrrolidine cycloadduct (**2**) (Supplementary Scheme 1a) analogous to previously reported comparable cycloadditions (Mutti *et al.* and Sheffler. *et al.*)[1,2]. Subsequently, the aldehyde (**2**) was reduced to the corresponding alcohol **3** (Supplementary Scheme 1b). For synthesis of iDeg-1, the alcohol was further functionalized as urethane, followed by *N*-debenzylation and sulfonamide formation (Supplementary Scheme 2). For synthesis of iDeg-2, -3, and -4, the *N*-benzyl protecting group was removed from **3** to yield amine **7** followed by sulfonamide formation (⇨ **8, 9**) and functionalization of the alcohol as urethanes **10** (iDeg-2) and **11** (Supplementary Scheme 3). Subsequently, the aryl iodide in iDeg-2 (**10)** and in urethane **11** were subjected to a Pd(0) mediated Sonogashira coupling reaction to yield **12** (iDeg-3) and **13** (iDeg-6) respectively (Supplementary Scheme 3).

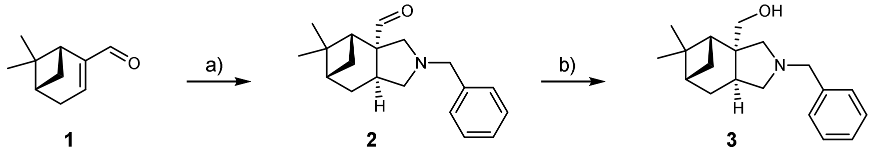

Supplementary Scheme 1: a) *N*-(Methoxymethyl)-*N*-(trimethylsilylmethyl) benzylamine, TFA, DCM, 0 °C to rt, 16 h; b) $NaBH_4$, MeOH, 0 °C to rt, 1 h

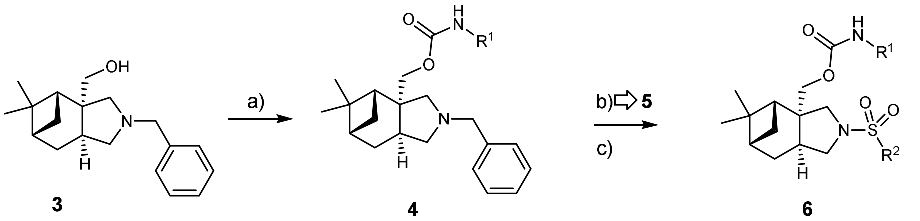

3

4
R^1 = 4-H_3C-Ph-

6
R^1 = 4-H_3C-Ph-
R^2 = 4-*t*Bu-Ph-
iDeg-1

Supplementary Scheme 2. a) R^1NCO, NEt_3, THF, rt, 2 h; b) Pd/C, NH_4HCO_2, MeOH, 60 °C, 1 h or Pd/C, H_2, EtOH/THF, rt, 18 h; c) R^2SO_2Cl, NEt_3, DCM, 0 °C to rt, 2 h.

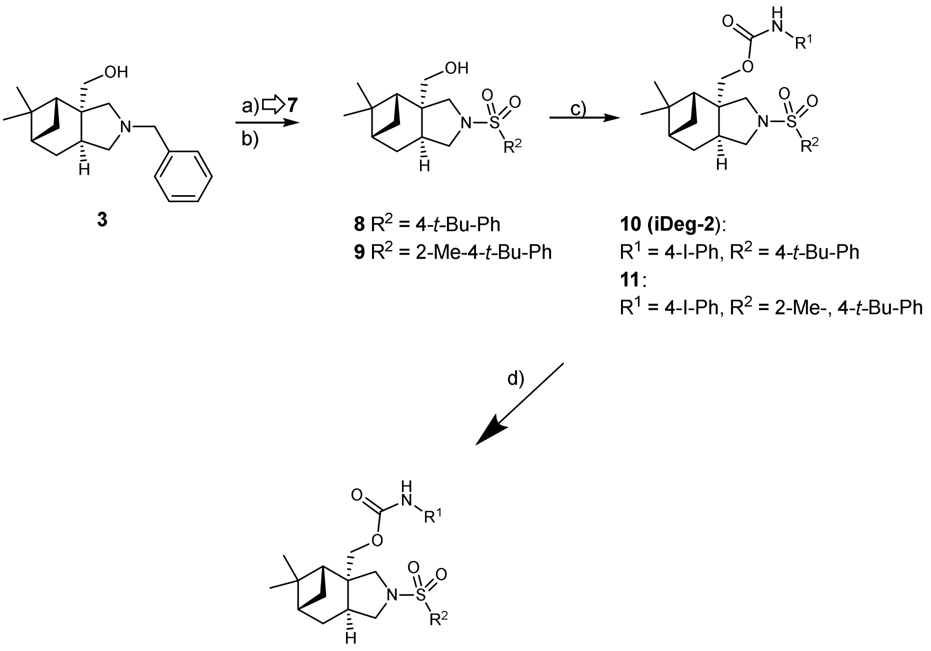

3

8 R^2 = 4-*t*-Bu-Ph
9 R^2 = 2-Me-4-*t*-Bu-Ph

10 (iDeg-2):
R^1 = 4-I-Ph, R^2 = 4-*t*-Bu-Ph
11:
R^1 = 4-I-Ph, R^2 = 2-Me-, 4-*t*-Bu-Ph

12 (iDeg-3):
R^1 = 4-CCH, R^2 = 4-*t*-Bu-Ph
13 (iDeg-6):
R^1 = 4-CCH, R^2 = 2-Me-, 4-*t*-Bu-Ph

Supplementary Scheme 3. a) Pd/C, H_2, MeOH, rt, 16 h; b) R^2SO_2Cl, NEt_3, DCM, 0 °C to rt, 2 h; c) R^1NCO, DBU, THF, rt, 18 h; d) Me_3SiCCH, NEt_3, THF, $Pd(PPh_3)_4$, then TBAF.

General procedure A: carbamate formation

To a solution of the alcohol (1.0 equiv.) in THF (0.2 M) was added NEt_3 (2.0 equiv.) and the respective isocyanate (1.3 equiv.) and the solution was stirred at room temperature for 2 h.

The solvent was removed under reduced pressure. Column chromatography afforded the carbamate.

General procedure B: benzyl group cleavage

B1:

To a degassed solution of the *N*-benzyl protected amine (1.0 equiv.) in MeOH (0.1−0.2 M) was added ammonium formate (5.0 equiv.) and Pd/C (10 wt.%) and the suspension was stirred at 60 °C for 1 h. After cooling to room temperature, the reaction mixture was filtered over Celite, washed with EtOAc and the solvent was removed under reduced pressure to give the amine.

B2:

To a degassed solution of the *N*-benzyl-protected amine (1.0 equiv.) in MeOH (0.1 M) was added Pd/C (10 wt.%) and the suspension was stirred under a H_2 atmosphere for 16−22 h. The reaction mixture was filtered over Celite, washed with EtOAc and the solvent was removed under reduced pressure to give the amine.

General procedure C: sulfonamide formation

To a solution of the amine (1.0 equiv.) in dichloromethane (0.2 M) at 0 °C was added NEt_3 (2.0 equiv.) and the respective sulfonyl chloride (1.2 equiv.) and the solution was stirred at room temperature for 2 h. The solvent was removed under reduced pressure. Column chromatography afforded the sulfonamide.

General procedure D: carbamate formation

To a solution of the alcohol (1.0 equiv.) in THF (0.1−0.2 M) was added DBU (1.5 equiv.) and the respective isocyanate (1.2 equiv.) and the solution was stirred at room temperature for 18-22 h. The solvent was removed under reduced pressure. Column chromatography afforded the carbamate.

(3a*S*,4*R*,6*R*,7a*S*)-2-benzyl-5,5-dimethyloctahydro-3a*H*-4,6-methanoisoindole-3a-carbaldehyde (2)

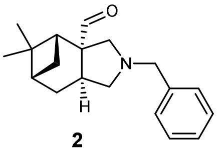

2

To a solution of myrtenal (**1**) (2.0 g, 13.3 mmol, 1 eq.) and *N*-(methoxymethyl)-*N*-trimethylsilylmethyl) benzylamine 1 (4.5 mL, 15.9 mmol, 1.2 eq., 90% pure) in 65 mL dry CH_2Cl_2 under argon atmosphere in an ice bath at 0°C, a solution of TFA in dry DCM (0.2 mL, 2.6 mmol, 0.2 eq., 0.1 M) was added dropwise over 1 min. The mixture was stirred for 12 h at 5-10°C. Progress of the reaction was monitored by TLC. After complete consumption of the starting material the reaction was quenched by the addition of aqueous saturated sodium bicarbonate and the mixture was stirred for 5 min. The layers were separated and the aqueous layer was extracted with dichloromethane. The combined organic layers were dried over Na_2SO_4, filtered, and concentrated under reduced pressure to give a crude residue, which was purified by chromatography (PE:EtOAc, 4:1, v/v). The product was obtained as colorless oil (2.8 g, 9.8 mmol, 74 % yield).

TLC (PE: EtOAc, 6:1, v/v): Rf=0.6.

^{1}H NMR (400 MHz, $CDCl_3$): δ 9.69 (s, 1H), 7.38 – 7.13 (m, 5H), 3.65 (d, *J* = 13.1 Hz, 1H), 3.50 (d, *J* = 13.1 Hz, 1H), 3.18 (t, *J* = 8.4 Hz, 1H), 2.92 (dtd, *J* = 10.7, 7.8, 3.0 Hz, 1H), 2.69 (d, *J* = 10.2 Hz, 1H), 2.43 (d, *J* = 10.2 Hz, 1H), 2.35 – 2.08 (m, 4H), 1.88 (tt, *J* = 6.0, 3.1 Hz, 1H), 1.69 – 1.57 (m, 2H), 1.24 (s, 3H), 0.69 (s, 3H).

^{13}C NMR (101 MHz, $CDCl_3$): δ 206.1, 138.9, 128.6, 128.4, 127.1, 66.2, 61.2, 60.9, 59.7, 45.8, 40.5, 38.7, 32.2, 31.0, 26.6, 26.5, 23.7.

HRMS (ESI) for $C_{19}H_{26}ON$: calculated 284.2009 $[M+H]^+$, found: 284.2015.

((3a*S*,4*R*,6*R*,7a*S*)-2-benzyl-5,5-dimethyloctahydro-3a*H*-4,6-methanoisoindol-3a-yl)methanol (3)

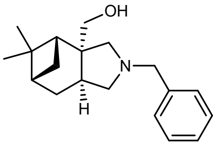

3

To a solution of **2** (1.0 g, 3.5 mmol, 1 eq.) in MeOH (10 mL) at 0°C $NaBH_4$ (173 mg, 4.5 mmol, 1.3 eq.) was added in portions. After stirring for 1 h, the reaction was quenched with acetone (5 mL). The solvents were removed under reduced pressure and the residue was dissolved in water. The aqueous layer was extracted twice with ethyl acetate, the combined organic layers were dried over Na_2SO_4 and concentrated under reduced pressure. The crude product was purified by chromatography (PE:EtOAc, 4:1, v/v) and the product was obtained as colorless oil (0.92 g, 3.2 mmol, 92 % yield).

TLC (PE: EtOAc, 3:1, v/v): Rf=0.3; $[\alpha]^{20}_D$ = -53.7° ($CHCl_3$, *c* = 1.0)

^{1}H NMR (500 MHz, $CDCl_3$) δ 7.32 (d, *J* = 4.5 Hz, 4H), 7.30 – 7.25 (m, 1H), 3.81 (d, *J* = 9.9 Hz, 1H), 3.68 (t, *J* = 10.8 Hz, 2H), 3.39 (d, *J* = 9.9 Hz, 1H), 3.36 (d, *J* = 8.6 Hz, 1H), 2.86 (d, *J* = 9.3 Hz, 1H), 2.59 – 2.52 (m, 1H), 2.50 (d, *J* = 9.5 Hz, 1H), 2.23 – 2.17 (m, 1H), 2.18 – 2.11 (m, 1H), 2.11 (d, *J* = 13.9 Hz, 1H), 1.89 (dq, *J* = 5.9, 3.0 Hz, 1H), 1.75 (dd, *J* = 6.4, 4.9 Hz, 1H), 1.56 (d, *J* = 13.5 Hz, 1H), 1.40 (d, *J* = 10.4 Hz, 1H), 1.22 (s, 3H), 0.95 (s, 3H).

^{13}C NMR (126 MHz, $CDCl_3$) δ 137.4, 129.0, 128.7, 127.7, 71.4, 66.4, 64.2, 59.7, 51.0, 46.2, 41.3, 38.9, 32.9, 32.1, 27.6, 27.6, 24.4.

HRMS (ESI) for $C_{19}H_{28}ON$; calculated 286.2165 $[M+H]^+$, found 286.2167.

((3a*S*,4*R*,6*R*,7a*S*)-2-benzyl-5,5-dimethyloctahydro-3a*H*-4,6-methanoisoindol-3a-yl)methyl *p*-tolylcarbamate (4)

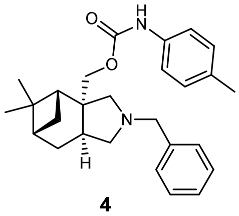

4

Following general procedure A: From **3** (100 mg, 0.35 mmol) and *p*-tolyl isocyanate. Purification by column chromatography (PE:EtOAc, 9:1, v/v) afforded a colorless foam (122 mg, 0.29 mmol, 83 % yield).

TLC (PE: EtOAc, 3:1, v/v): Rf=0.7.

^{1}H NMR ($CDCl_3$, 500 MHz): δ 7.20–7.39 (m, 7 H), 7.10 (d, *J* = 8.1 Hz, 2 H), 6.46 (s_{br}, 1 H), 4.21 (d, *J* = 10.3 Hz, 1 H), 4.05 (d, *J* = 10.3 Hz, 1 H), 3.63 (d, *J* = 13.3 Hz, 1 H), 3.55 (d, *J* = 13.1 Hz, 1 H), 2.57–2.62 (m, 1 H), 2.46–2.52 (m, 1 H), 2.34 (d, *J* = 9.4 Hz, 1 H), 2.30 (s, 3 H), 2.21–2.28 (m, 1 H), 2.06–2.19 (m, 2 H), 1.95 (t, *J* = 5.7 Hz, 1 H), 1.82–1.89 (m, 2 H), 1.60 (dt, *J* = 3.5, 13.3 Hz, 1 H), 1.23 (s, 3 H), 1.03 (s, 3 H).

^{13}C NMR ($CDCl_3$, 125 MHz): δ 154.0, 139.9, 135.5, 133.01, 129.7, 128.6, 128.3, 126.8, 118.7, 71.0, 64.2, 50.5, 46.3, 40.6, 39.5, 34.9, 34.6, 29.9, 27.6, 27.1, 26.7, 23.6, 20.9.

HRMS (ESI) for $C_{27}H_{35}N_2O_2$: calculated 419.2693 $[M+H]^+$, found 419.12692.

((3a*S*,4*R*,6*R*,7a*S*)-5,5-dimethyloctahydro-3a*H*-4,6-methanoisoindol-3a-yl)methyl *p*-tolylcarbamate (5)

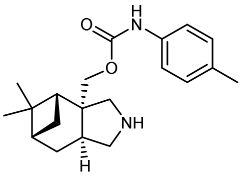

5

Following general procedure B1: From **4** (1.0 g, 2.4 mmol) afforded a colorless solid (746 mg, 2.3 mmol, 95 % yield).

^{1}H NMR ($CDCl_3$, 400 MHz): δ = 7.33 (d, *J* = 8.2 Hz, 2H), 7.08 (d, *J* = 8.2 Hz, 2H), 4.28 (d, *J* = 10.7 Hz, 1H), 4.07 (d, *J* = 10.7 Hz, 1H), 3.75 – 3.61 (m, 1H), 3.41 – 3.28 (m, 2H), 3.11 (d, *J* = 12.3 Hz, 1H), 2.95 (dd, *J* = 11.8, 7.5 Hz, 1H), 2.51 – 2.22 (m, 3H), 2.27 (s, 3H), 2.04 (t, *J* = 5.6 Hz, 1H), 1.96 (d, *J* = 5.4 Hz, 1H), 1.65 (dt, *J* = 13.8, 3.2 Hz, 1H), 1.28 (d, 3H), 1.06 (s, 3H).

HRMS (ESI) for $C_{20}H_{29}N_2O_2$: calculated 329,222 $[M+H]^+$, found: 329.224

((3a*S*,4*R*,6*R*,7a*S*)-2-((4-(*tert*-butyl)phenyl)sulfonyl)-5,5-dimethyloctahydro-3a*H*-4,6-methanoisoindol-3a-yl)methyl *p*-tolylcarbamate(6) (iDeg-1)

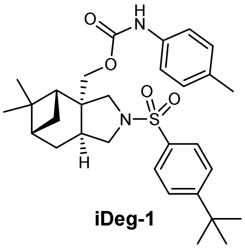

iDeg-1

Following general procedure B1: From **4** (110 mg, 0.26 mmol) afforded a colorless solid **5** (81.0 mg, 0.25 mmol, 94 % yield).
Following general procedure C: From amine **5** (81.0 mg, 0.25 mmol) and 4-*tert*-butylbenzenesulfonyl chloride. Purification by column chromatography (PE:EtOAc, 8:1 → 4:1, v/v) afforded a colorless foam (115 mg, 0.22 mmol, 89 % yield).

TLC (PE: EtOAc, 3:1, v/v): Rf=0.6; $[\alpha]^{20}_D$ = -16.0° ($CHCl_3$, *c* = 0.5)

^{1}H NMR (600 MHz, CD_2Cl_2) δ 7.71 (d, *J* = 8.5 Hz, 2H), 7.56 (d, *J* = 8.6 Hz, 2H), 7.19 (d, *J* = 7.6 Hz, 2H), 7.10 (d, *J* = 7.9 Hz, 2H), 6.36 (s, 1H), 4.06 (d, *J* = 10.7 Hz, 1H), 3.92 (d, *J* = 10.6

Hz, 1H), 3.15 (dd, J = 9.2, 7.1 Hz, 1H), 3.05 (d, J = 9.9 Hz, 1H), 3.02 (dd, J = 9.2, 3.1 Hz, 1H), 2.99 (d, J = 9.9 Hz, 1H), 2.33 (ddd, J = 10.5, 3.3, 2.1 Hz, 1H), 2.30 (s, 3H), 2.25 (tdd, J = 7.4, 4.2, 2.2 Hz, 1H), 2.20 (ddt, J = 8.4, 6.2, 3.1 Hz, 1H), 1.98 (dd, J = 6.5, 5.0 Hz, 1H), 1.87 (tt, J = 5.6, 3.0 Hz, 1H), 1.63 – 1.59 (m, 1H), 1.32 (d, J = 10.4 Hz, 1H), 1.30 (s, 9H), 1.22 (s, 3H), 1.01 (s, 3H).

^{13}C NMR (151 MHz, CD_2Cl_2) δ 157.2, 153.7, 135.6, 133.6, 132.4, 129.9, 128.3, 126.4, 119.2, 70.1, 58.5, 57.4, 54.2, 54.0, 53.8, 53.7, 53.5, 50.9, 46.7, 40.6, 39.4, 35.4, 34.7, 34.5, 31.2, 27.4, 27.3, 23.5, 20.8.

HRMS (ESI) for $C_{30}H_{41}N_2O_4S$: calculated 525.2782 $[M+H]^+$, found 525.2785.

((3a*S*,4*R*,6*R*,7a*S*)-2-((4-(*tert*-butyl)phenyl)sulfonyl)-5,5-dimethyloctahydro-3a*H*-4,6-methanoisoindol-3a-yl)methanol (8)

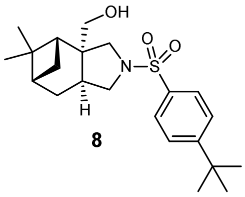

8

To a degassed solution of **3** (389 mg, 1.4 mmol) in MeOH (4 mL) was added Pd/C (10 wt%, 40 mg) and the mixture was stirred under a H_2 atmosphere for 16 h. The reaction mixture was filtered over Celite, washed with MeOH and the solvent was evaporated to give intermediate **7** a colorless solid (242 mg, 1.2 mmol, 91 % yield) which was used immediately for the following transformation.

To a solution of the solid **7** (242 mg, 1.2 mmol) in THF (10 mL) at 0 °C was added NEt_3 (198 µL, 1.5 mmol, 1.2 eq.) and 4-*tert*-butylbenzenesulfonyl chloride (288 mg, 1.24 mmol, 1.0 eq.) and the mixture was stirred at 0 °C for 2 h. The solvent was removed under reduced pressure. Column chromatography (PE:EtOAc, 4:1 → 2:1, v/v) afforded a colorless solid 8 (415 mg, 1.1 mmol, 86 % yield).

TLC (PE: EtOAc, 3:1, v/v): Rf=0.4; $[\alpha]^{20}_D$ = - 25.3° ($CHCl_3$, c = 1.0)

^{1}H NMR (500 MHz, $CDCl_3$) δ 7.72 (d, J = 8.6 Hz, 2H), 7.53 (d, J = 8.6 Hz, 2H), 3.51 (d, J = 10.5 Hz, 1H), 3.42 (d, J = 10.6 Hz, 1H), 3.19 (dd, J = 9.1, 7.3 Hz, 1H), 3.06 (d, J = 9.7 Hz, 1H), 3.01 – 2.95 (m, 2H), 2.31 – 2.23 (m, 1H), 2.21 – 2.14 (m, 2H), 1.96 (dd, J = 6.5, 5.0 Hz, 1H), 1.86 (dq, J = 5.4, 2.8 Hz, 1H), 1.61 (ddd, J = 13.4, 4.4, 2.8 Hz, 1H), 1.35 (s, 9H), 1.31 (d, J = 10.4 Hz, 1H), 1.21 (s, 3H), 0.95 (s, 3H).

^{13}C NMR (126 MHz, $CDCl_3$) δ 156.6, 132.0, 128.1, 126.0, 68.2, 58.5, 56.9, 52.3, 45.8, 40.4, 39.2, 35.3, 34.2, 34.1, 31.2, 27.5, 27.2, 23.7.

HRMS (ESI) for $C_{22}H_{34}NO_3S$: calculated 392.2254 $[M+H]^+$, found 392.2254.

((3a*S*,4*R*,6*R*,7a*S*)-2-((4-(*tert*-butyl)-2-methylphenyl)sulfonyl)-5,5-dimethyloctahydro-3a*H*-4,6-methanoisoindol-3a-yl)methanol (9)

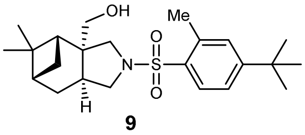

9

To a solution of the solid **7** (58.6 mg, 0.3 mmol) in THF (10 mL) at 0 °C was added NEt_3 (55.5 µL, 0.4 mmol, 1.2 eq.) and 4-(*tert*-butyl)-2-methylbenzenesulfonyl chloride (74.0 mg, 1.0 eq.) and the mixture was stirred at 0 °C for overnight. The solvent was removed under reduced pressure. Column chromatography (PE:EtOAc, 4:1 → 2:1, v/v) afforded a colorless solid (80 mg, 0.2 mmol, 66 % yield).

TLC (PE: EtOAc, 3:1, v/v): Rf=0.4; $[\alpha]^{20}_D$ = -88.3° ($CHCl_3$, *c* = 0.5)

^{1}H NMR (500 MHz, $CDCl_3$) δ 7.83 (d, *J* = 8.1 Hz, 1H), 7.33 – 7.28 (m, 2H), 3.57 (d, *J* = 10.6 Hz, 1H), 3.50 (d, *J* = 10.6 Hz, 1H), 3.42 (dd, *J* = 9.3, 7.0 Hz, 1H), 3.25 (d, *J* = 9.8 Hz, 1H), 3.03 (dd, *J* = 9.4, 3.7 Hz, 1H), 2.99 (d, *J* = 9.7 Hz, 1H), 2.66 (s, 3H), 2.27 (ddt, *J* = 12.1, 7.6, 2.1 Hz, 1H), 2.23 – 2.16 (m, 2H), 2.00 – 1.94 (m, 1H), 1.87 (dt, *J* = 5.4, 2.7 Hz, 1H), 1.59 (d, *J* = 3.2 Hz, 1H), 1.33 (s, 9H), 1.31 (d, *J* = 10.5 Hz, 1H), 1.22 (s, 3H), 0.97 (s, 3H).

^{13}C NMR (126 MHz, $CDCl_3$) δ 156.6, 138.0, 132.6, 130.4, 129.9, 123.2, 68.3, 58.0, 56.3, 52.4, 45.8, 40.5, 39.2, 35.0, 34.3, 34.1, 31.2, 27.6, 27.3, 23.8, 21.3.

HRMS (ESI) for $C_{23}H_{36}NO_3S$: calculated 406.2410 $[M+H]^+$, found 406.2408.

((3a*S*,4*R*,6*R*,7a*S*)-2-((4-(*tert*-butyl)phenyl)sulfonyl)-5,5-dimethyloctahydro-3a*H*-4,6-methanoisoindol-3a-yl)methyl (4-iodophenyl)carbamate (10) (iDeg-2)

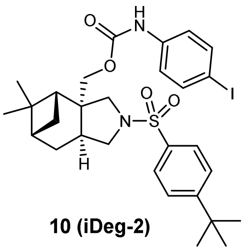

10 (iDeg-2)

Following general procedure D: From **8** (20.0 mg, 51.1 µmol) and 4-iodophenyl isocyanate (15 mg, 61.3 µmol, 1.2 eq.). Purification by column chromatography (PE:EtOAc, 4:1, v/v) afforded a colorless solid (28.0 mg, 44.0 µmol, 86 % yield).

TLC (PE: EtOAc, 3:1, v/v): Rf=0.6; $[\alpha]^{20}_D$ = -10.7° ($CHCl_3$, *c* = 0.5)

^{1}H NMR (500 MHz, CD_2Cl_2) δ 7.71 (d, *J* = 8.5 Hz, 2H), 7.61 (d, *J* = 8.8 Hz, 2H), 7.56 (d, *J* = 8.6 Hz, 2H), 7.15 (d, *J* = 8.3 Hz, 2H), 6.54 (s, 1H), 4.11 (d, *J* = 10.7 Hz, 1H), 3.93 (d, *J* = 10.7 Hz, 1H), 3.20 (dd, *J* = 9.3, 7.1 Hz, 1H), 3.04 – 2.96 (m, 3H), 2.37 – 2.28 (m, 1H), 2.28 – 2.17 (m, 2H), 1.98 (dd, *J* = 6.5, 4.9 Hz, 1H), 1.87 (s, 1H), 1.63 – 1.58 (m, 1H), 1.31 (s, 9H), 1.28 (s, 1H), 1.22 (s, 3H), 1.01 (s, 3H).

^{13}C NMR (126 MHz, CD_2Cl_2) δ 157.2, 153.4, 138.3, 138.2, 132.5, 128.3, 126.4, 120.9, 86.5, 70.4, 58.5, 57.3, 50.8, 46.6, 40.6, 39.4, 35.4, 34.7, 34.3, 31.2, 27.4, 27.2, 23.5.

HRMS (ESI) for $C_{29}H_{38}IN_2O_4S$: calculated 637.1592 $[M+H]^+$, found 637.1601.

((3a*S*,4*R*,6*R*,7a*S*)-2-((4-(*tert*-butyl)-2-methylphenyl)sulfonyl)-5,5-dimethyloctahydro-3a*H*-4,6-methanoisoindol-3a-yl)methyl (4-iodophenyl)carbamate (11)

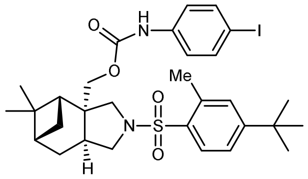

11

Following general procedure D: From **9** (60.84 mg, 0.15 mmol, 1.0 eq.) and 1-iodo-4-isocyanatobenzene (73.5 mg, 0.23 mmol, 1.5 eq.). Purification by column chromatography (PE:EtOAc, 20:1 to 10:1, v/v) afforded a colorless solid **11** (69.6 mg, 0.11 mmol, 71 % yield).

TLC (PE: EtOAc, 3:1, v/v): Rf=0.6; $[\alpha]^{20}_D$ = +11.5° ($CHCl_3$, *c* = 0.5)

^{1}H NMR (500 MHz, $CDCl_3$) δ 7.85 (d, *J* = 8.7 Hz, 1H), 7.60 (d, *J* = 8.8 Hz, 2H), 7.33 – 7.28 (m, 2H), 7.18 (d, *J* = 8.4 Hz, 2H), 6.90 (s, 1H), 4.18 (d, *J* = 10.7 Hz, 1H), 4.05 (d, *J* = 10.6 Hz, 1H), 3.48 (t, *J* = 8.4 Hz, 1H), 3.37 (d, *J* = 10.0 Hz, 1H), 3.04 (d, *J* = 10.0 Hz, 1H), 3.00 (dd, *J* = 9.5, 3.5 Hz, 1H), 2.66 (s, 3H), 2.35 (dt, *J* = 7.3, 3.5 Hz, 1H), 2.34 – 2.26 (m, 1H), 2.21 (dtd, *J* = 10.4, 6.3, 1.9 Hz, 1H), 1.95 – 1.87 (m, 2H), 1.57 – 1.53 (m, 1H), 1.31 (s, 9H), 1.29 (d, *J* = 8.5 Hz, 1H), 1.23 (s, 3H), 1.03 (s, 3H).

^{13}C NMR (176 MHz, CD_2Cl_2) δ 157.1, 153.5, 138.4, 138.3, 138.3, 133.0, 130.4, 130.3, 123.5, 120.8, 86.4, 70.6, 58.0, 56.6, 51.0, 46.7, 40.6, 39.3, 35.2, 34.8, 34.4, 31.1, 27.4, 27.4, 23.6, 21.2.

HRMS (ESI) for $C_{30}H_{39}IN_2O_4SNa$: calculated 651.1748 $[M+Na]^+$, found 651.1752.

((3a*S*,4*R*,6*R*,7a*S*)-2-((4-(*tert*-butyl)phenyl)sulfonyl)-5,5-dimethyloctahydro-3a*H*-4,6-methanoisoindol-3a-yl)methyl (4-ethynylphenyl)carbamate(12) (iDeg-3)

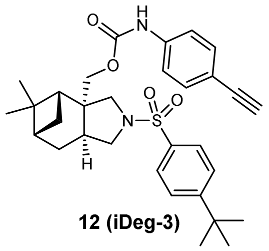

12 (iDeg-3)

To a degassed solution of **10** (60.0 mg, 94.3 µmol) in THF (0.5 mL) was added NEt_3 (65 µL, 0.471 mmol, 5.0 eq.), trimethylsilylacetylene (26 µL, 0.189 mmol, 2.0 eq.), CuI (3.6 mg, 18.9 µmol, 0.2 eq.) and Pd $(PPh_3)_4$ (10.9 mg, 9.43 µmol, 0.1 eq.) and the mixture was stirred at room temperature for 22 h. The reaction mixture was poured into sat. aq. NaCl solution, extracted with dichloromethane (2 × 20 mL), dried over $MgSO_4$ and the solvent was removed under reduced pressure. Column chromatography (PE:EtOAc, 6:1, v/v) afforded a beige colored solid (50.0 mg).

To a solution of the solid in THF (0.5 mL) was added TBAF (1 M in THF, 99 µL, 98.9 µmol, 1.2 eq.) and the mixture was stirred at room temperature for 1 h. The solvent was removed under reduced pressure. Column chromatography (PE:EtOAc, 5:1, v/v) afforded a colorless solid (25.0 mg, 46.8 µmol, 50 % yield over 2 steps).

TLC (PE: EtOAc, 3:1, v/v): Rf=0.6; $[\alpha]^{20}_D$ = -13.3° ($CHCl_3$, *c* = 0.5).

^{1}H NMR (500 MHz, CD_2Cl_2) δ 7.72 (d, J = 8.6 Hz, 2H), 7.56 (d, J = 8.6 Hz, 2H), 7.43 (d, J = 8.7 Hz, 2H), 7.33 (d, J = 8.4 Hz, 2H), 6.63 (s, 1H), 4.12 (d, J = 10.7 Hz, 1H), 3.94 (d, J = 10.7 Hz, 1H), 3.21 (dd, J = 9.3, 7.1 Hz, 1H), 3.09 (s, 1H), 3.05 – 2.97 (m, 3H), 2.32 (tdd, J = 13.0, 3.1, 2.0 Hz, 1H), 2.26 (tt, J = 7.0, 3.6 Hz, 1H), 2.20 (dtd, J = 10.4, 6.3, 2.0 Hz, 1H), 1.98 (dd, J = 6.5, 5.0 Hz, 1H), 1.88 (tt, J = 5.7, 3.1 Hz, 1H), 1.63 – 1.59 (m, 1H), 1.31 (s, 9H), 1.28 (d, J = 9.9 Hz, 1H), 1.23 (s, 3H), 1.01 (s, 3H).

^{13}C NMR (126 MHz, CD_2Cl_2) δ 157.2, 153.3, 142.6, 138.9, 133.3, 132.5, 128.3, 126.4, 118.5, 117.1, 83.6, 76.8, 70.4, 58.5, 57.3, 50.8, 46.6, 40.6, 39.4, 35.5, 34.7, 34.3, 31.2, 27.4, 27.2, 23.5.**HRMS** (ESI) for $C_{31}H_{39}N_2O_4S$: calculated 535.2625 $[M+H]^+$, found 535.2627.

((3a*S*,4*R*,6*R*,7a*S*)-2-((4-(*tert*-butyl)-2-methylphenyl)sulfonyl)-5,5-dimethyloctahydro-3a*H*-4,6-methanoisoindol-3a-yl)methyl (4-ethynylphenyl)carbamate 13 (iDeg-6)

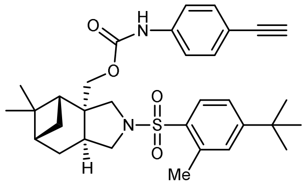

13 (iDeg-6)

To a degassed solution of **11** (89 mg, 0.14 mmol) in THF (1.4 mL) was added NEt_3 (98.3 µL, 0.7 mmol, 5.0 eq.), Trimethylsilylacetylene (42 ul, 0.28 mmol, 2.0 eq.), CuI (5.3 mg, 0.028 mmol, 0.2 eq.) and $Pd(PPh_3)_4$ (16.2 mg, 0.014 mmol, 0.1 eq.), and the mixture was stirred at room temperature for 22 h. The reaction mixture was poured into sat. aq. NaCl solution, extracted with dichloromethane (2 ×), dried over $MgSO_4$ and the solvent was removed under reduced pressure. Column chromatography (PE:EtOAc, 15:1 to 10:1 to 5:1, v/v) afforded a beige colored solid.

To a solution of the solid in THF (1.8 mL) was added TBAF (1 M in THF, 180 µL, 0.18 mmol, 1.2 eq.) and the mixture was stirred at room temperature for 1 h. The solvent was removed under reduced pressure. Column chromatography (PE:EtOAc, 15:1 to 10:1 to 5:1, v/v) afforded a light pink solid (40.7 mg, 0.07 mmol, 53 % yield over 2 steps, >99% HPLC purity).

TLC (PE: EtOAc, 3:1, v/v): Rf=0.6; $[\alpha]^{20}_{D}$ = +10.7° ($CHCl_3$, *c* = 0.5).

^{1}H NMR (600 MHz, CD_2Cl_2) δ 7.81 (d, *J* = 9.0 Hz, 1H), 7.45 – 7.41 (m, 2H), 7.36 (d, *J* = 8.4 Hz, 2H), 7.34 (d, *J* = 7.2 Hz, 2H), 6.95 (s, 1H), 4.10 (d, *J* = 2.7 Hz, 2H), 3.45 – 3.39 (m, 1H), 3.27 (d, *J* = 10.0 Hz, 1H), 3.09 (s, 1H), 3.02 (d, *J* = 10.0 Hz, 1H), 3.00 (dd, *J* = 10.3, 2.0 Hz, 1H), 2.64 (s, 3H), 2.32 (t, *J* = 3.1 Hz, 2H), 2.25 – 2.19 (m, 1H), 1.99 – 1.94 (m, 1H), 1.89 (dd, *J* = 5.5, 2.2 Hz, 1H), 1.30 (s, 9H), 1.29 (d, *J* = 5.7 Hz, 1H), 1.23 (s, 3H), 1.03 (s, 3H).

^{13}C NMR (151 MHz, CD_2Cl_2) δ 157.1, 153.4, 139.1, 138.3, 133.3, 133.0, 130.4, 130.3, 123.5, 118.5, 117.0, 83.6, 76.8, 70.6, 58.0, 56.6, 51.0, 46.7, 40.6, 39.3, 35.2, 34.9, 34.4, 31.1, 27.4, 27.4, 23.6, 21.2.

HRMS (ESI) for $C_{32}H_{40}N_2O_4SNa$: calculated: 571.2606 $[M+Na]^+$, found 571.2602.

General procedure for the synthesis of benzenesulfonyl chlorides (14):[3]

Chlorosulfonic acid (5 mL, 5 eq.) was added dropwise to a solution of alkyl benzene (15 mmol, 1 eq.) in chloroform at 0 °C. The resulting solution was stirred at room temperature for 1 h. The reaction mixture was poured onto ice, and the aqueous phase was extracted with chloroform twice. After the reaction was finished, the mixture was carefully poured over crushed ice and the aqueous layer was extracted with chloroform. The combined organic layers were then washed with brine, dried over sodium sulfate, and concentrated by rotary evaporation to give the crude product. The resulting oil was then purified by flash column chromatography (hexanes/ethyl acetate = 20:1, v/v; 1.3 g, 5.3 mmol, 35 % yield).

2-Methyl-4-(*tert*-butyl)-benzenesulfonyl chloride (14)

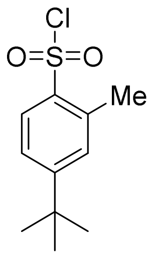

14

TLC (PE: EtOAc, 10:1, v/v): Rf=0.5.

^{1}H NMR (500 MHz, $CDCl_3$) δ 7.98 (d, *J* = 9.1 Hz, 1H), 7.42 – 7.38 (m, 2H), 2.78 (s, 3H), 1.35 (s, 9H).

^{13}C NMR (126 MHz, $CDCl_3$) δ 159.6, 140.4, 137.9, 130.6, 128.9, 123.9, 35.4, 31.1, 20.7.

LRMS (EI) for $C_{11}H_{15}ClO_2S$: calculated: 246.05 [M], found 246.05.

HPLC method to determine the stability of iDegs:

Time (min)	%A	%B
0	90	10
1	90	10
3.3	50	50
6	5	95
8	5	95
10	90	10

A: Double distilled (dd) H_2O + 0.1% (v/v) formic acid; B: acetonitrile + 0.1% (v/v) formic acid; flow rate: 0.25 mL/min. Instruments: Velos Pro (Thermo Fisher Scientific, US), Ultimate 3000 HPLC (Thermo Fisher Scientific, US).

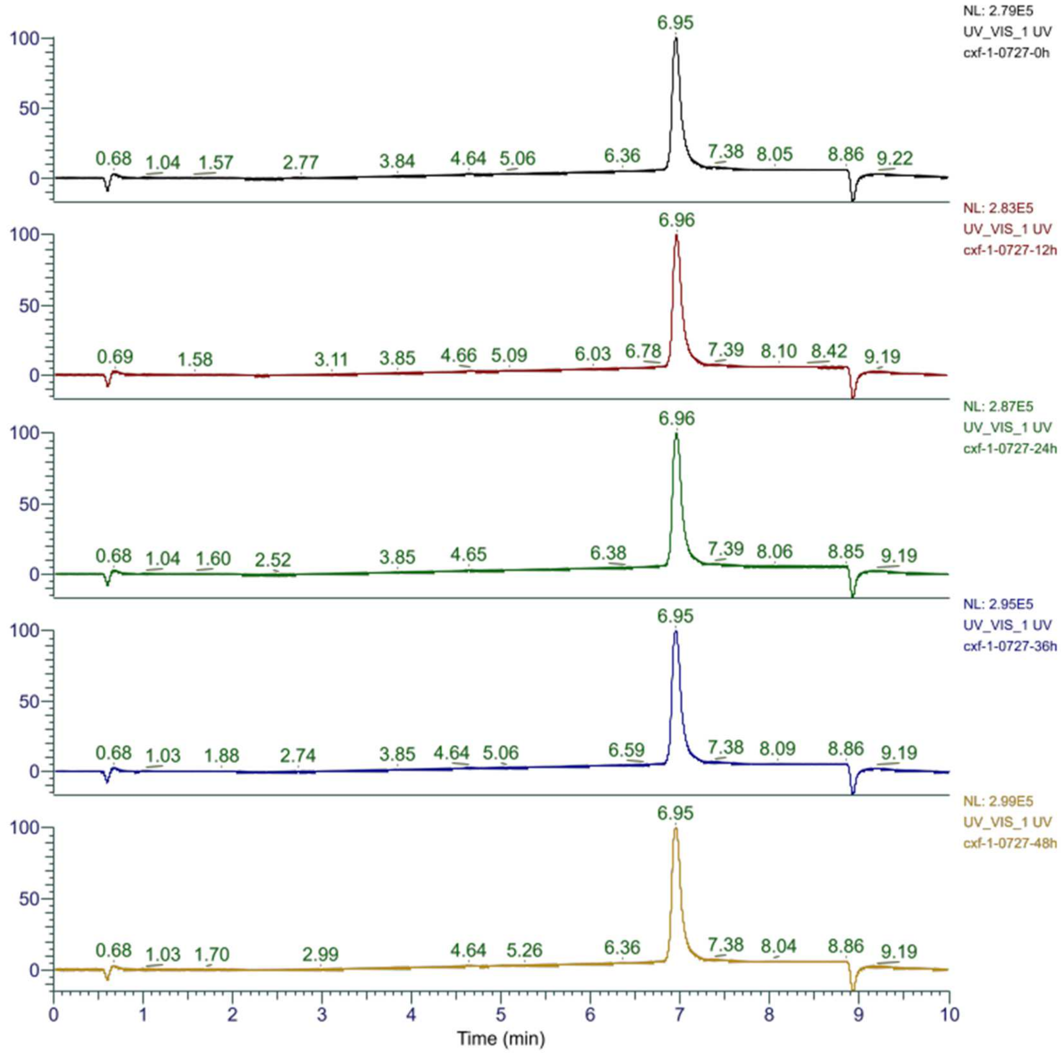

Supplementary Fig. 5. Stability of iDeg-6. A 100 µM solution of iDeg-6 was incubated in

H_2O/MeCN (v/v = 1/1) for up to 48 h at 37 °C and subsequently subjected to HPLC-ESI-MS analysis.

NMR-spectra

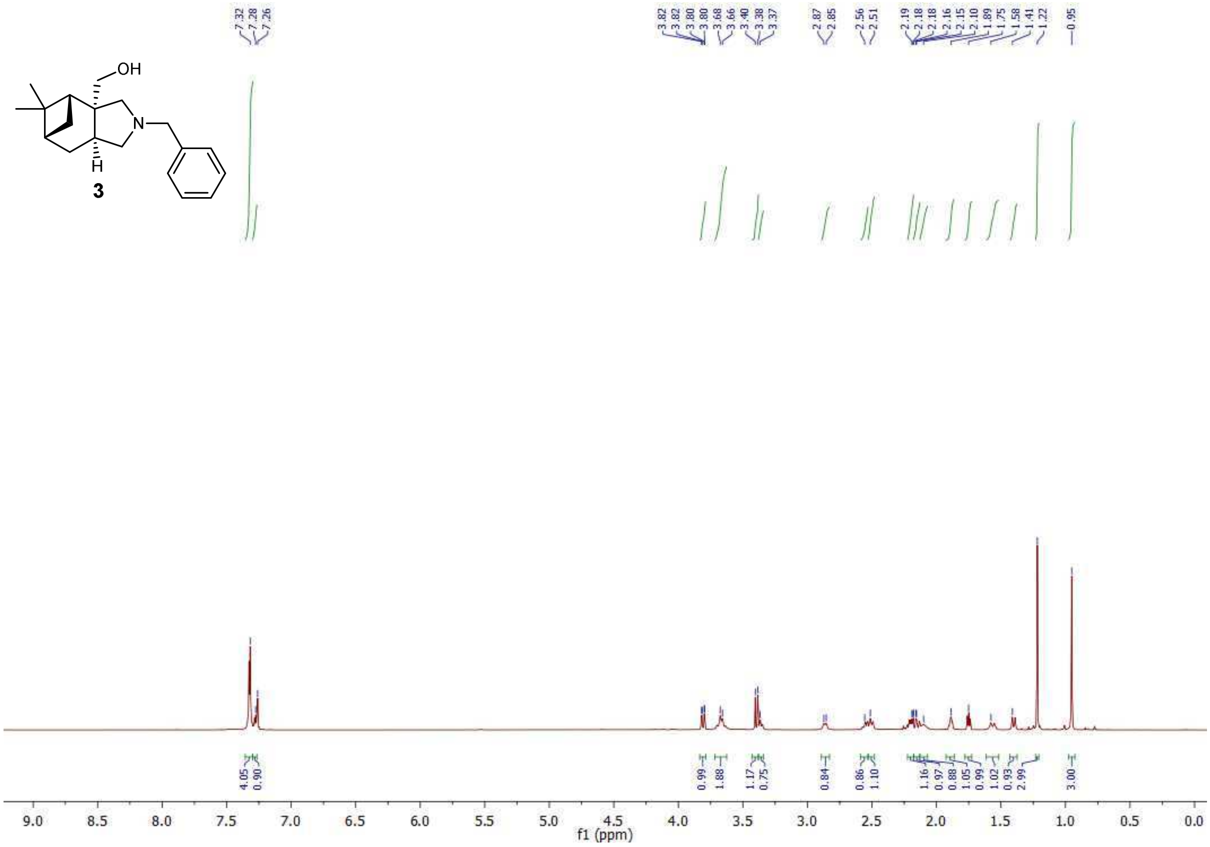

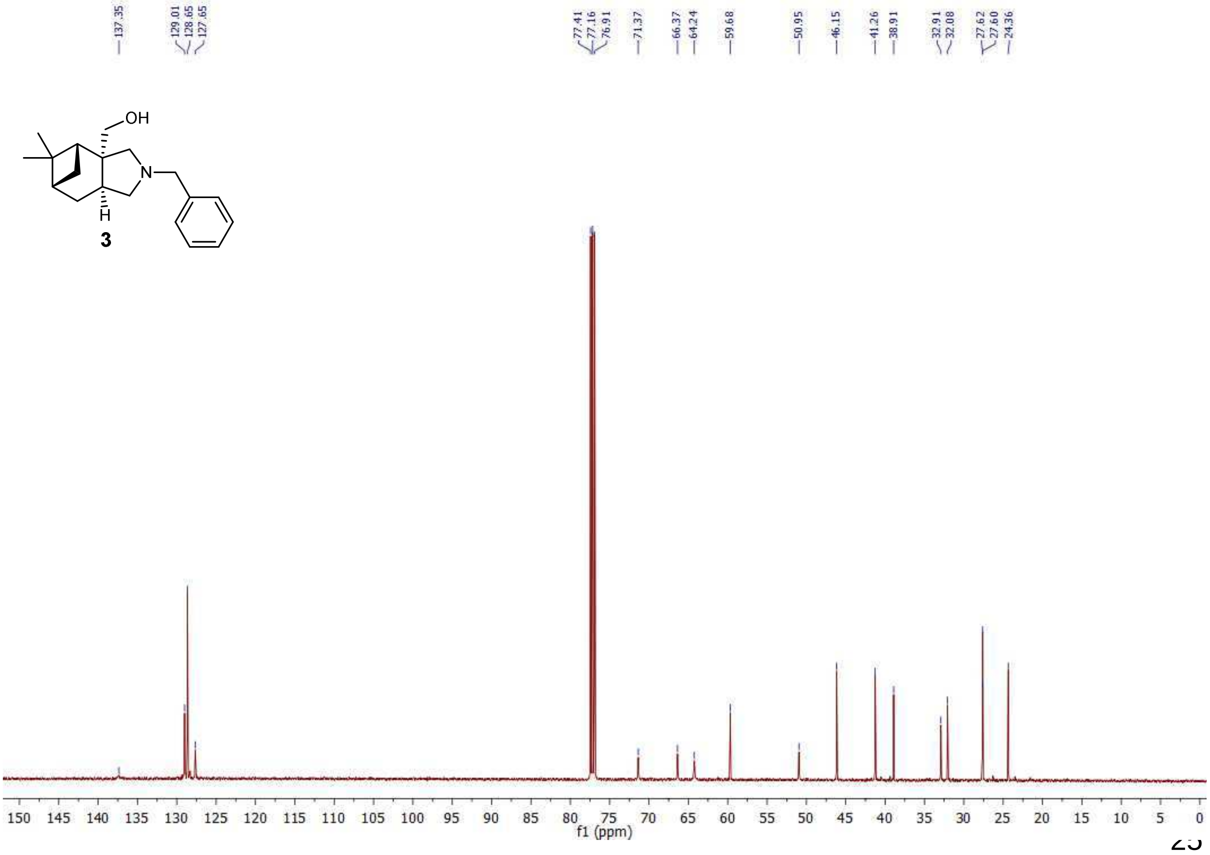

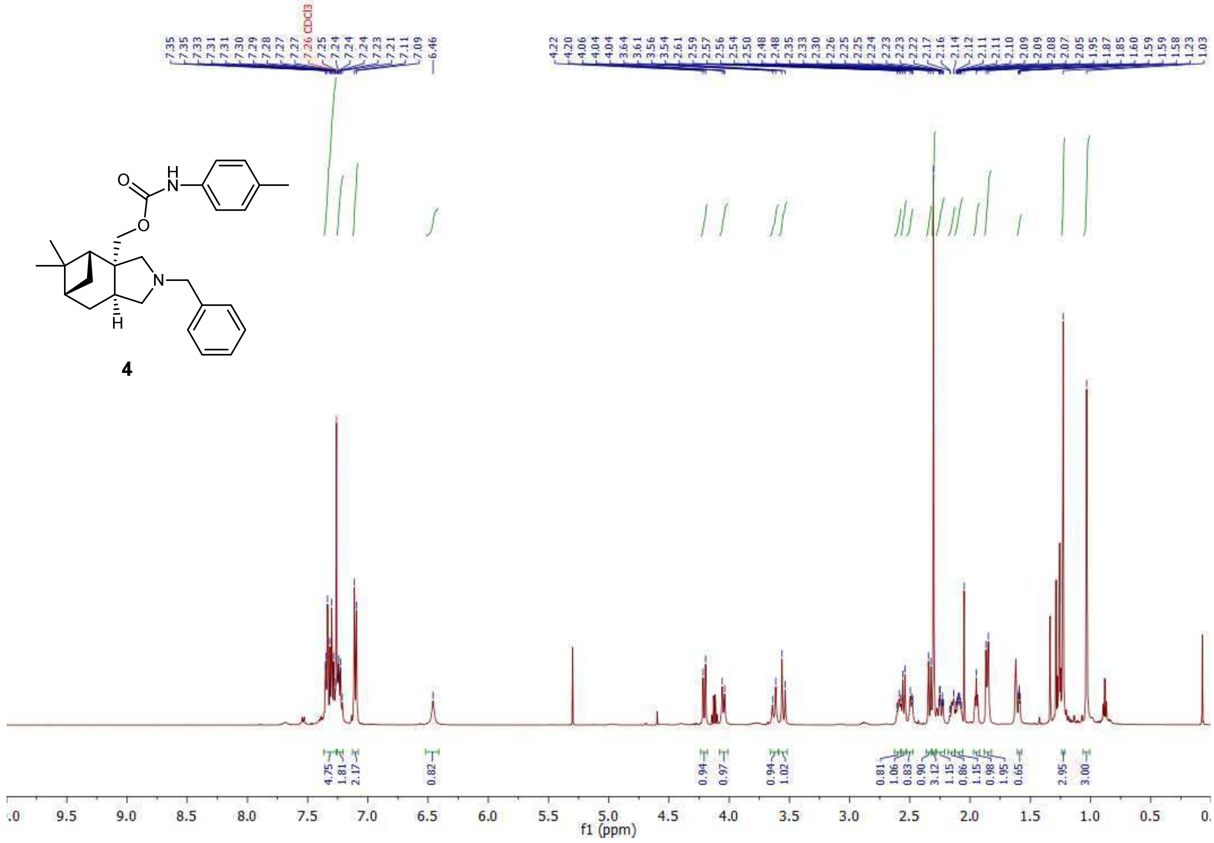

4

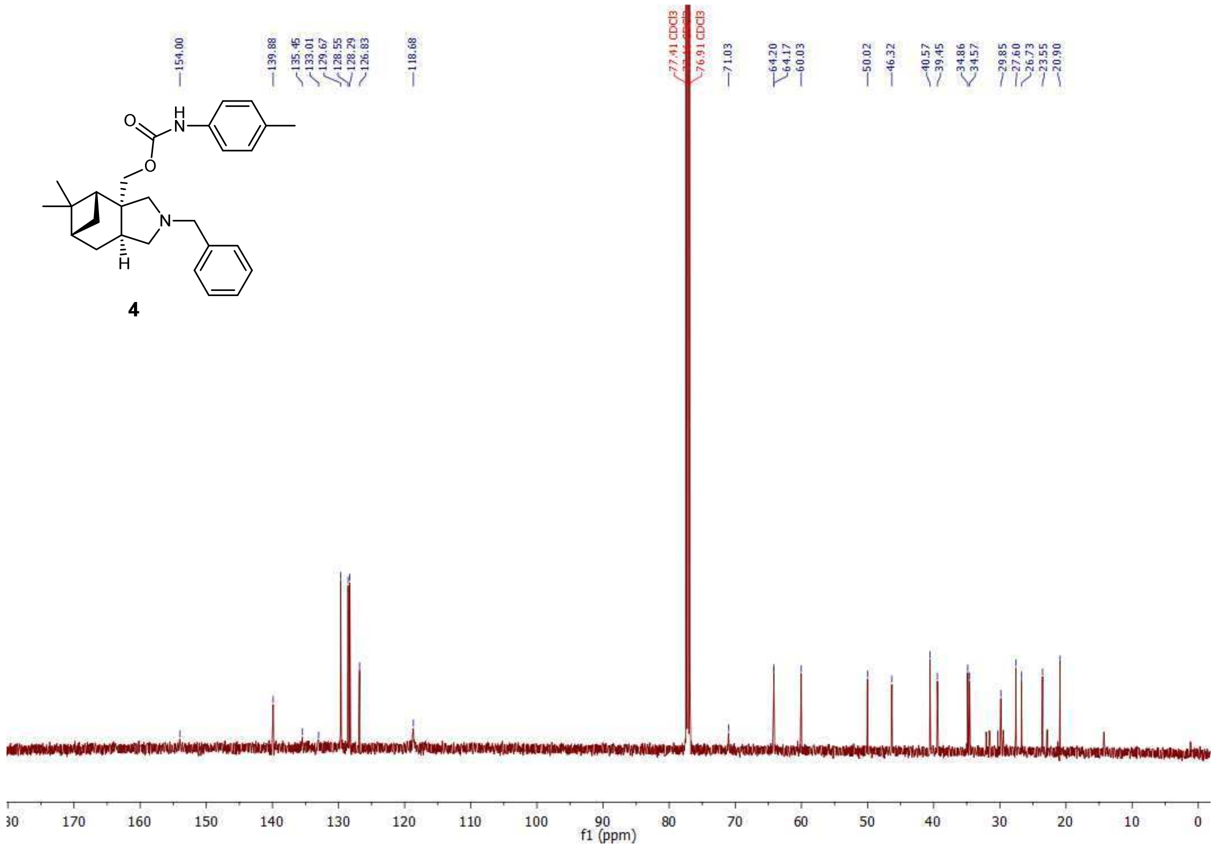

4

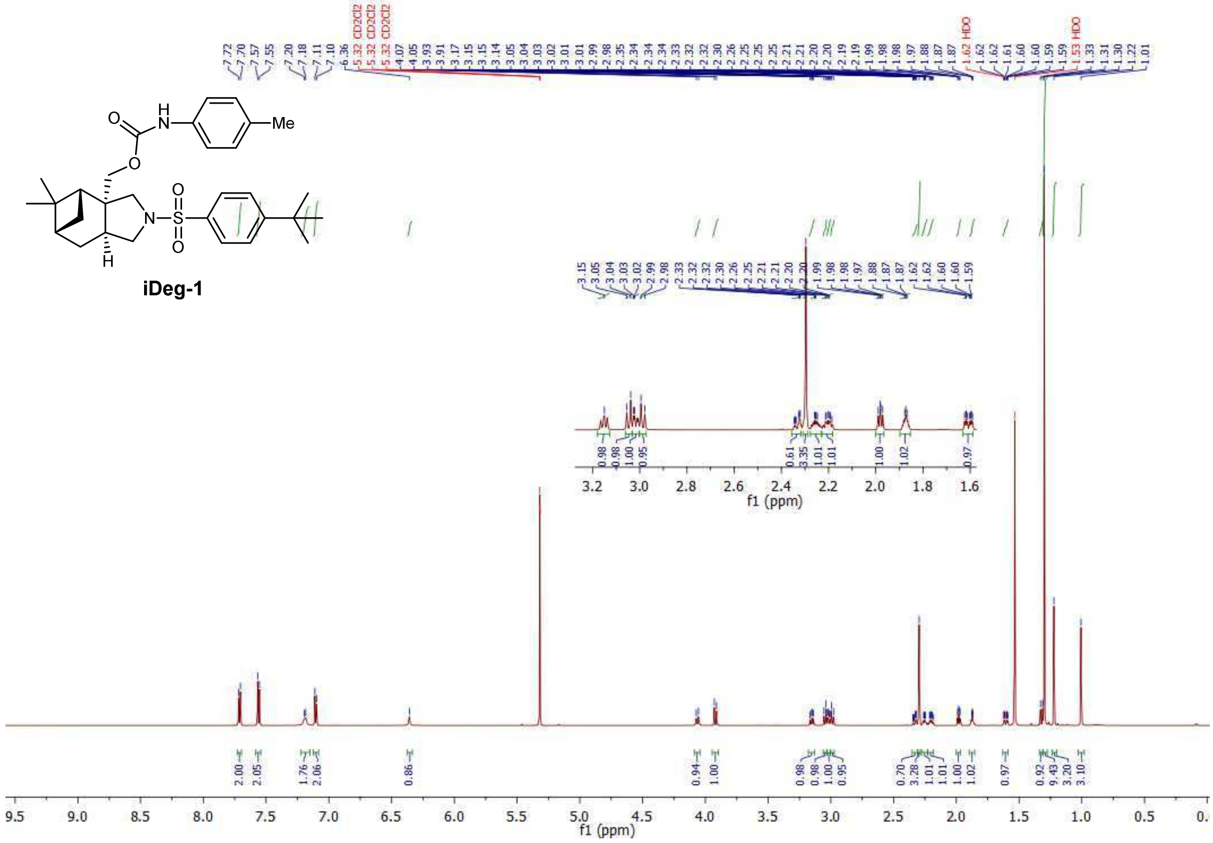

iDeg-1

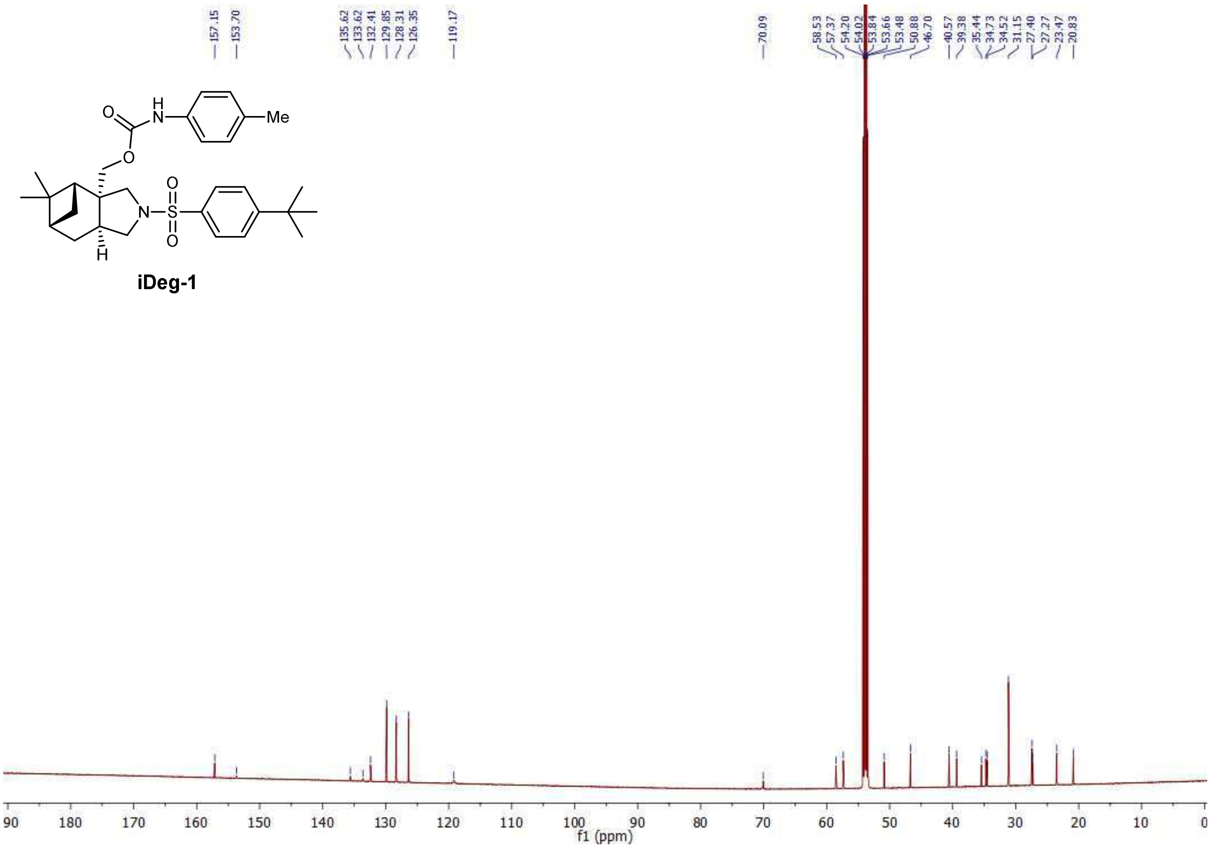

iDeg-1

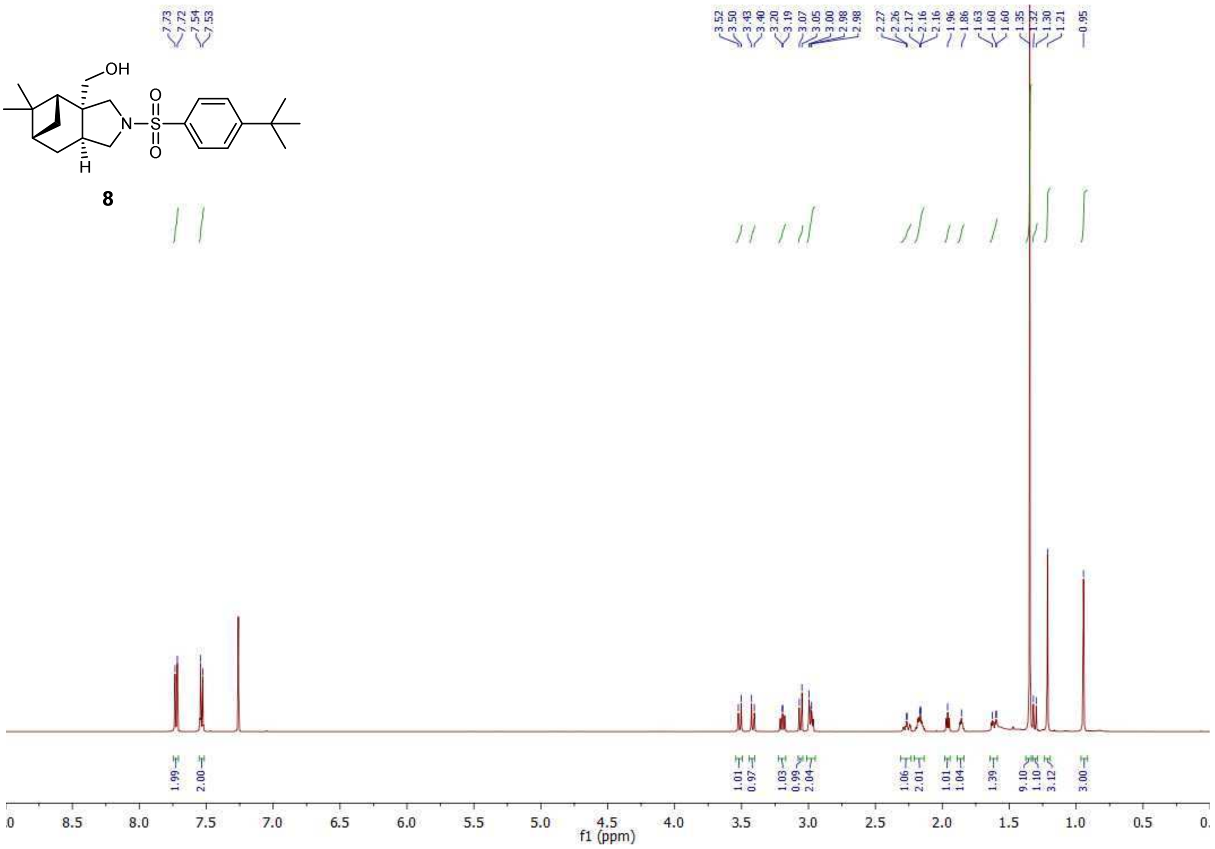

OH
O
S
N
O
H
7.73
7.72
7.54
7.53
3.52
3.50
3.43
3.40
3.20
3.19
3.07
3.05
3.00
2.98
2.98
2.27
2.26
2.17
2.16
2.16
1.96
1.86
1.63
1.60
1.60
1.35
1.32
1.30
1.21
0.95
1.99
2.00
1.01
0.97
1.03
0.99
2.04
1.06
2.01
1.01
1.04
1.39
9.10
1.10
3.12
3.00
f1 (ppm)

8

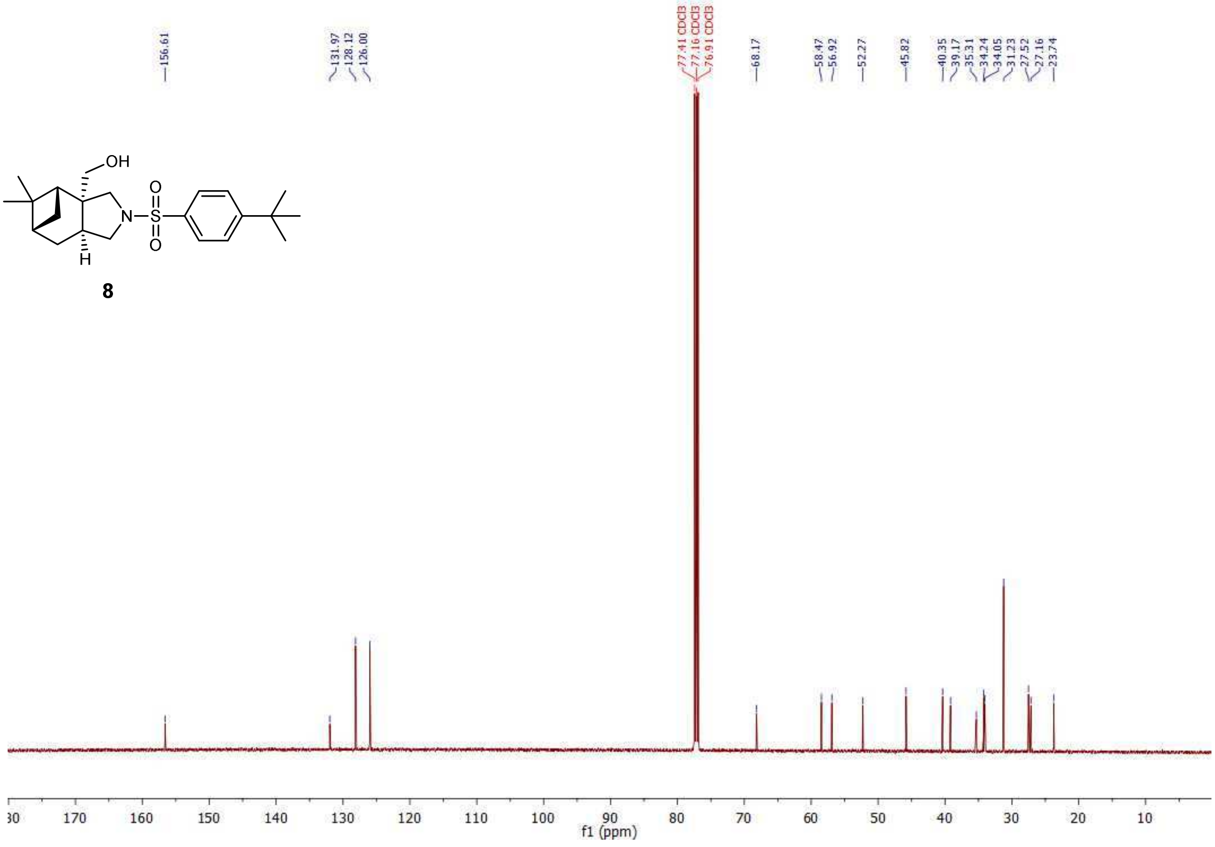

OH
O
S
N
O
H
156.61
131.97
128.12
126.00
77.41 CDCl3
77.16 CDCl3
76.91 CDCl3
68.17
58.47
56.92
52.27
45.82
40.35
39.17
35.31
34.24
34.05
31.23
27.52
27.16
23.74
f1 (ppm)

8

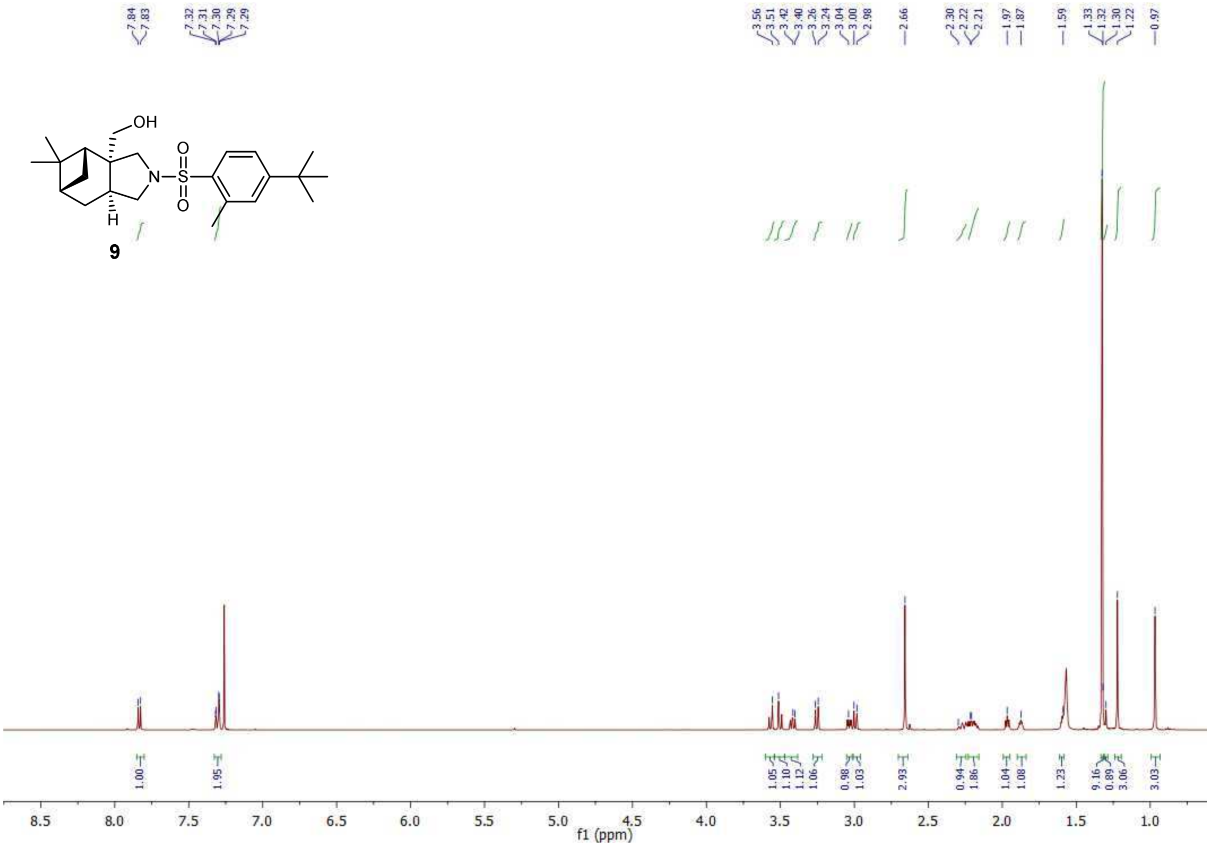
7.84
7.83
7.32
7.31
7.30
7.29
7.29
3.56
3.51
3.42
3.40
3.26
3.24
3.04
3.00
2.98
2.66
2.30
2.22
2.21
1.97
1.87
1.59
1.33
1.32
1.30
1.22
0.97
OH
N
S
O
O
H
9
1.00
1.95
1.05
1.10
1.12
1.06
0.98
1.03
2.93
0.94
1.86
1.04
1.08
1.23
9.16
0.89
3.06
3.03
8.5
8.0
7.5
7.0
6.5
6.0
5.5
5.0
4.5
4.0
3.5
3.0
2.5
2.0
1.5
1.0
f1 (ppm)

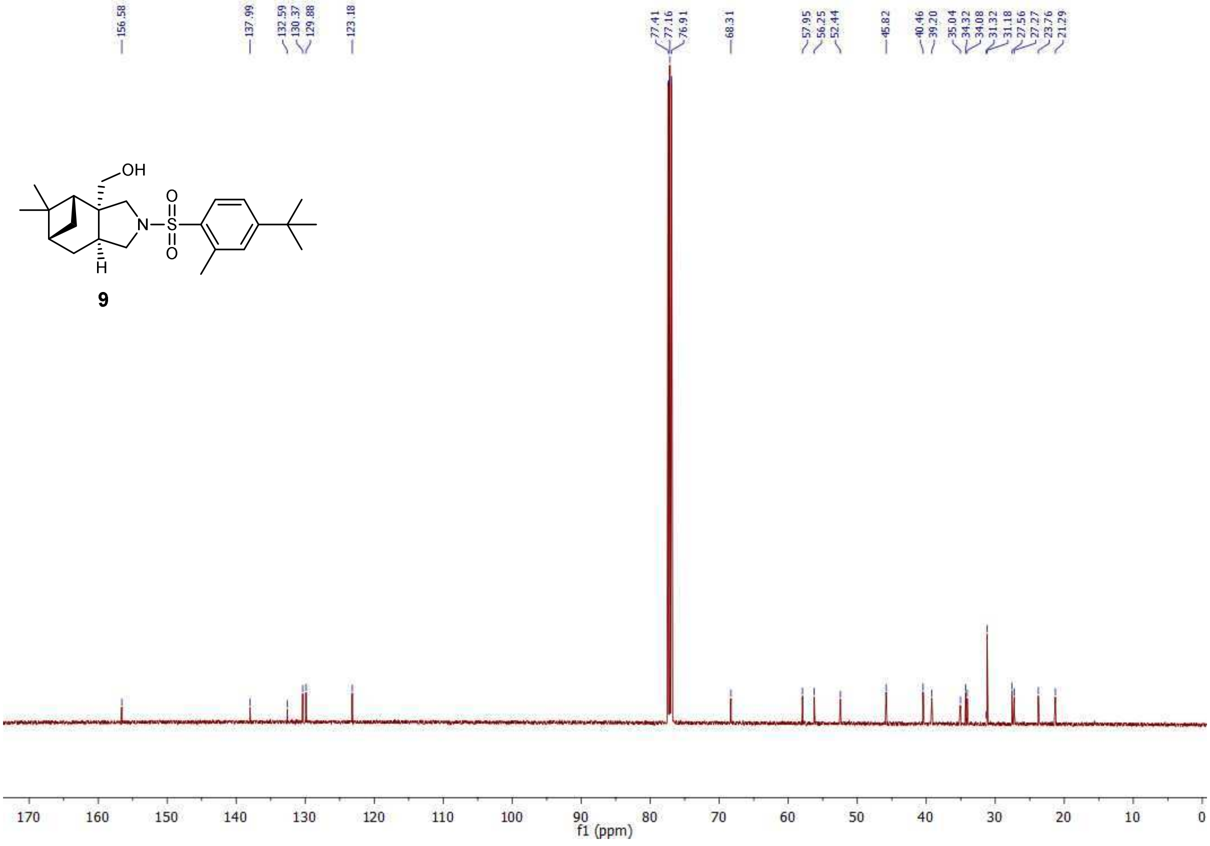
156.58
137.99
132.59
130.37
129.88
123.18
77.41
77.16
76.91
68.31
57.95
56.25
52.44
45.82
40.46
39.20
35.04
34.32
34.08
31.32
31.18
27.56
27.27
23.76
21.29
OH
N
S
O
O
H
9
170
160
150
140
130
120
110
100
90
80
70
60
50
40
30
20
10
0
f1 (ppm)

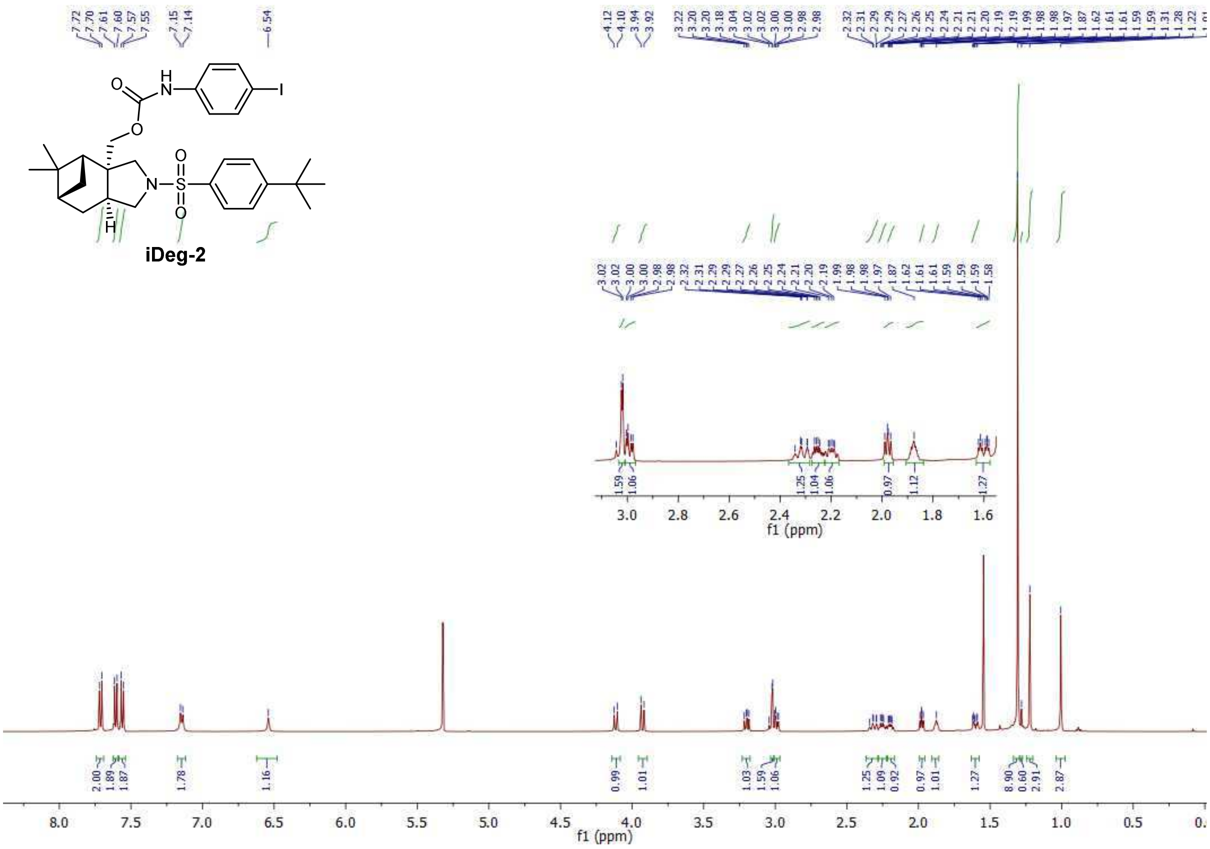
iDeg-2
f1 (ppm)

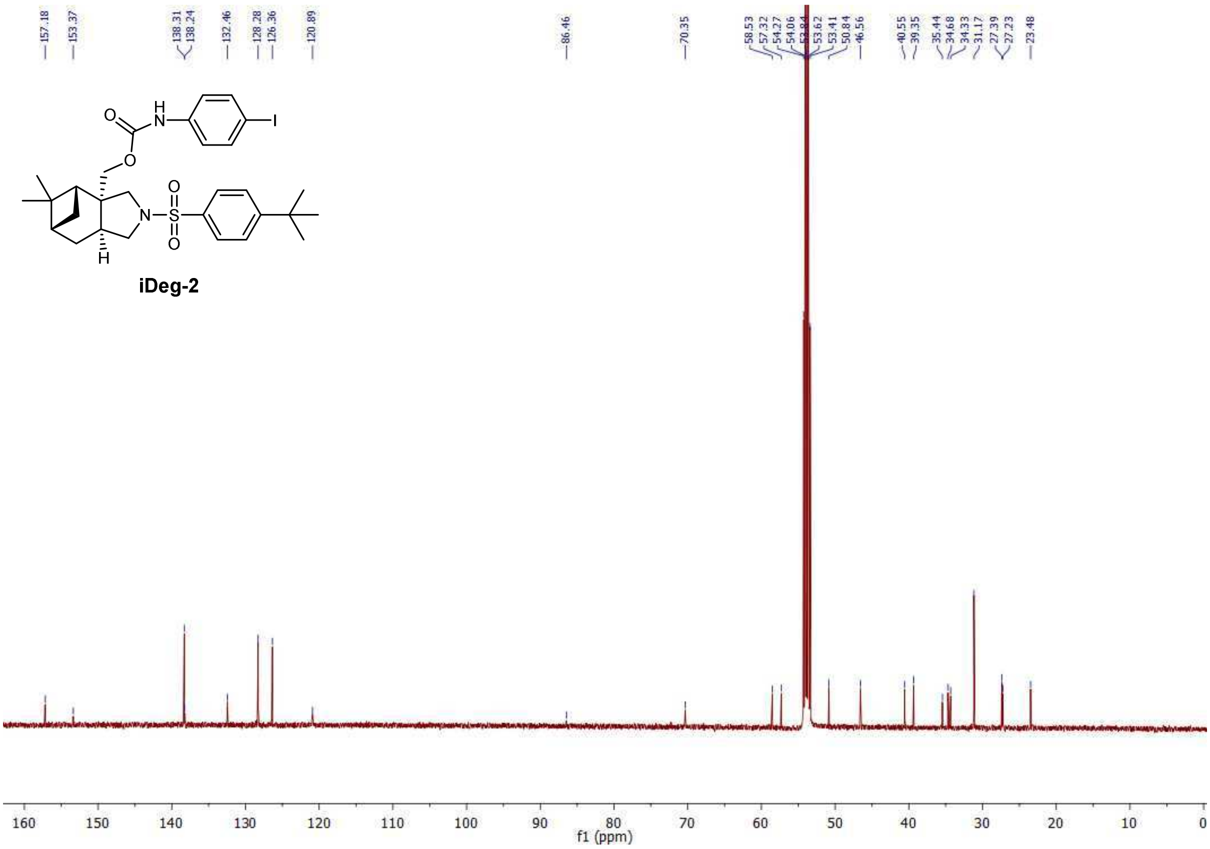
iDeg-2
f1 (ppm)

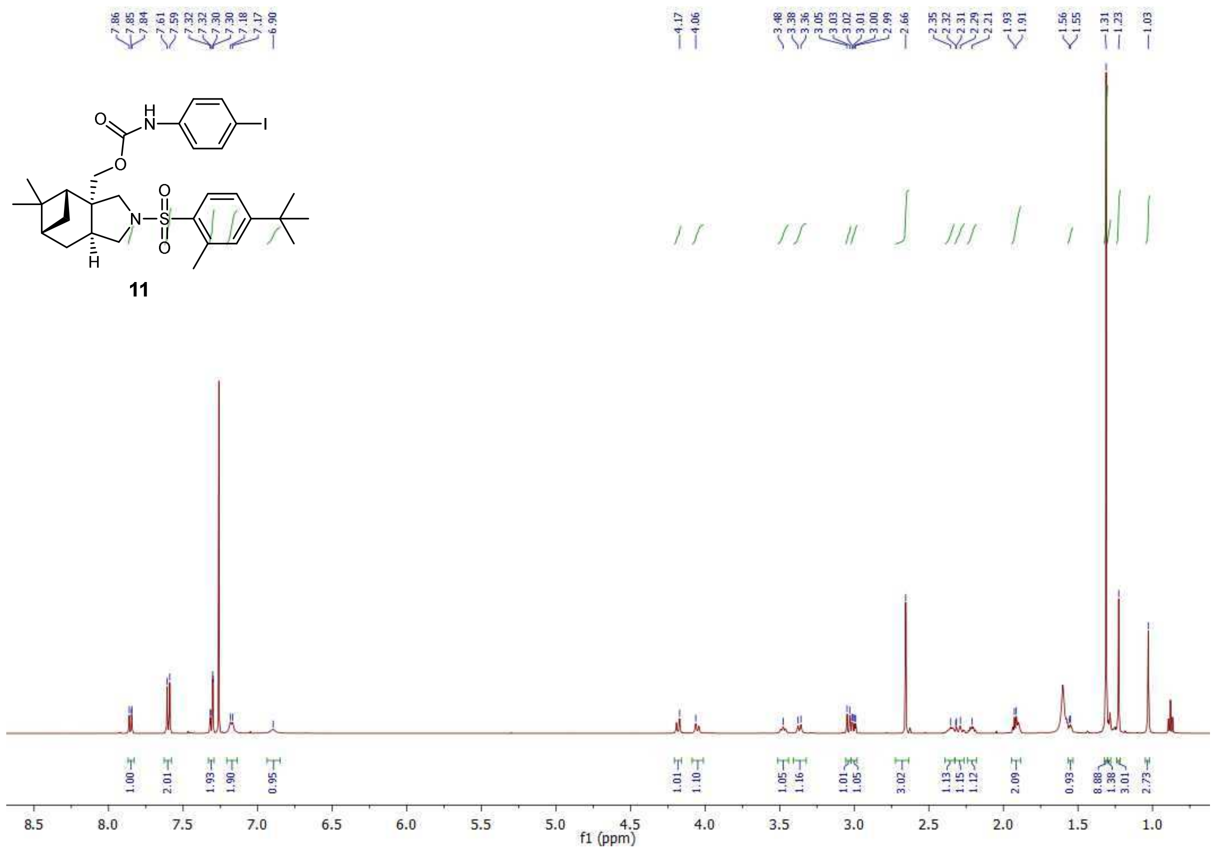

7.86 7.85 7.84 7.61 7.59 7.32 7.32 7.30 7.30 7.18 7.17 6.90
4.17 4.06
3.48 3.38 3.36 3.05 3.03 3.02 3.01 3.00 2.99 2.66
2.35 2.32 2.31 2.29 2.21 1.93 1.91
1.56 1.55 1.31 1.23 1.03
1.00 2.01 1.93 1.90 0.95 1.01 1.10 1.05 1.16 1.01 1.05 3.02 1.13 1.15 1.12 2.09 0.93 8.88 1.38 3.01 2.73
f1 (ppm)

11

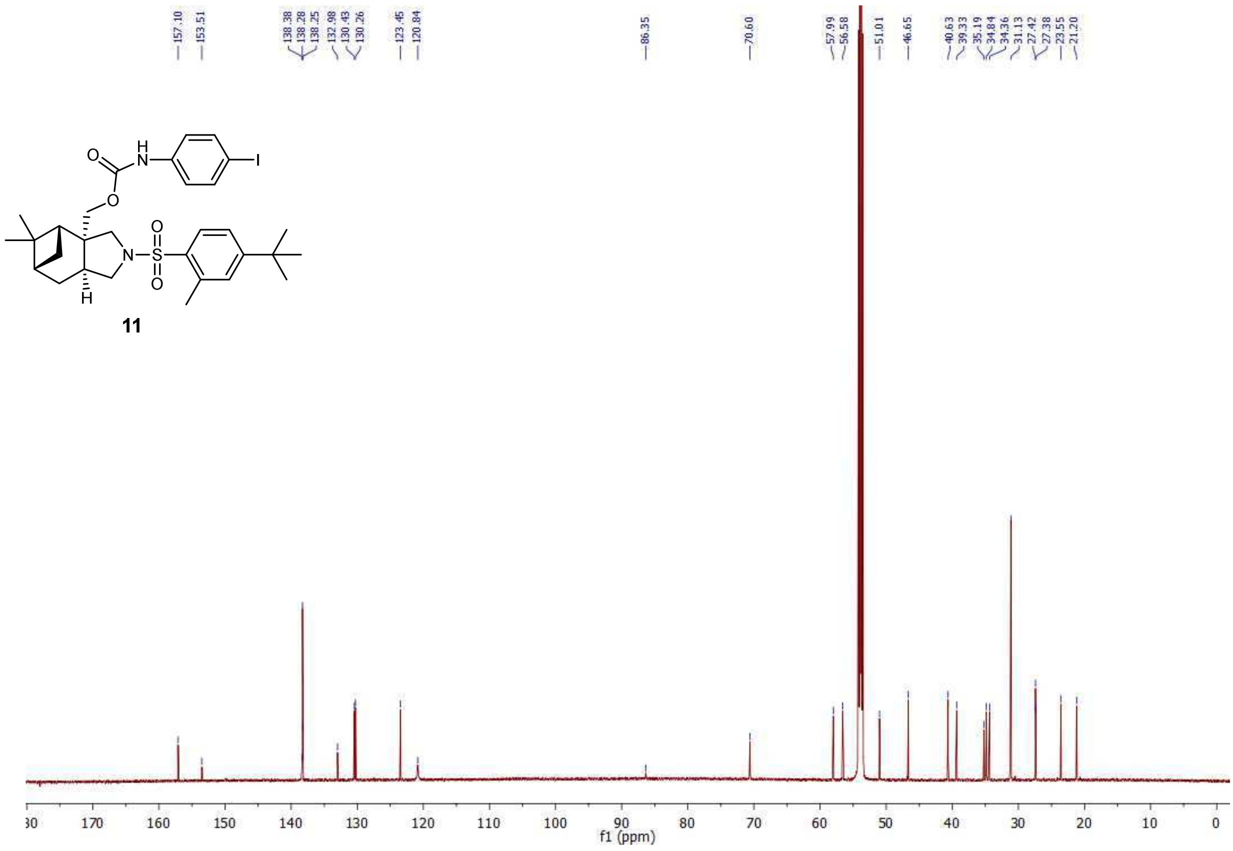

157.10 153.51 138.38 138.28 138.25 132.98 130.43 130.26 123.45 120.84 86.35 70.60 57.99 56.58 51.01 46.65 40.63 39.33 35.19 34.84 34.36 31.13 27.42 27.38 23.55 21.20
f1 (ppm)

11

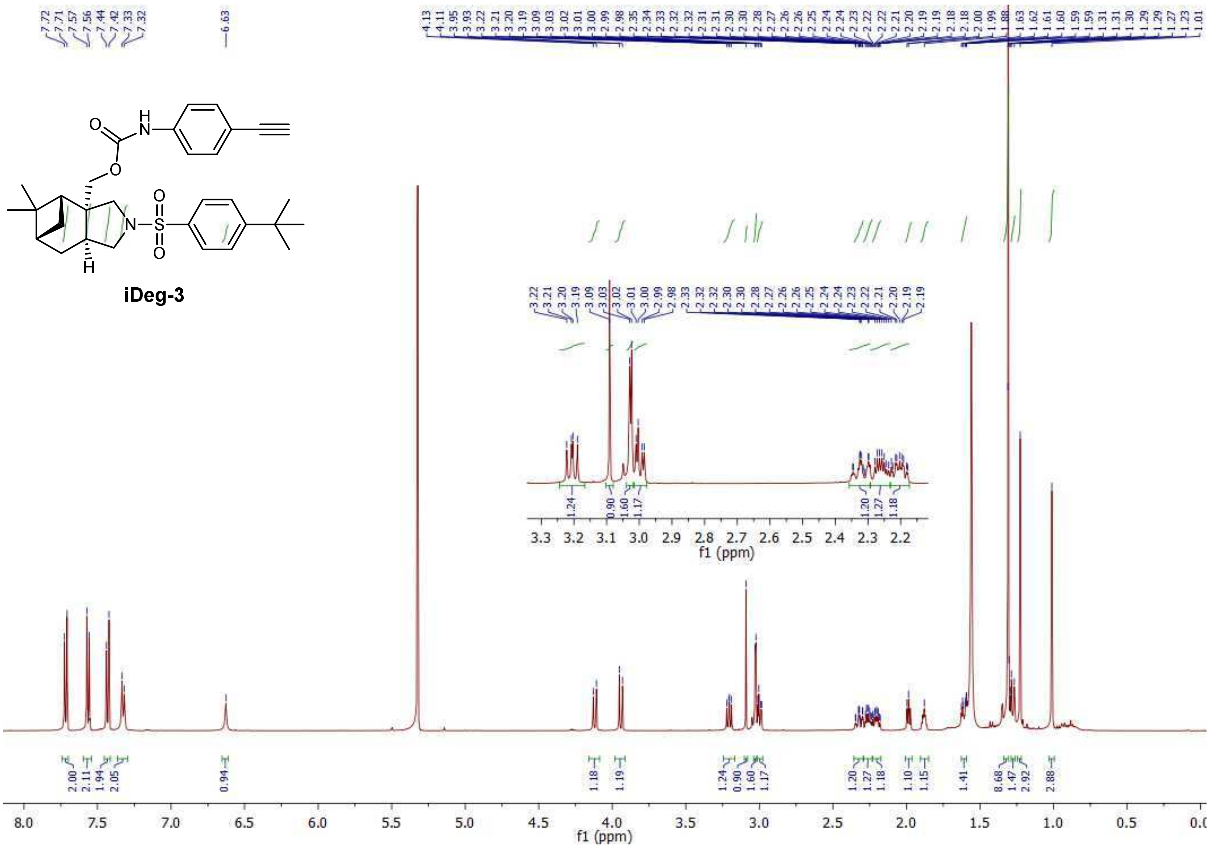

7.72 7.71 7.57 7.56 7.44 7.42 7.33 7.32
6.63
f1 (ppm)

iDeg-3

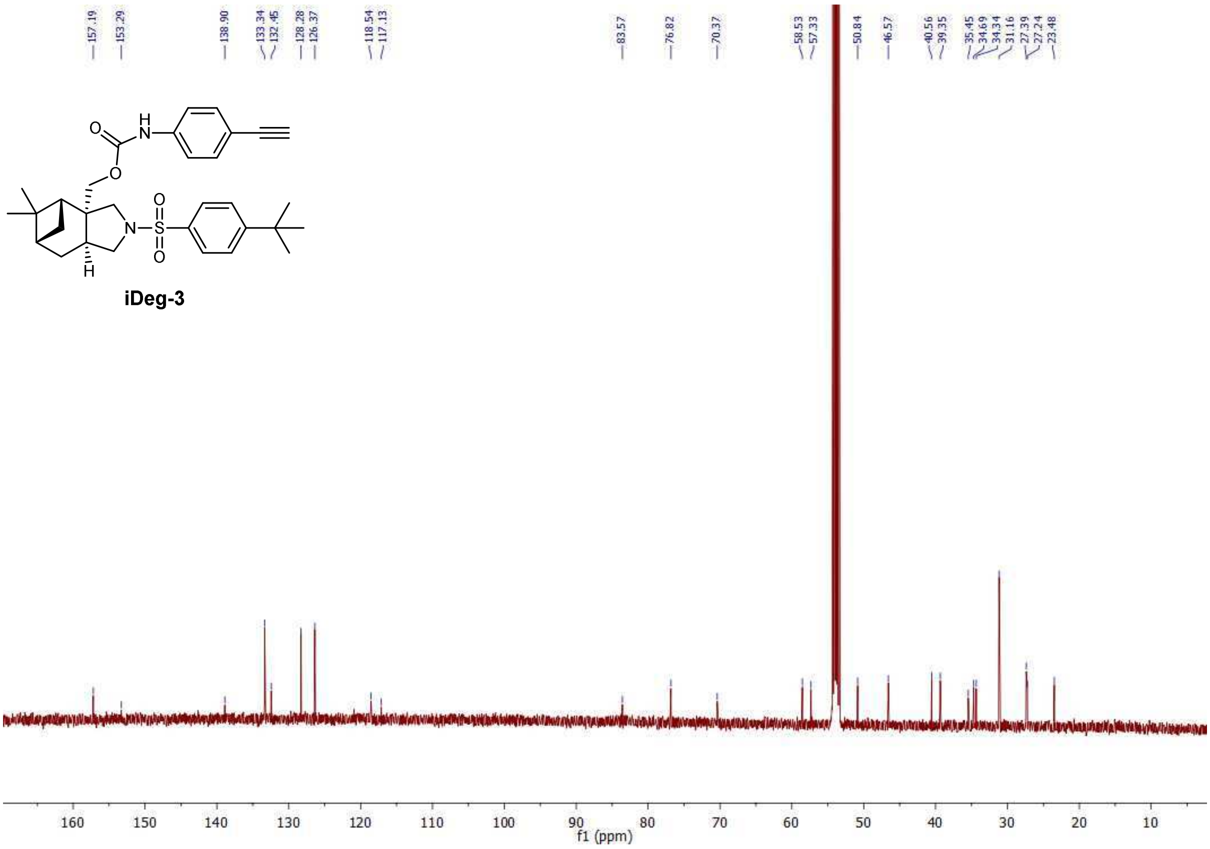

157.19 153.29 138.90 133.34 132.45 128.28 126.37 118.54 117.13 83.57 76.82 70.37 58.53 57.33 50.84 46.57 40.56 39.35 35.45 34.69 34.34 31.16 27.39 27.24 23.48
f1 (ppm)

iDeg-3

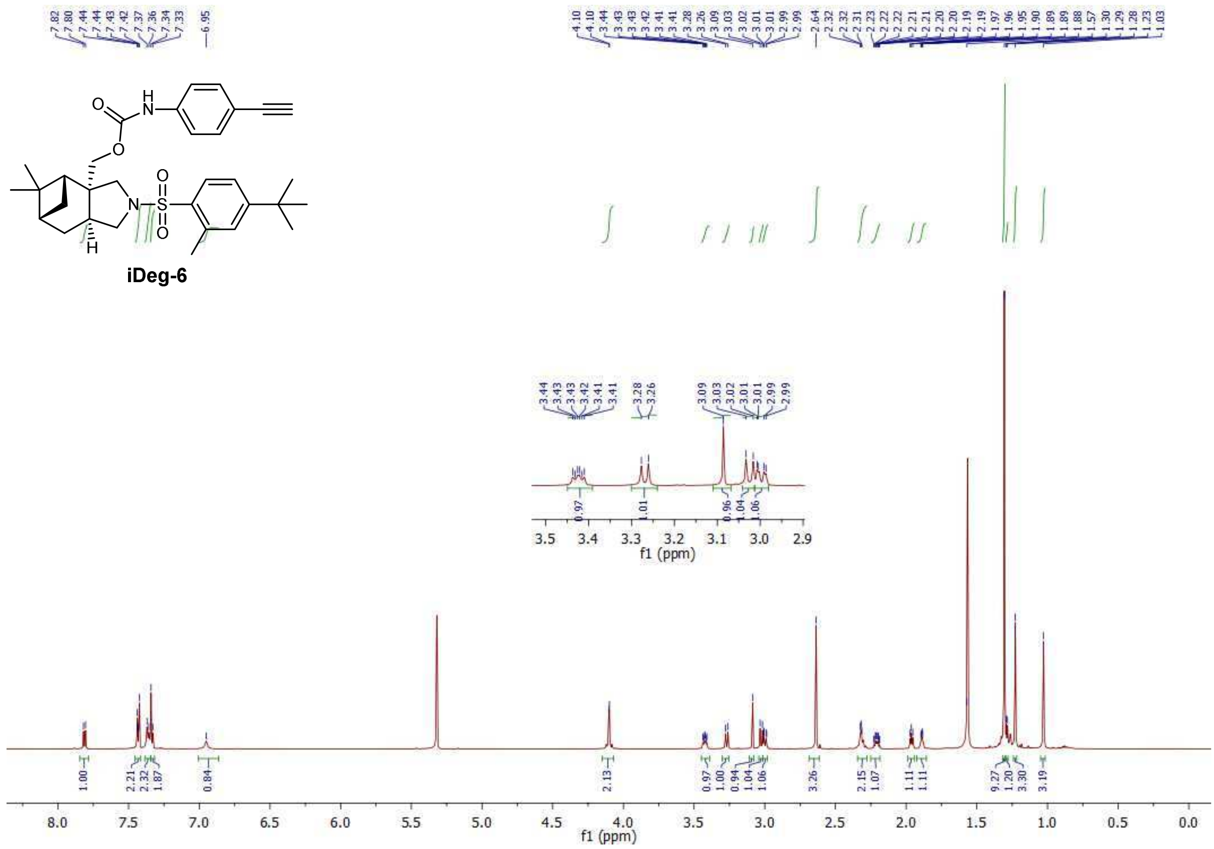

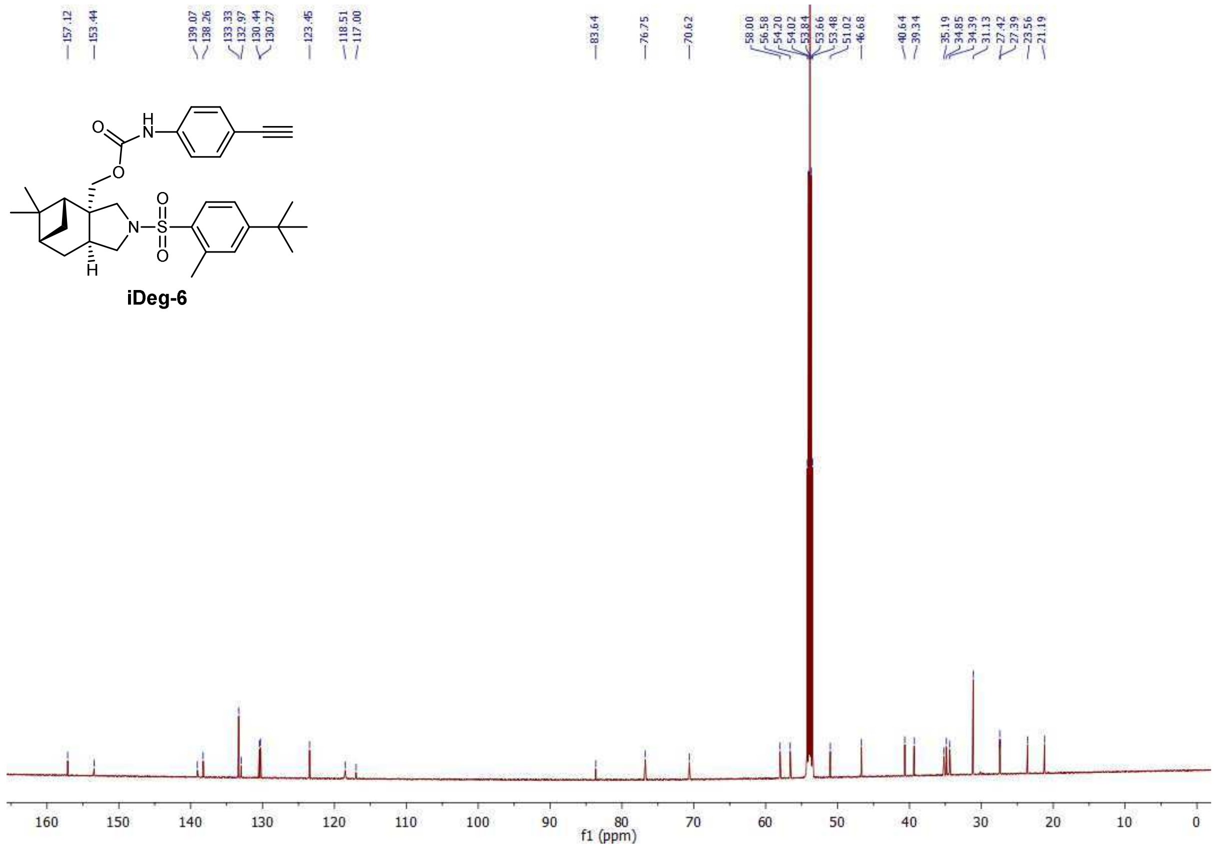

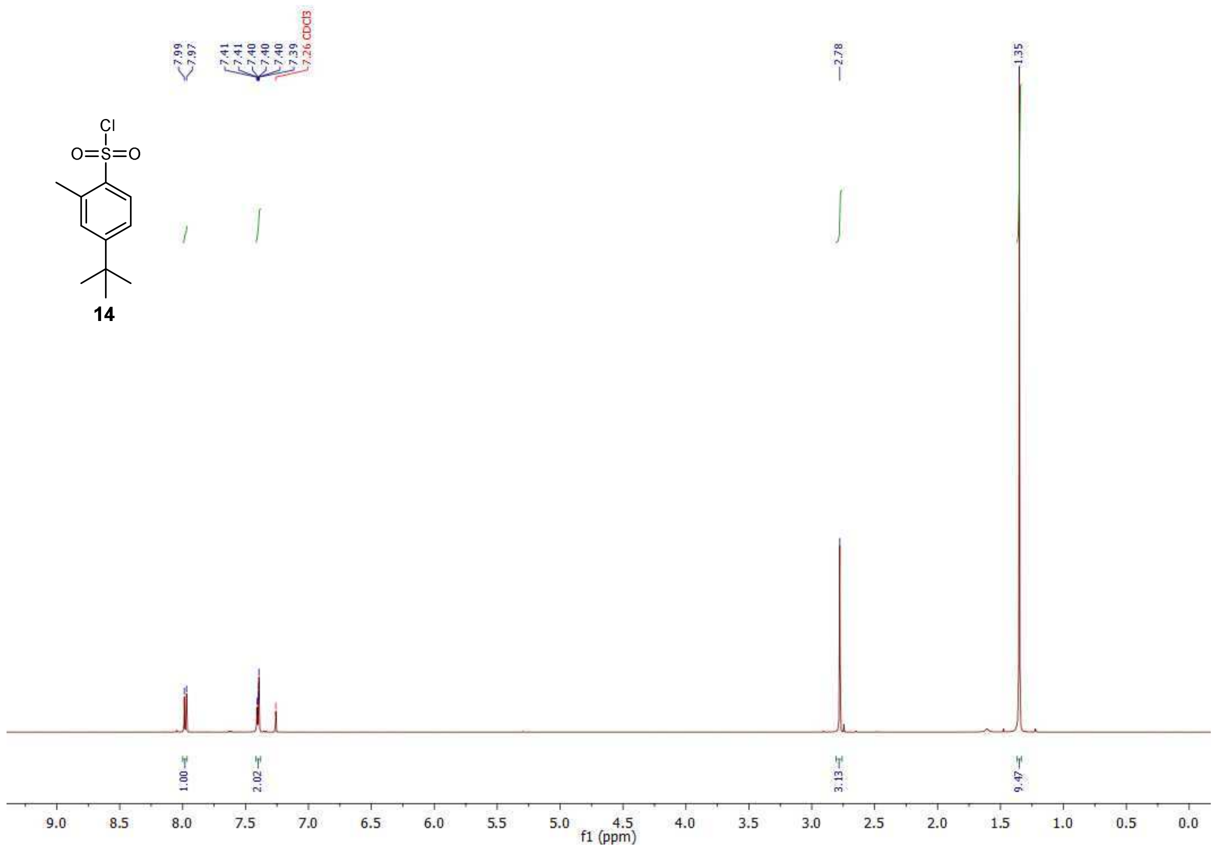

7.99
7.97
7.41
7.41
7.40
7.40
7.40
7.39
7.26 CDCl3
2.78
1.35
1.00
2.02
3.13
9.47
9.0 8.5 8.0 7.5 7.0 6.5 6.0 5.5 5.0 4.5 4.0 3.5 3.0 2.5 2.0 1.5 1.0 0.5 0.0
f1 (ppm)

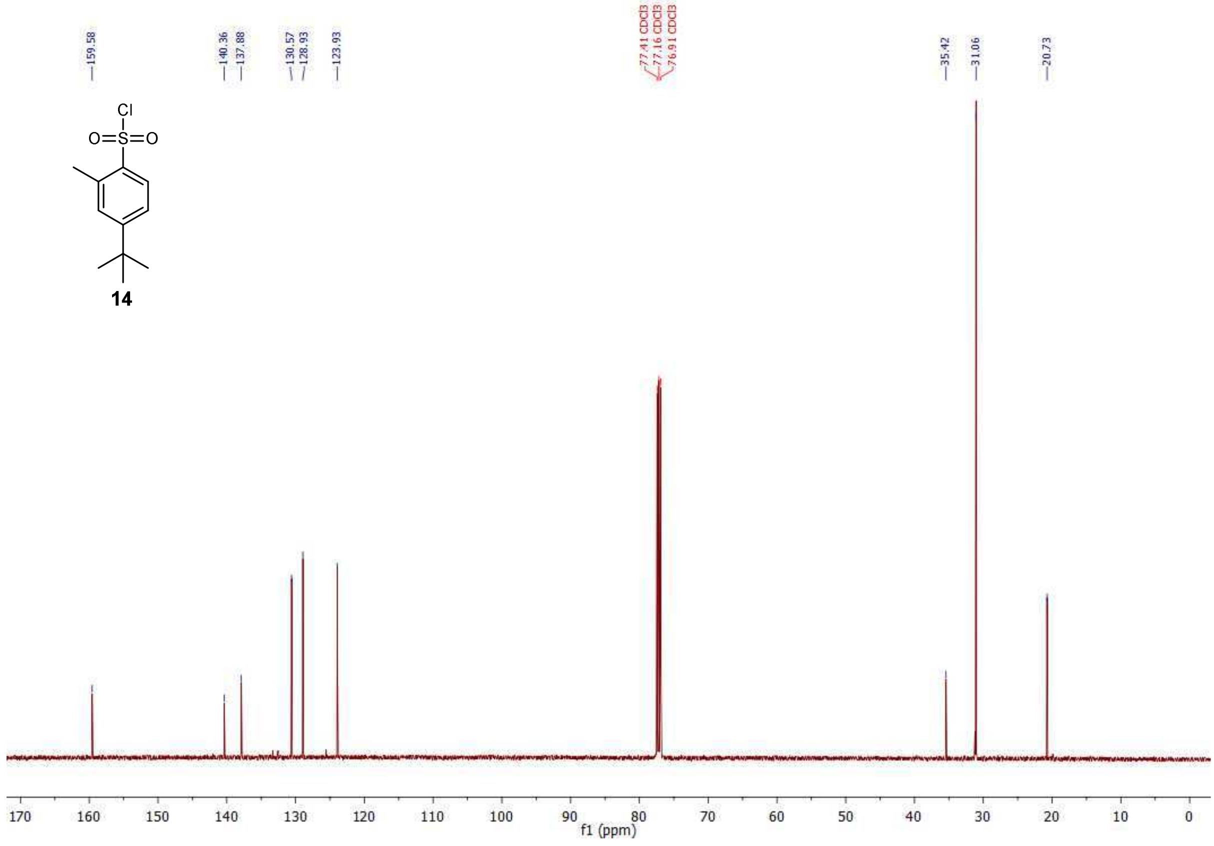

159.58
140.36
137.88
130.57
128.93
123.93
77.41 CDCl3
77.16 CDCl3
76.91 CDCl3
35.42
31.06
20.73
170 160 150 140 130 120 110 100 90 80 70 60 50 40 30 20 10 0
f1 (ppm)

Source data

Uncropped gel for Supplementary Fig. S2a

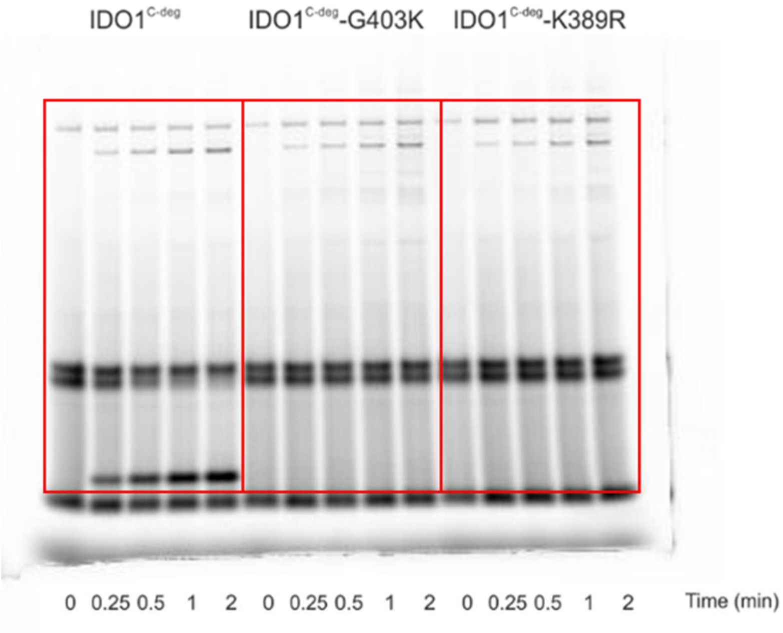

Uncropped gel for Supplementary Fig. S4

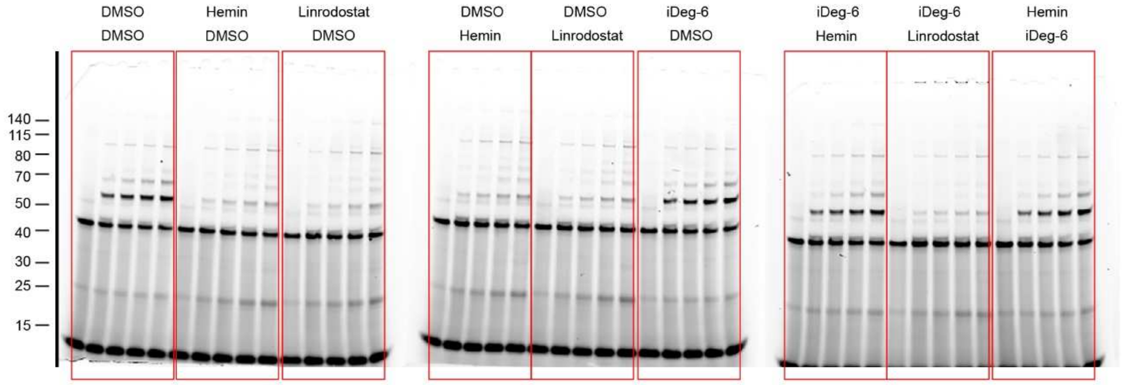

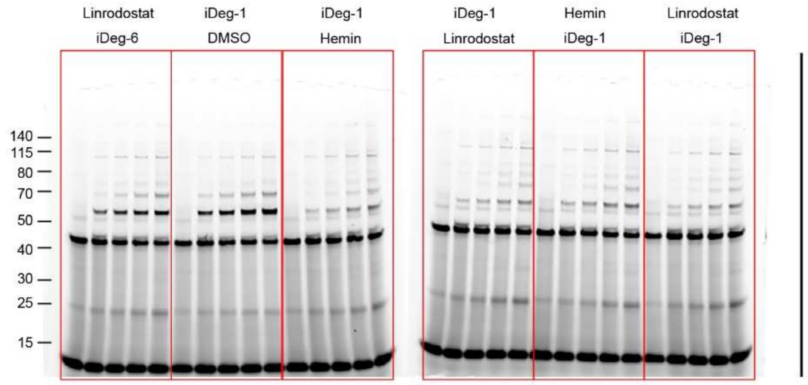

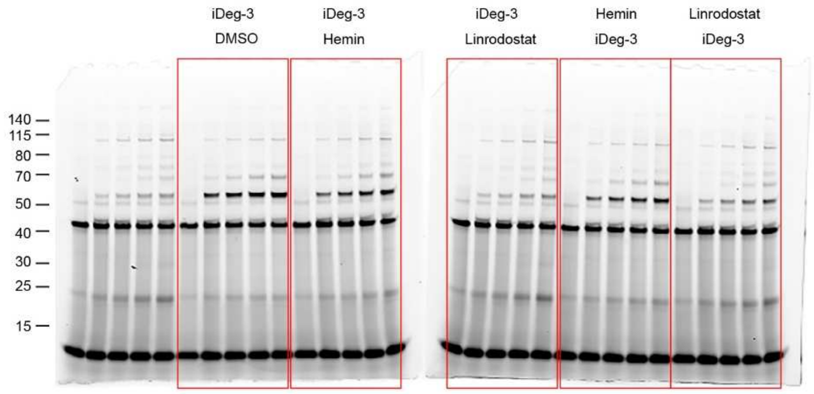

References

1 Sheffler, D. J., Nedelcovych, M. T., Williams, R., Turner, S. C., Duerk, B. B., Robbins, M. R. *et al.* Novel GlyT1 inhibitor chemotypes by scaffold hopping. Part 2: Development of a [3.3.0]-based series and other piperidine bioisosteres (vol 24, pg 1062, 2014). *Bioorganic & Medicinal Chemistry Letters* **27**, 2079-2079 (2017).

2 Mutti, S., Daubie, C., Decalogne, F., Fournier, R. & Rossi, P. Enantiospecific synthesis of RPR 107880: A new non peptide substance P antagonist. *Tetrahedron Lett* **37**, 3125-3128 (1996).

3 Zhong, L. J., Xiong, Z. Q., Ouyang, X. H., Li, Y., Song, R. J., Sun, Q. *et al.* Intermolecular 1,2-Difunctionalization of Alkenes Enabled by Fluoroamide-Directed Remote Benzyl C(sp)-H Functionalization. *Journal of the American Chemical Society* **144**, 339-348 (2022).
