# Supplementary material for: Monovalent pseudo-natural products supercharge degradation of IDO1 by its native E3 KLHDC3
Source: Nat Chem. 2026 Jan 7;18(3):585–96. doi: 10.1038/s41557-025-02021-5 (PMC12962974; doi:10.1038/s41557-025-02021-5)
Supplement: Supplementary file 2 — Reporting Summary [file 41557_2025_2021_MOESM2_ESM.pdf]

Corresponding author(s): Herbert Waldmann

Last updated by author(s): Dec 19, 2024

Reporting Summary

Nature Portfolio wishes to improve the reproducibility of the work that we publish. This form provides structure for consistency and transparency in reporting. For further information on Nature Portfolio policies, see our Editorial Policies and the Editorial Policy Checklist.

Statistics

For all statistical analyses, confirm that the following items are present in the figure legend, table legend, main text, or Methods section.

n/a	Confirmed	
☐	☒	The exact sample size (n) for each experimental group/condition, given as a discrete number and unit of measurement
☐	☒	A statement on whether measurements were taken from distinct samples or whether the same sample was measured repeatedly
☐	☒	The statistical test(s) used AND whether they are one- or two-sided<br>*Only common tests should be described solely by name; describe more complex techniques in the Methods section.*
☒	☐	A description of all covariates tested
☒	☐	A description of any assumptions or corrections, such as tests of normality and adjustment for multiple comparisons
☐	☒	A full description of the statistical parameters including central tendency (e.g. means) or other basic estimates (e.g. regression coefficient) AND variation (e.g. standard deviation) or associated estimates of uncertainty (e.g. confidence intervals)
☐	☒	For null hypothesis testing, the test statistic (e.g. F, t, r) with confidence intervals, effect sizes, degrees of freedom and P value noted<br>*Give P values as exact values whenever suitable.*
☒	☐	For Bayesian analysis, information on the choice of priors and Markov chain Monte Carlo settings
☒	☐	For hierarchical and complex designs, identification of the appropriate level for tests and full reporting of outcomes
☒	☐	Estimates of effect sizes (e.g. Cohen's d, Pearson's r), indicating how they were calculated

Our web collection on statistics for biologists contains articles on many of the points above.

Software and code

Policy information about availability of computer code

Data collection	Flow cytometry: BD LSRFortessa using BD FACSDiva software (v9.0), BD FACSAria Fusion using BD FACSDiva software (v8.0.2) or CytoFLEX SRT Benchtop Cell Sorter using CytExpert SRT-Software (v1.1.0.10007).<br>Immunoblotting: ChemiDocTM MP system (Bio-Rad) operated on Image Lab (v2.4.0.03).<br>NGS: NovaSeq 6000, Illumina (https://www.illumina.com/).<br>Mass spectrometry: UltiMateTM 3000 RSLCnano system (Thermo Fisher Scientific, Germany) online coupled to a Q Exactive™ HF Hybrid Quadrupole-Orbitrap Mass Spectrometer equipped with a nanospray source (Nanospray Flex Ion Source, Thermo Fisher Scientific).<br>nanoDSF: Prometheus Panta device (NanoTemper® Technologies).<br>RT-qPCR: CFX96 Real-Time PCR Detection System (Bio-Rad).<br>Reporter gene assa, Kyn assay, UV(Vis analysis: on Spark® multimode microplate plate reader (Tecan).<br>Live-cell analysis: IncuCyte S3 system (Sartorius)<br>Microscopy: Axiovert 200M (Zeiss) andMetaXpress software for image analysis.<br>ITC: MicroCal AutoITC200 (Malvern) using MicroCal PEAQ software.
Data analysis	Flow Cytometry Analysis: Flowjo (v10.6.2)<br>FACS-based CRISPR screens: pipelines for sgRNA quantification and statistical analysis are available on Github (https://github.com/ZuberLab/crispr-processnf/ tree/566f6d46bbcc2a3f49f51bbc96b9820f408ec4a3 and https://github.com/ZuberLab/crisprmageck- nf/tree/c75a90f670698bfa78bfd8be786d6e5d6d4fc455). Packages: fastx-toolkit (v0.0.14), Bowtie2 (v2.4.5), featureCounts (v2.0.1), MAGeCK (v0.5.9).<br>Immunoblot quantification: Image Lab (v6.0)

Data compiling and processing: Microsoft Excel 2016, GraphPad Prism (6.0, 9.2.0)
Mass Spectrometry: MaxQuant (v.1.6.17.0)
ITC: MicroCal PEAQ software
X-ray analysis: XDS, XSCALE, Phaser11 (Phenix suite12), phenix.refinePHENIX, COOT13, PyMOL Molecular Graphics System (2.5.4, Schrödinger, LLC), LigPlot Plus14.

For manuscripts utilizing custom algorithms or software that are central to the research but not yet described in published literature, software must be made available to editors and reviewers. We strongly encourage code deposition in a community repository (e.g. GitHub). See the Nature Portfolio guidelines for submitting code & software for further information.

Data

Policy information about availability of data

All manuscripts must include a data availability statement. This statement should provide the following information, where applicable:

- Accession codes, unique identifiers, or web links for publicly available datasets
- A description of any restrictions on data availability
- For clinical datasets or third party data, please ensure that the statement adheres to our policy

The proteomics data have been deposited in MassIVE with the accession code MSV000094270, PXD050474 (global proteome profiling) and MSV000094271, PXD050475 (IDO1 immunoprecipitation).
The crystal structures of IDO1 with iDeg-1 and iDeg-2 were deposited in the PBD with the accession numbers 9RIS and 9FOH.

Research involving human participants, their data, or biological material

Policy information about studies with human participants or human data. See also policy information about sex, gender (identity/presentation), and sexual orientation and race, ethnicity and racism.

Reporting on sex and gender	N/A
Reporting on race, ethnicity, or other socially relevant groupings	N/A
Population characteristics	N/A
Recruitment	N/A
Ethics oversight	N/A

Note that full information on the approval of the study protocol must also be provided in the manuscript.

Field-specific reporting

Please select the one below that is the best fit for your research. If you are not sure, read the appropriate sections before making your selection.

☒ Life sciences ☐ Behavioural & social sciences ☐ Ecological, evolutionary & environmental sciences

For a reference copy of the document with all sections, see nature.com/documents/nr-reporting-summary-flat.pdf

Life sciences study design

All studies must disclose on these points even when the disclosure is negative.

Sample size	Sample size was not predetermined. Sample size was selected based on previous experience and is indicated in each figure caption.
Data exclusions	No data were excluded
Replication	All experiments were performed in three biological replicates unless otherwise stated. The number of biological replicates is specified in the respective figure legends.
Randomization	No randomization was performed as is this is not standard for biochemical and in vitro studies. Internal controls were included for comparison.
Blinding	No blinding was performed as no subjective measurements were done.

Reporting for specific materials, systems and methods

We require information from authors about some types of materials, experimental systems and methods used in many studies. Here, indicate whether each material, system or method listed is relevant to your study. If you are not sure if a list item applies to your research, read the appropriate section before selecting a response.

Materials & experimental systems

n/a	Involved in the study
☐	☒ Antibodies
☐	☒ Eukaryotic cell lines
☒	☐ Palaeontology and archaeology
☒	☐ Animals and other organisms
☒	☐ Clinical data
☒	☐ Dual use research of concern
☒	☐ Plants

Methods

n/a	Involved in the study
☒	☐ ChIP-seq
☐	☒ Flow cytometry
☒	☐ MRI-based neuroimaging

Antibodies

Antibodies used

Immunoblotting: anti-IDO1 (1:2500, 14-9750-80, ThermoFisher), anti-vinculin (1:10,000, V9131, ThermoFisher), anti-KLHDC3 (1:000, HPA030131, Sigma-Aldrich), anti-rabbit HRP (7074, Cell Signaling Technology), anti-IDO1 (1:5000, ab211017, abcam), anti-β-actin (1:20,000, ab8227, abcam), anti-tubulin (1:2500, ab18251, abcam). IRDye-conjugated secondary antibodies were obtained from LI-COR Biosciences. Anti-DOCK-8 (1:500, 11622-1-AP) and anti- RHOBTB3 (1:800, 13945-1-AP) were obtained from Proteintech.
Flow cytometry and FACS: APC anti-mouse CD90.1/Thy-1.1 antibody (1:400, no. 202526, BioLegend) and Human TruStain FcXTM Fc Receptor Blocking Solution (1:400, no. 422302, BioLegend). IRDye 800CW goat anti-mouse IgG secondary antibody (1:5000, #926-32210), IRDye 680CW goat anti-rabbit IgG secondary antibody (1:5000, 926-68071) and IRDye 680RD donkey anti-mouse (1:5000, 926-68072) were obtained from LI-COR Biosciences.

Validation

Antibodies were validated by induction of overexpression (IDO1, Results section), knockout (KLHDC3, Results section) or protein degradation analysis (IDO1, Results section). Antibodies were validated by their manufacturers and their use is validated in numerous publications as exemplified by citeab.com.

Eukaryotic cell lines

Policy information about cell lines and Sex and Gender in Research

Cell line source(s)

BxPC3 (ACC 760) and HeLa (ACC 57) cells were purchased from DSMZ GmbH (Germany). BT549 (HTB-122), HEK293-T (CRL-3216), SKOV-3 (HTB-77) and U2OS (HTB-96) cells were obtained from ATCC (USA). U2OS KO1 cells were obtained from St. Jude Center for Advanced Genome Engineering (CAGE) (Memphis, USA). KBM7 iCas9 cells were a gift from J. Zuber (IMP, Vienna). Lenti-X 293T lentiviral packaging cells (#632180) were obtained from Clontech.

Authentication

All used cell lines were authenticated by vendors and routinely authenticated via cell morphology.

Mycoplasma contamination

All used cell lines were routinely tested and confirmed negative for mycoplasma contamination.

Commonly misidentified lines
(See ICLAC register)

No commonly misidentified cell lines were used.

Plants

Seed stocks

N/A

Novel plant genotypes

N/A

Authentication

N/A

Flow Cytometry

Plots

Confirm that:

☒ The axis labels state the marker and fluorochrome used (e.g. CD4-FITC).

☒ The axis scales are clearly visible. Include numbers along axes only for bottom left plot of group (a 'group' is an analysis of identical markers).

☒ All plots are contour plots with outliers or pseudocolor plots.

☒ A numerical value for number of cells or percentage (with statistics) is provided.

Methodology

Sample preparation	The detailed generation and sample preparation can be found in the SI Methods. In brief, IDO1 stability reporter cell lines were generated by lentiviral transduction with the respective mCherry_P2A_BFP_IDO1 vectors. Sorted pools were used for experiments. sgRNAs were also introduced by lentiviral delivery and used after selection with G418 and dox induction. sgRNA harboring cells were confirmed before and after selection by surface antigen staining. For the quantitative flow cytometry experiments, cells were directly measured on a BD LSRFortessa. For sorting, cells were stained and fixed as described in the methods and sorted to achieve at least 1000 x sgRNA representation per replicate.<br><br>Flow cytometric data analysis was performed in FlowJo v10.6.2. BFP and mCherry mean fluorescence intensity (MFI) values DO1 stability was calculated as the ratio of BFP to mCherry MFI, and is displayed normalized to the respective control condition as indicated in the figure legends. For the analysis only reporter positive cells were considered.
Instrument	Data acquisition was performed on a BD LSRFortessa (4 laser, 16 detector configuration; BD Bioscience). Cell sorting was performed on a BD FACSAria Fusion (5 lasers, 16 detectors; BD Bioscience) for CRISPR/Cas9 screens or a CytoFLEX SRT (4 lasers, 15 detectors; Beckman Coulter) for cell line generation.
Software	BD FACSDiva software (v8.0.2 and v9.0), Beckman Coulter CytExpert SRT (v 1.1.0.10007), Flowjo (v10.6.2)
Cell population abundance	For the FACS-based CRISPR/Cas9 screens, cells were sorted into HIGH (5% of cells) or LOW (5%), and MID (30%) populations. Fractions were re-analyzed after collection for purity. In the case of > 5% of cross- contamination, samples were discarded before further processing.
Gating strategy	The forward scatter area vs. side scatter area plot was used to separate cell events from debris and dead cells. Forward scatter height vs. forward scatter area and/or side scatter width vs. side scatter height plots were used to separate single cells from aggregates. For cell populations that had not been sorted prior to the experiments (unsorted pools) or sorted pools with residual reporter negative cells, reporter positive cells were further gated in the Pacific Blue-A (BFP) vs PE-TexasRed-A (mCherry) scatter plots. Next, triple positive sgRNA (AF 647-A), iCas9-GFP (FITC-A/AF 488-A) and reporter (PE-TexasRed-A) cells were sorted into the respective LOW, HIGH, and MID populations based on the BFP (BV421-A/Pacific Blue-A) vs mCherry (PE-TexasRed-A) scatter plots. These gates were dynamically adjusted to keep the percentage at 5% for HIGH and LOW and 30% for MID populations.<br><br>A figure exemplifying the gating strategy for all flow cytometry experiments and FACS-based screens in provided in Extended Data Fig.7.

☒ Tick this box to confirm that a figure exemplifying the gating strategy is provided in the Supplementary Information.
