# Supplementary figures and images for: Monovalent pseudo-natural products supercharge degradation of IDO1 by its native E3 KLHDC3
Source: Nat Chem. 2026 Jan 7;18(3):585–96. doi: 10.1038/s41557-025-02021-5 (PMC12962974; doi:10.1038/s41557-025-02021-5)

Figure 1c

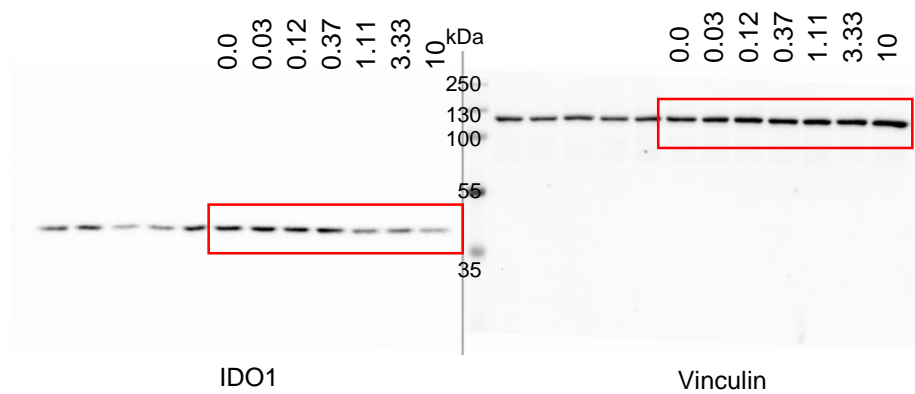

Figure 1e

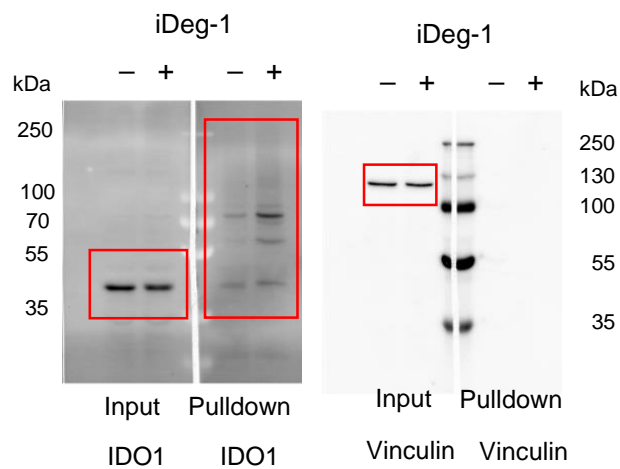

Figure 1f

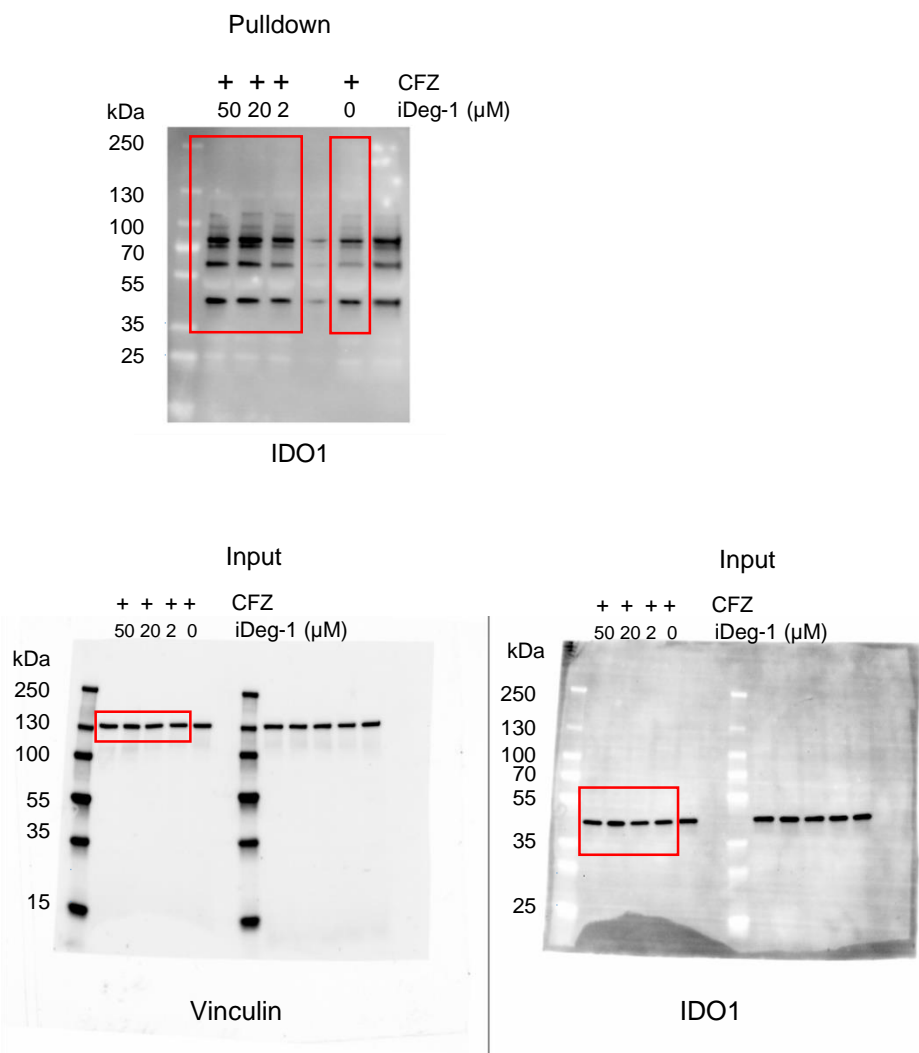

Figure 1g

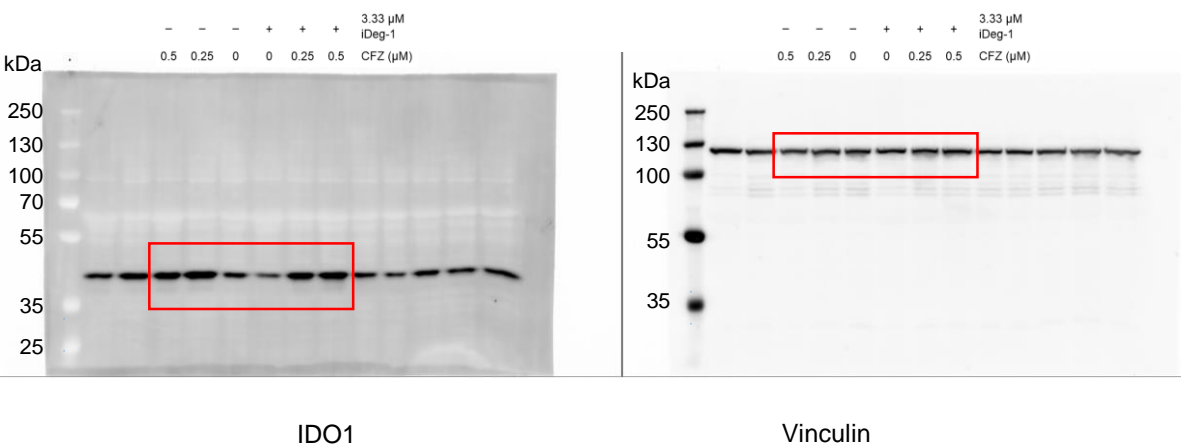

Supplement: Supplementary file 6 — Unprocessed western blots. [file 41557_2025_2021_MOESM6_ESM.pdf]

Figure 2a

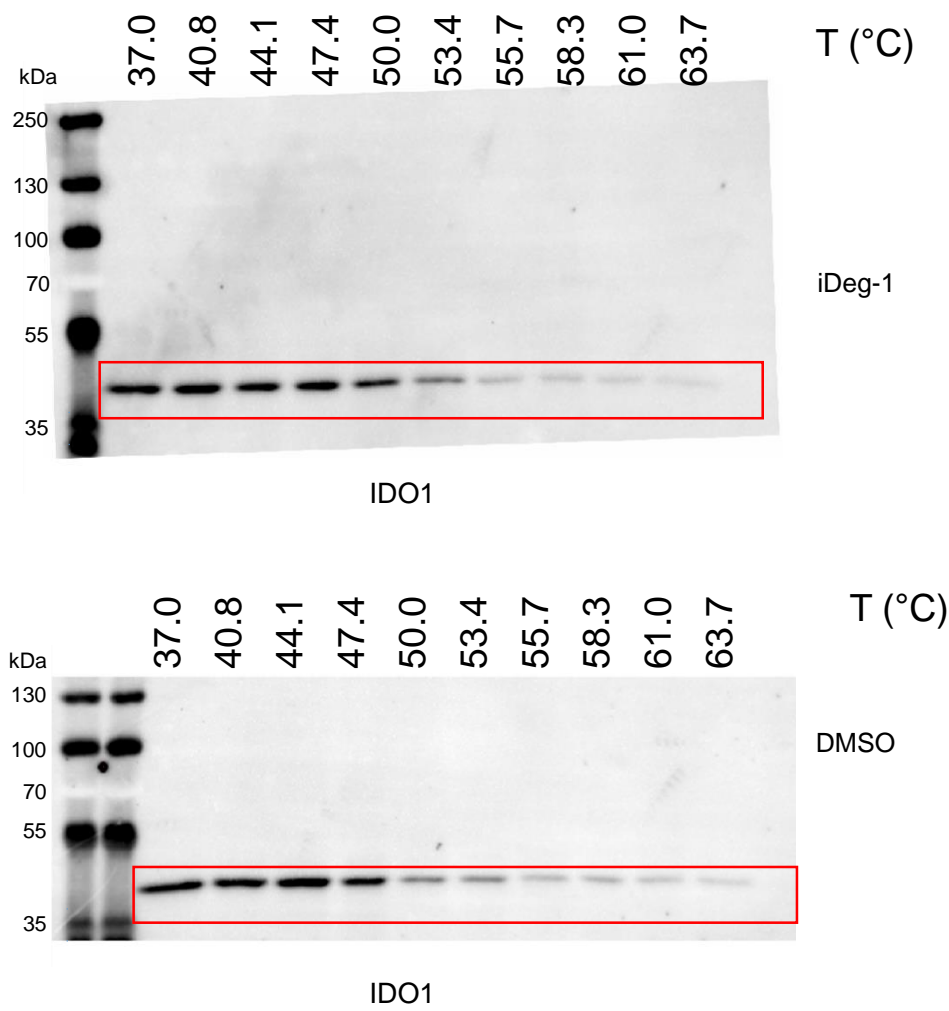

Figure 2d

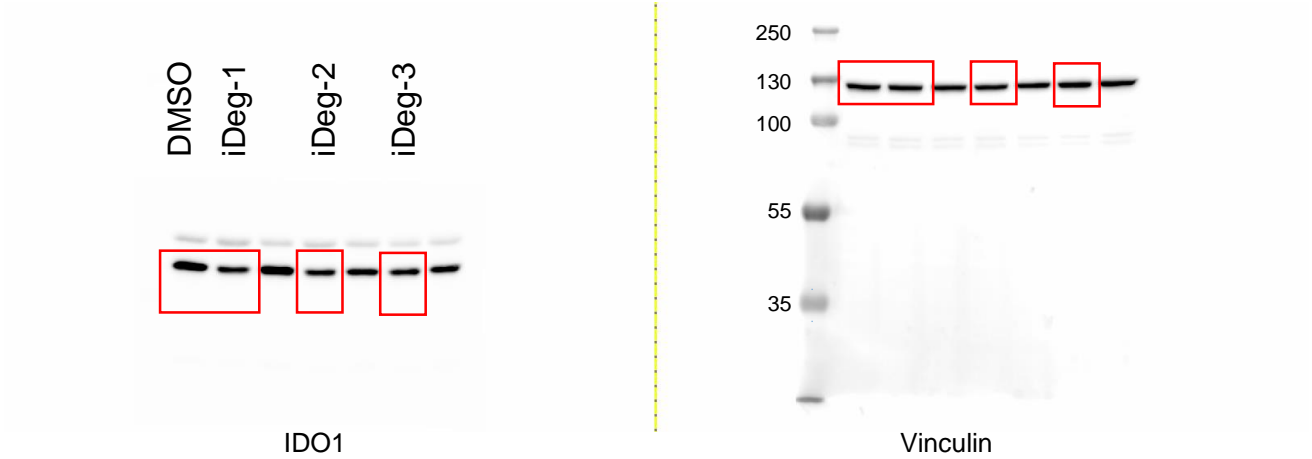

Supplement: Supplementary file 8 — Unprocessed western blots. [file 41557_2025_2021_MOESM8_ESM.pdf]

Figure 4i

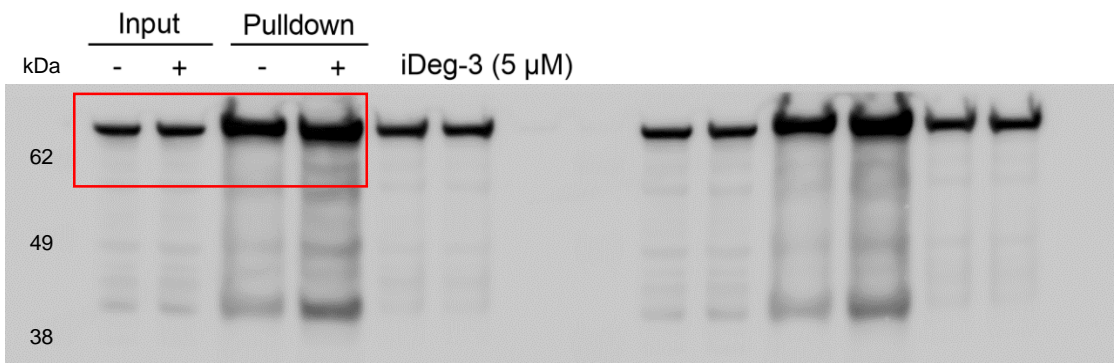

Turbo-IDO1

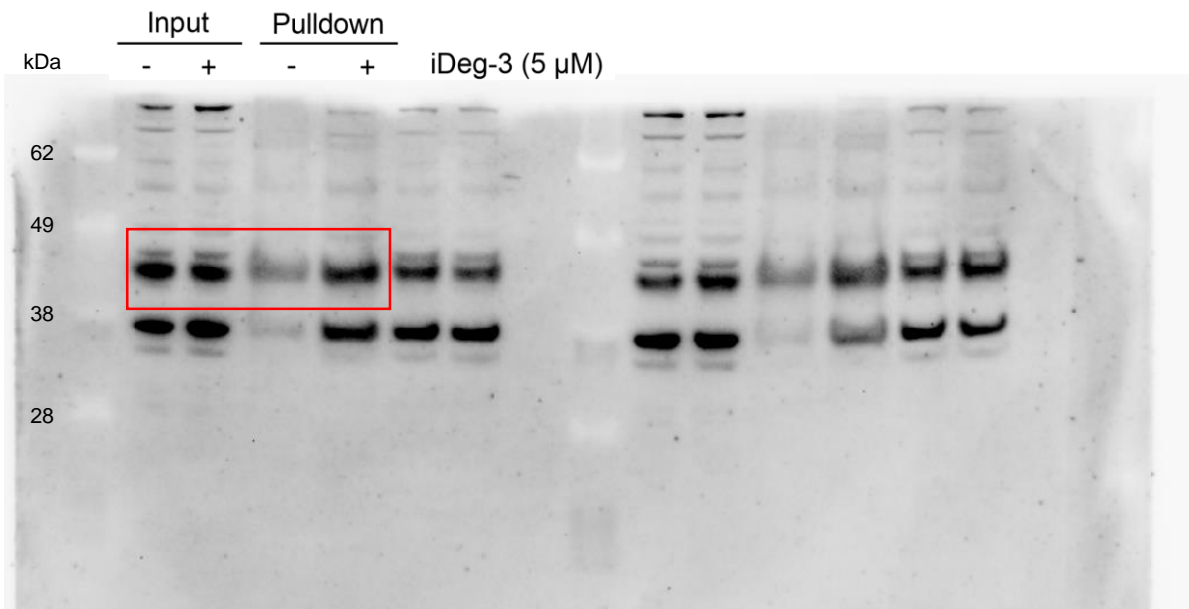

KLHDC3

Figure 4k

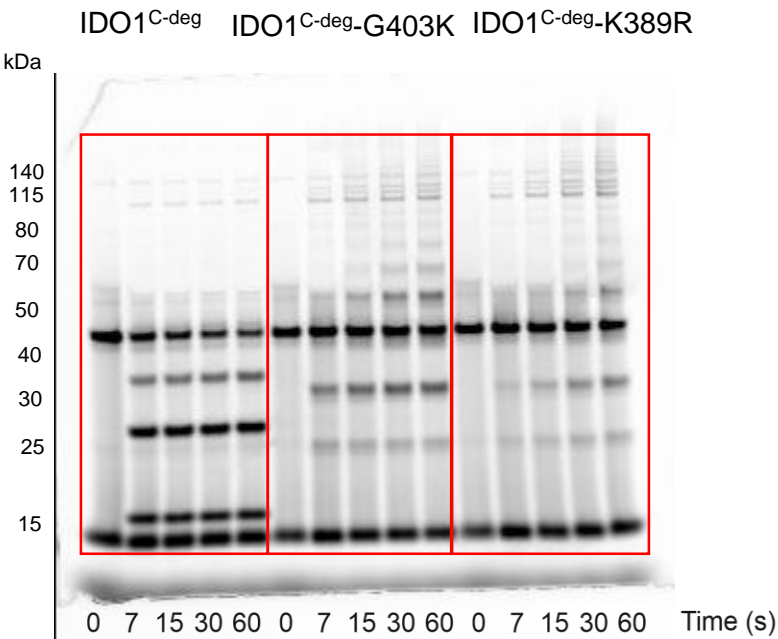

Supplement: Supplementary file 10 — Unprocessed western blots. [file 41557_2025_2021_MOESM10_ESM.pdf]

**Figure 5b**

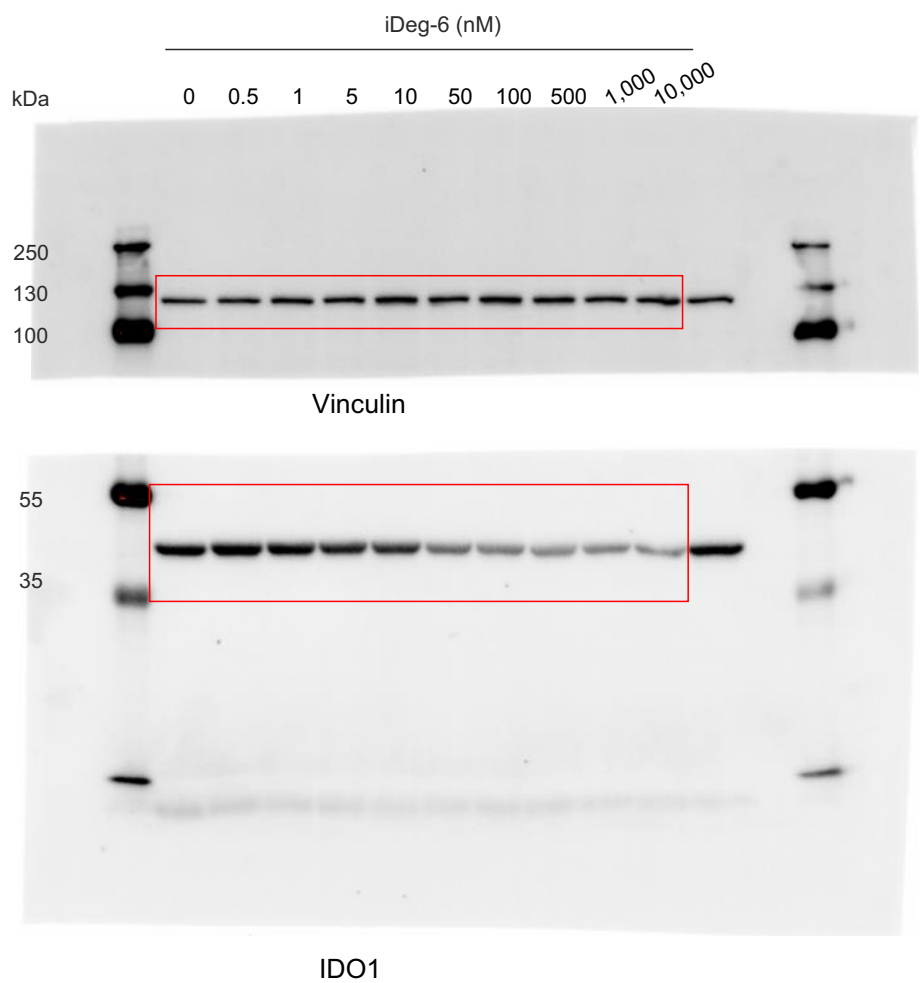

**Figure 5d**

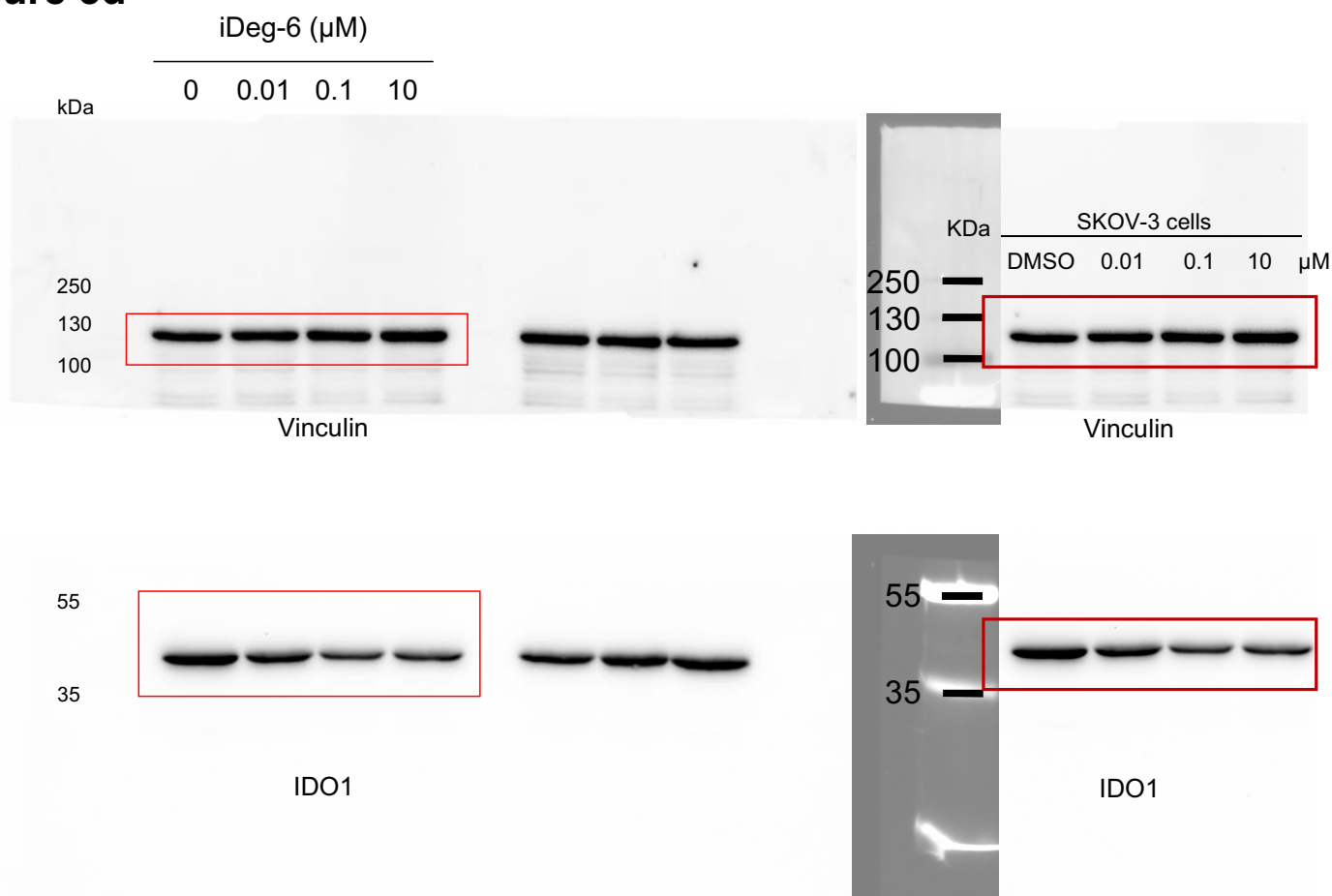

Figure 5e

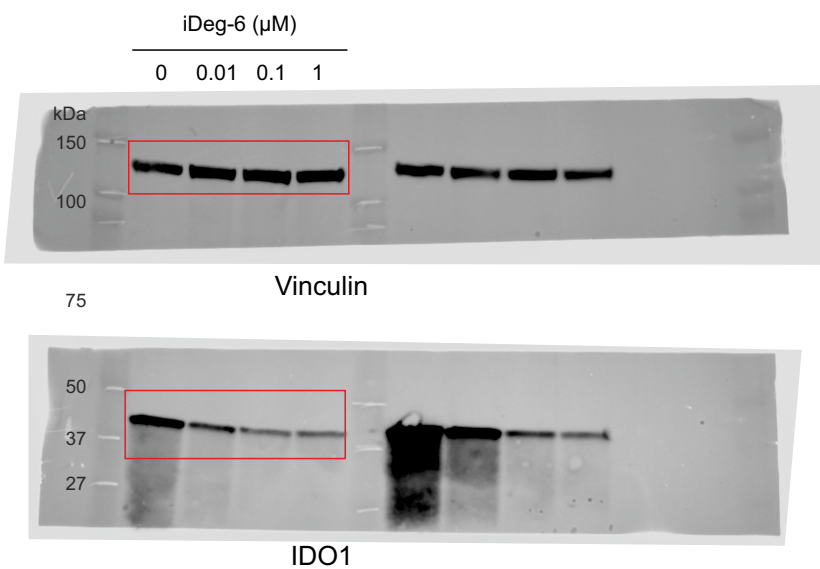

Figure 5f

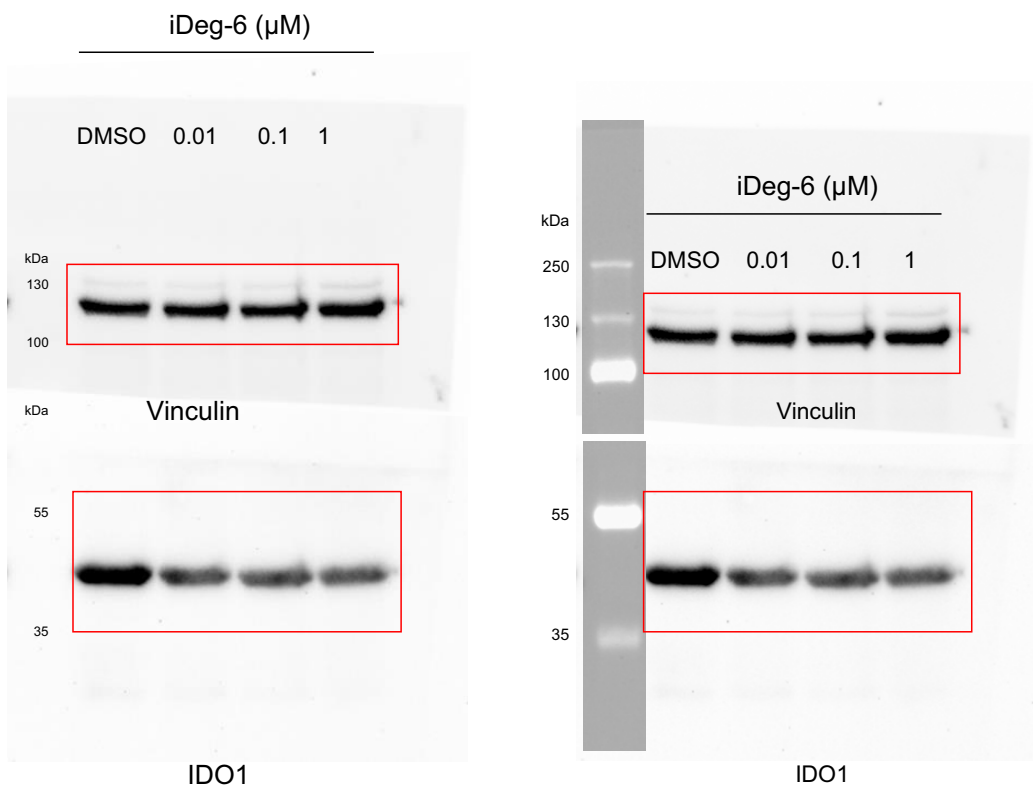

Figure 5g

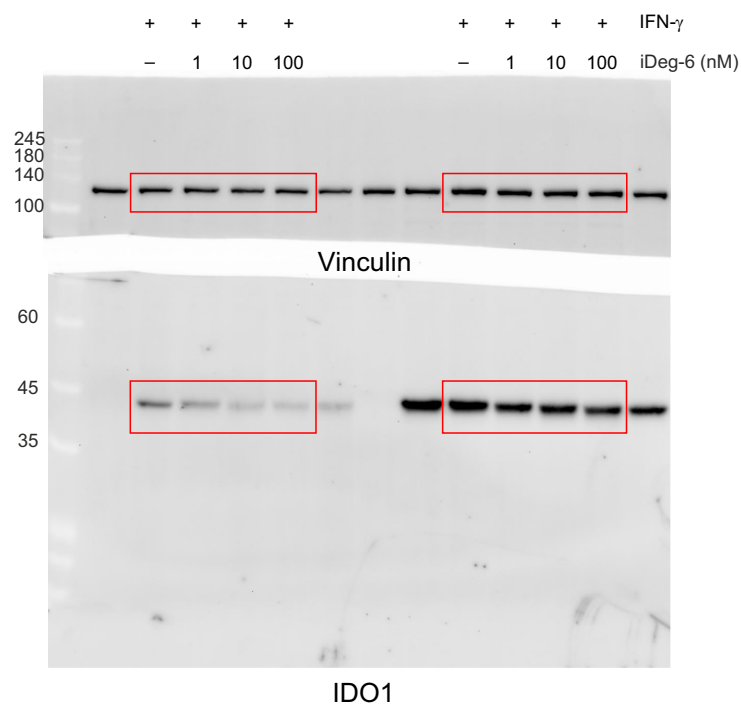

Supplement: Supplementary file 12 — Unprocessed western blots. [file 41557_2025_2021_MOESM12_ESM.pdf]

Figure 6b

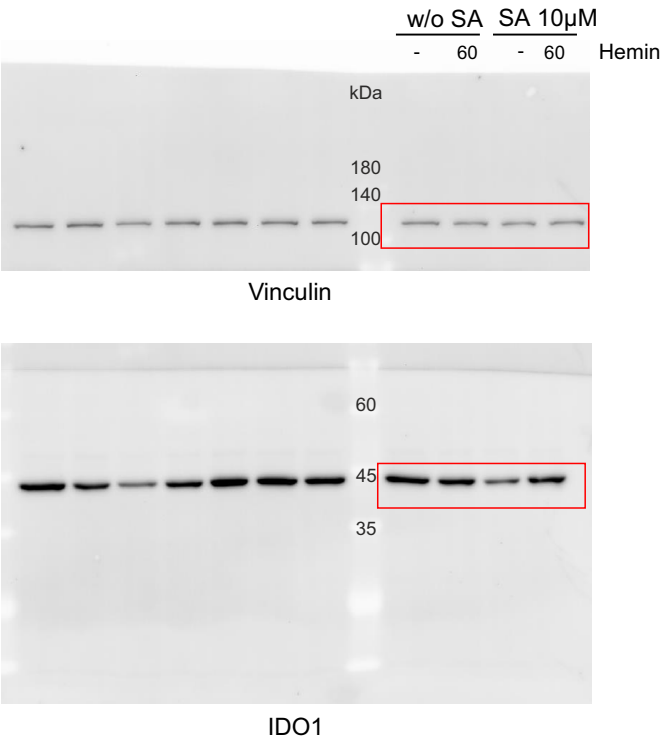

Figure 6d

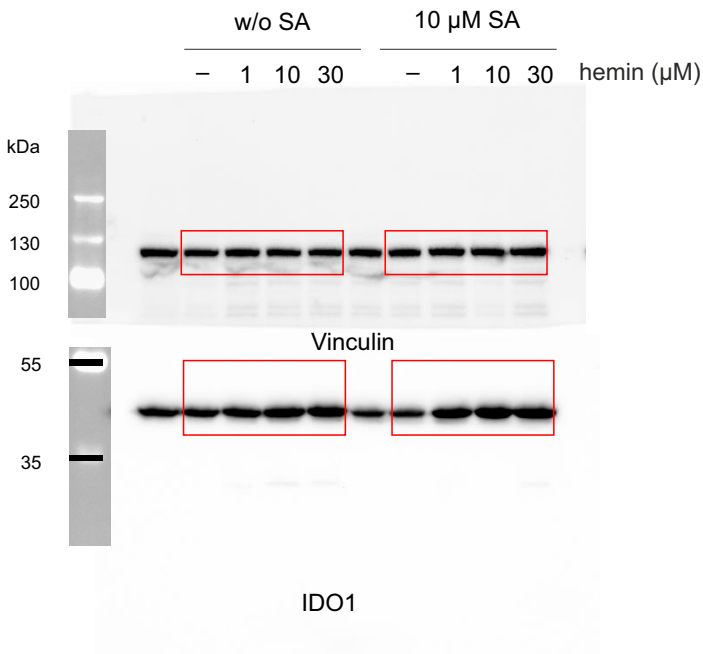

Figure 6g

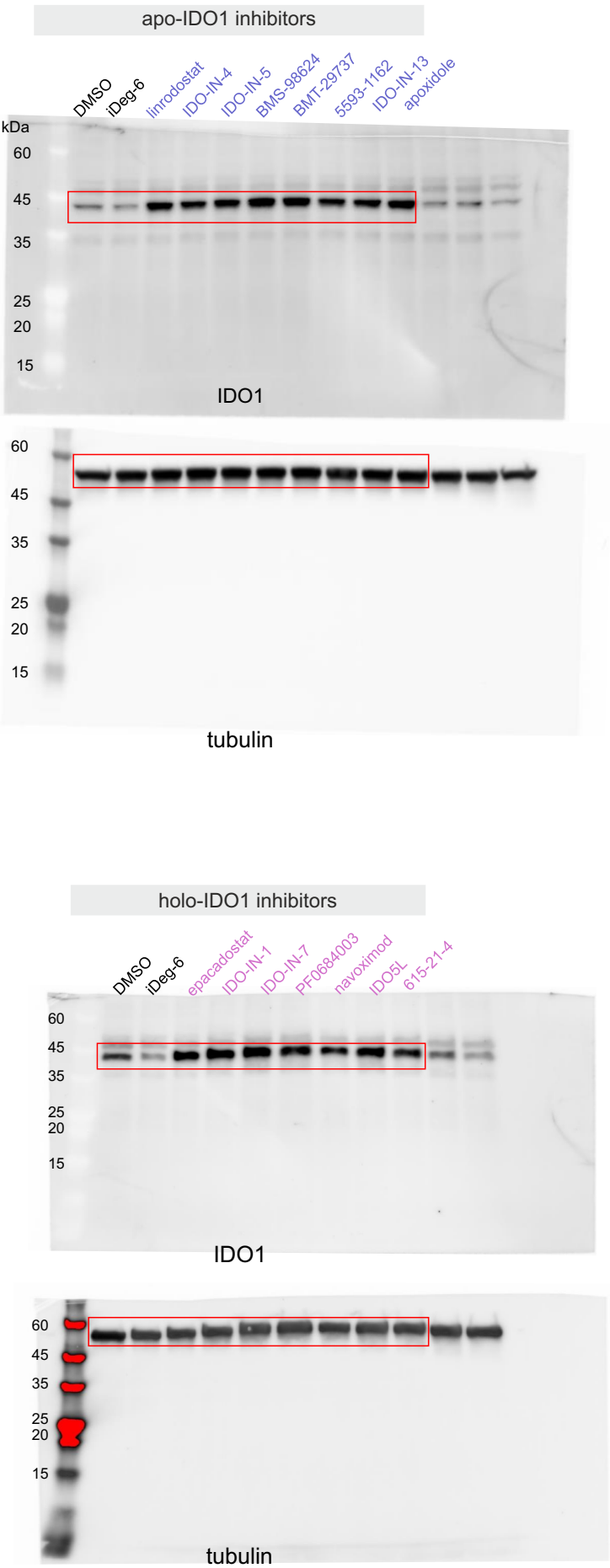

Supplement: Supplementary file 14 — Unprocessed western blots. [file 41557_2025_2021_MOESM14_ESM.pdf]

Extended Data Figure 1i

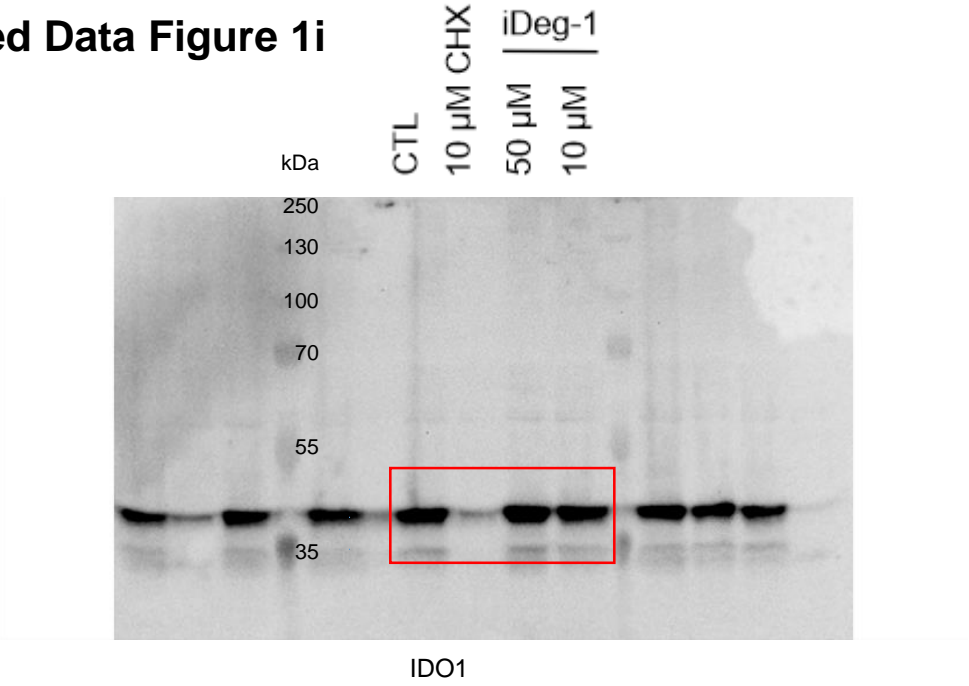

Extended Data Figure 1k

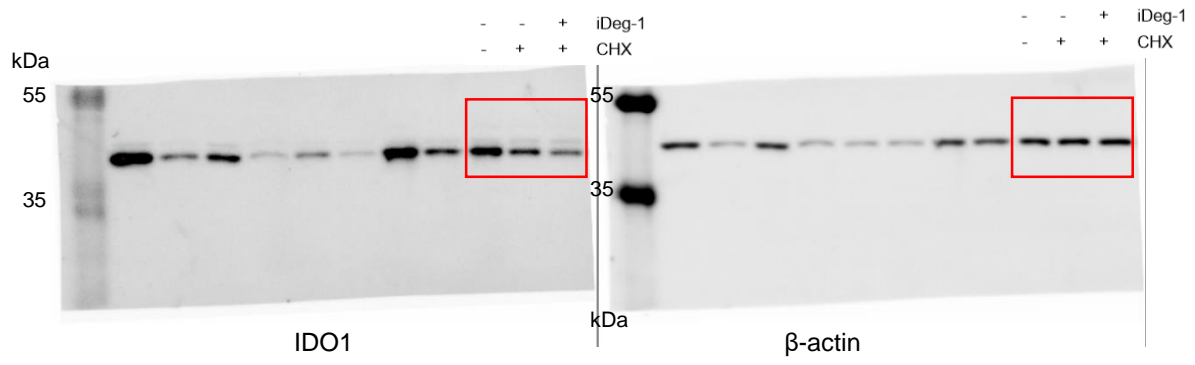

Supplement: Supplementary file 16 — Unprocessed western blots. [file 41557_2025_2021_MOESM16_ESM.pdf]

# Extended Data Figure 2a

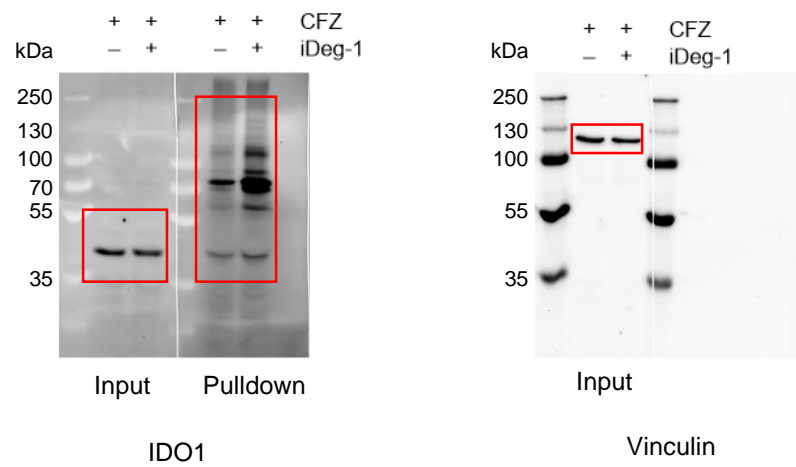

# Extended Data Figure 2d

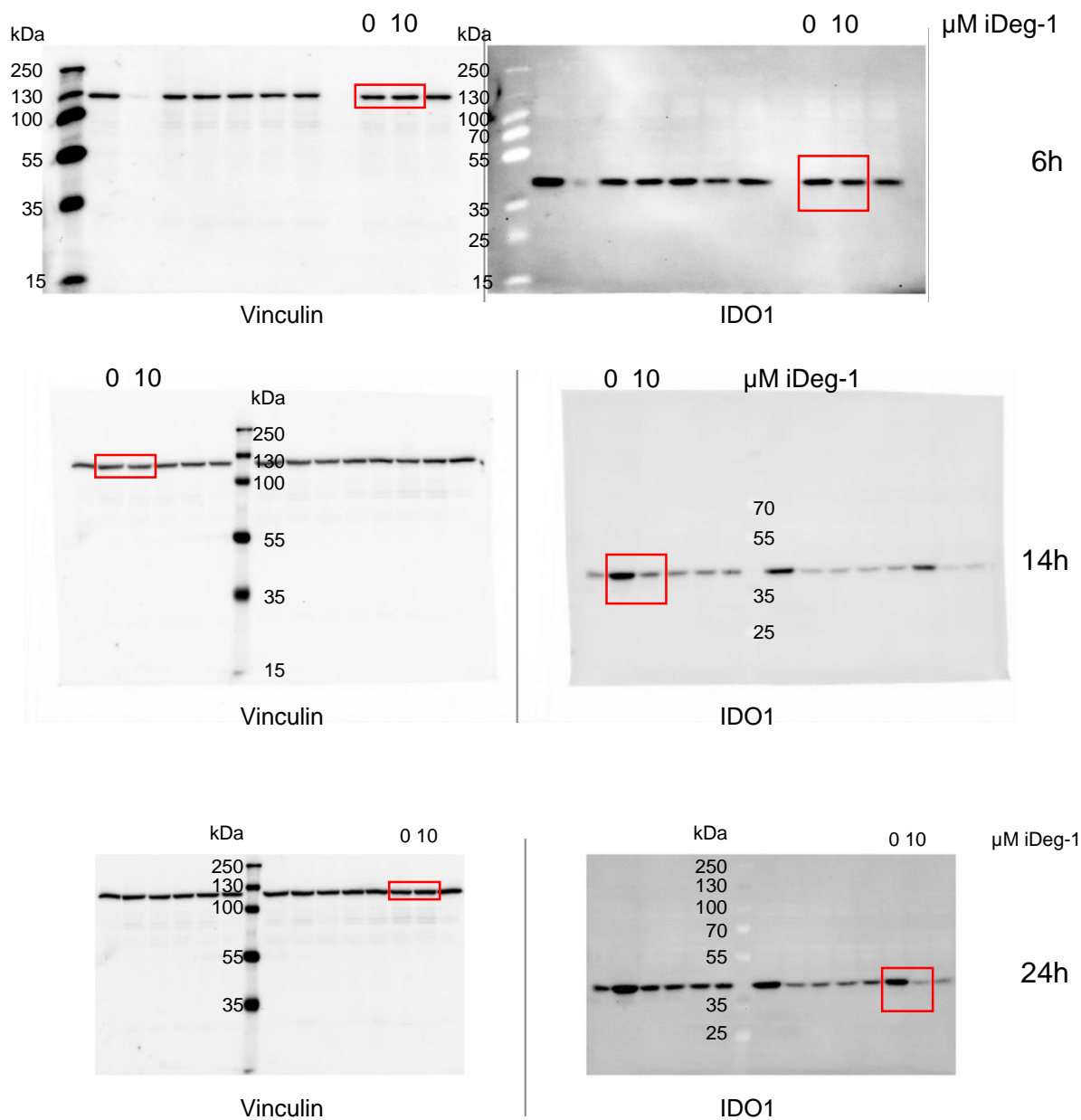

Supplement: Supplementary file 18 — Unprocessed western blots. [file 41557_2025_2021_MOESM18_ESM.pdf]

Extended Data Figure 3a

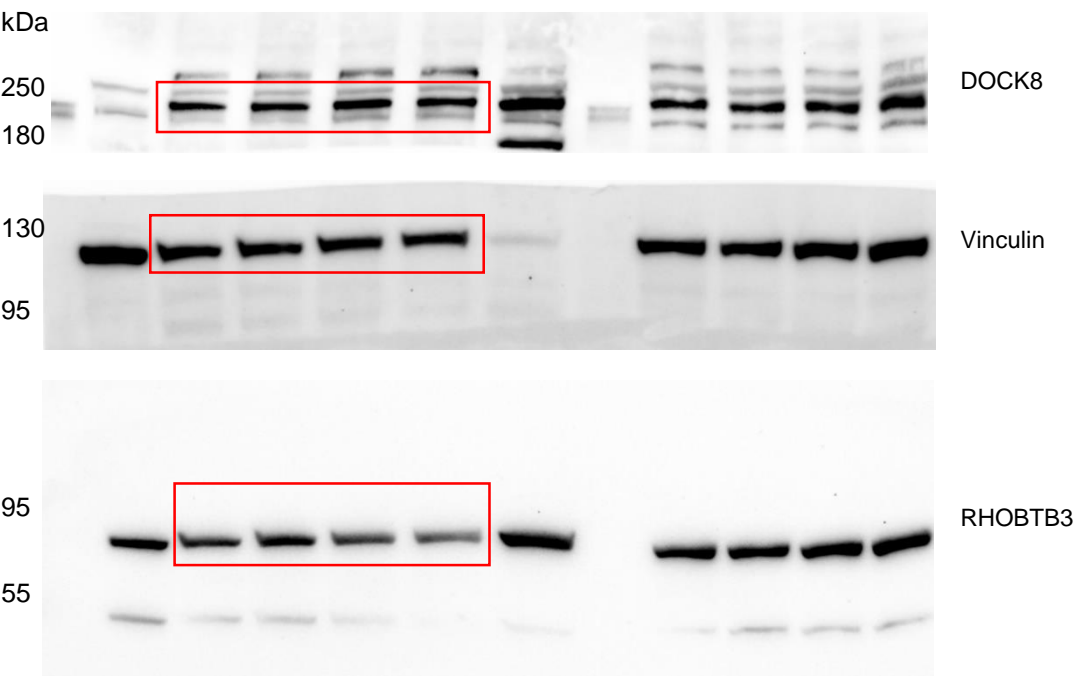

Supplement: Supplementary file 20 — Unprocessed western blots. [file 41557_2025_2021_MOESM20_ESM.pdf]

### Extended Data Figure 4a

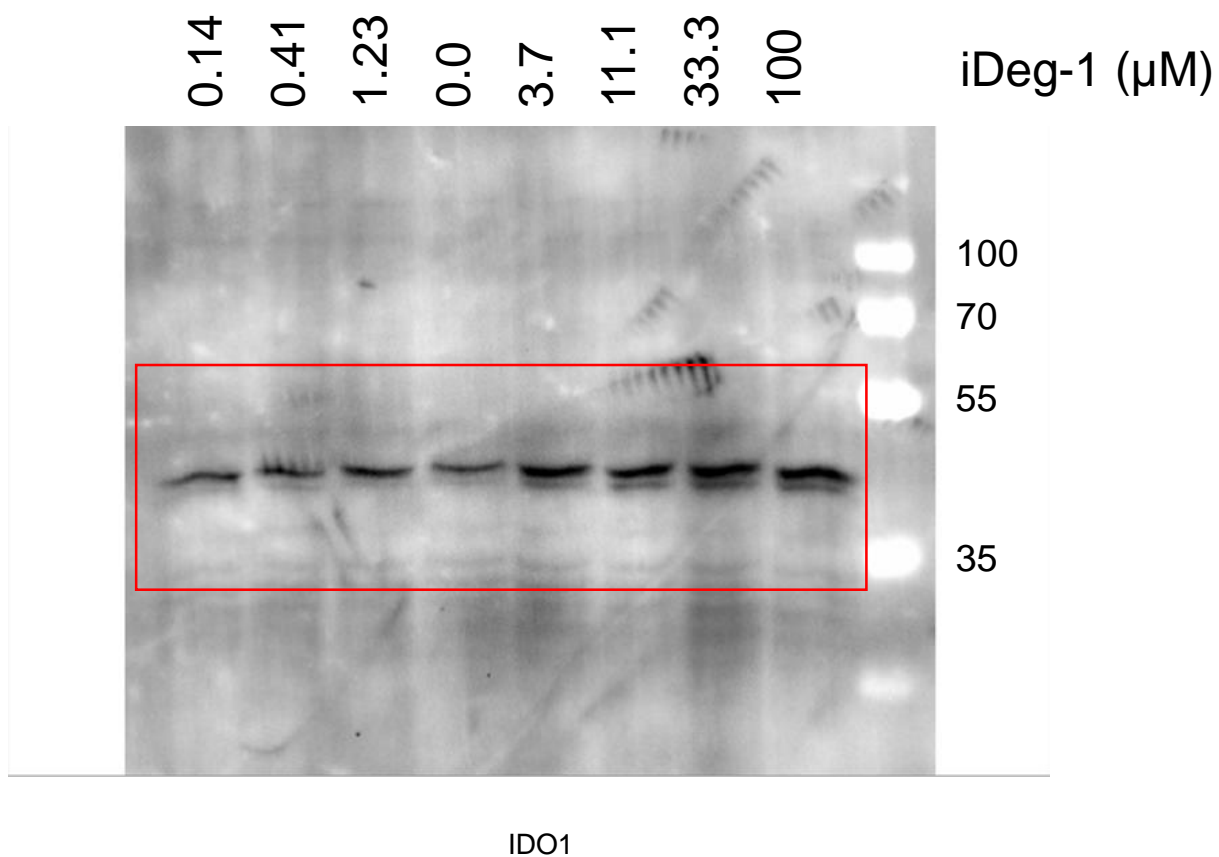

Supplement: Supplementary file 22 — Unprocessed western blots. [file 41557_2025_2021_MOESM22_ESM.pdf]

Extended Data Fig. 7b

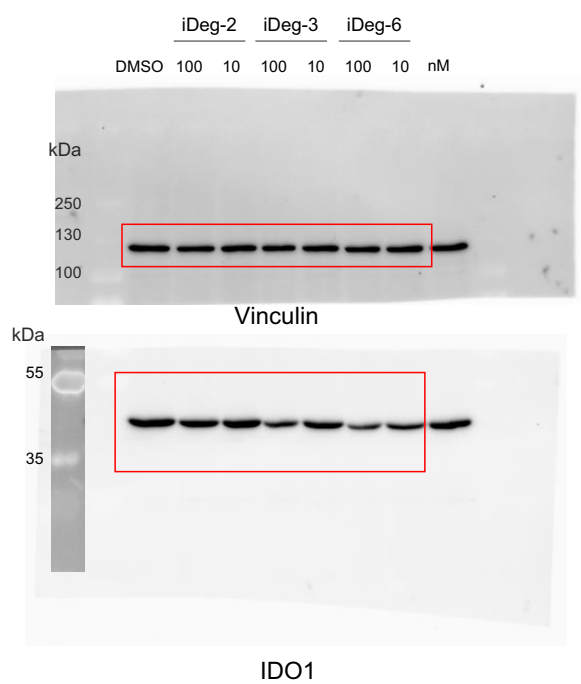

Extended Data Fig. 7j

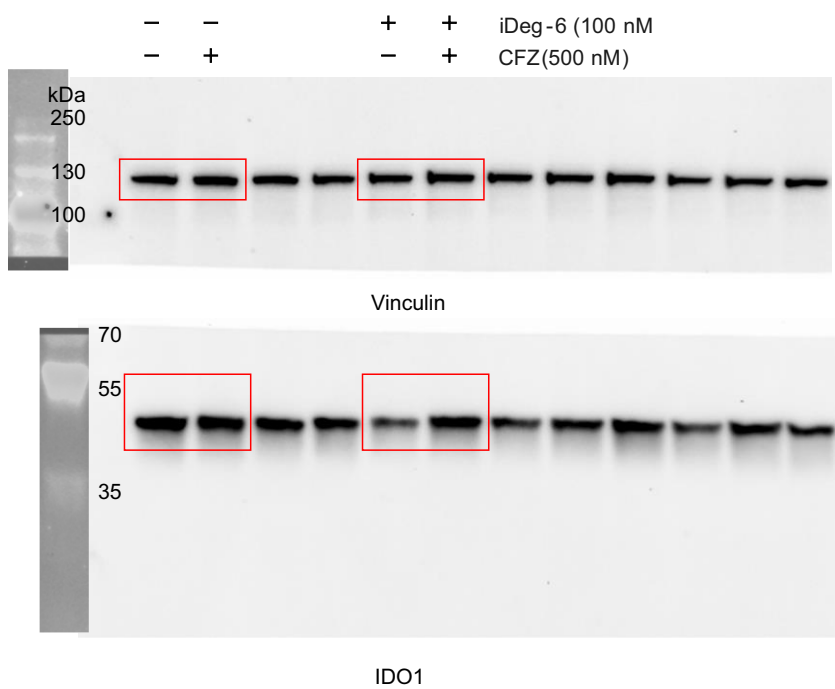

Supplement: Supplementary file 25 — Unprocessed western blots. [file 41557_2025_2021_MOESM25_ESM.pdf]

Extended Data Fig. 9a

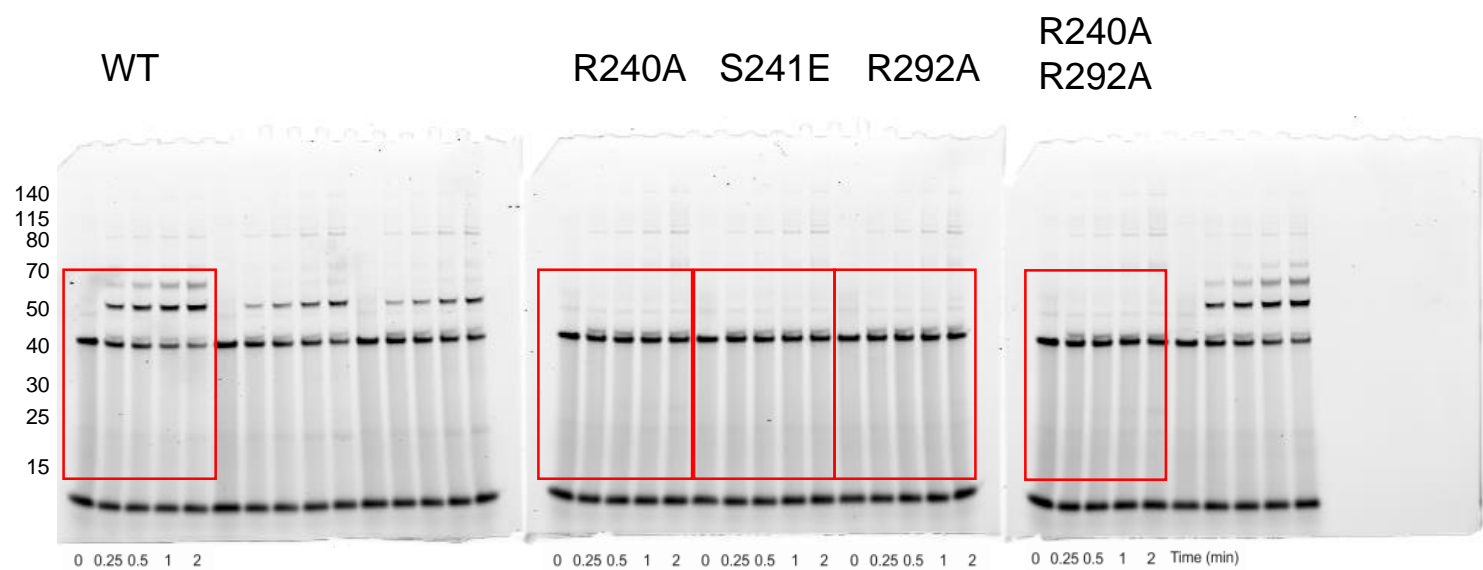

Extended Data Fig. 9c

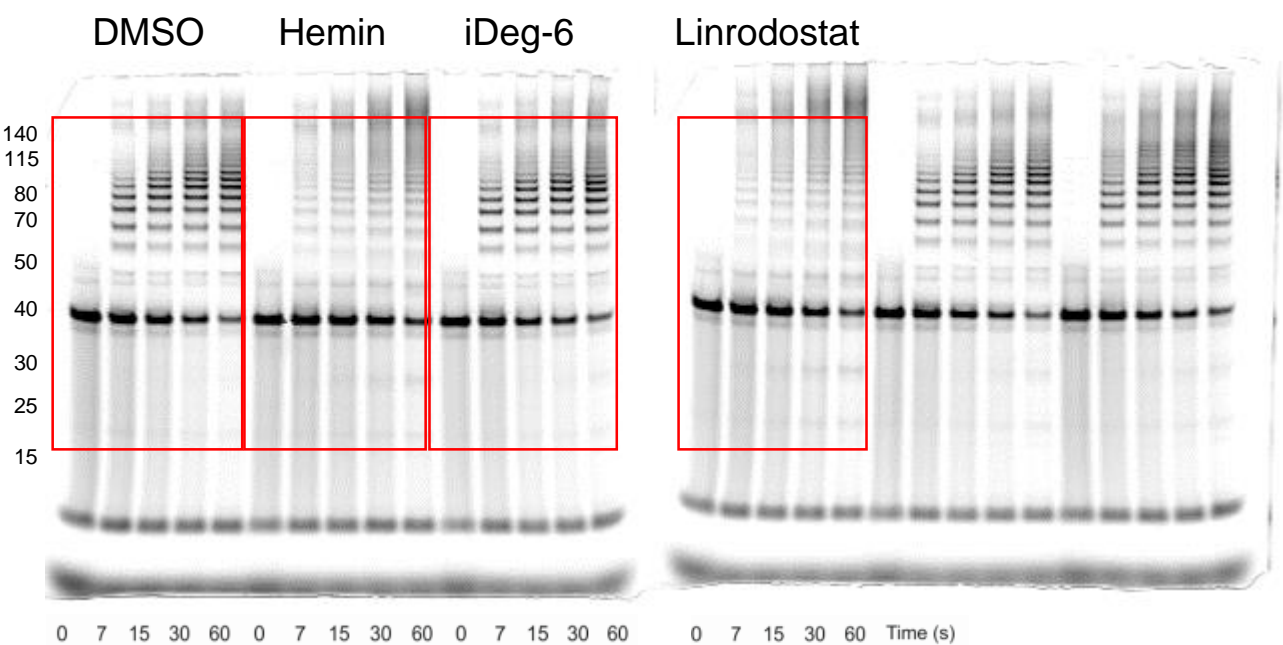

Extended Data Fig. 9d

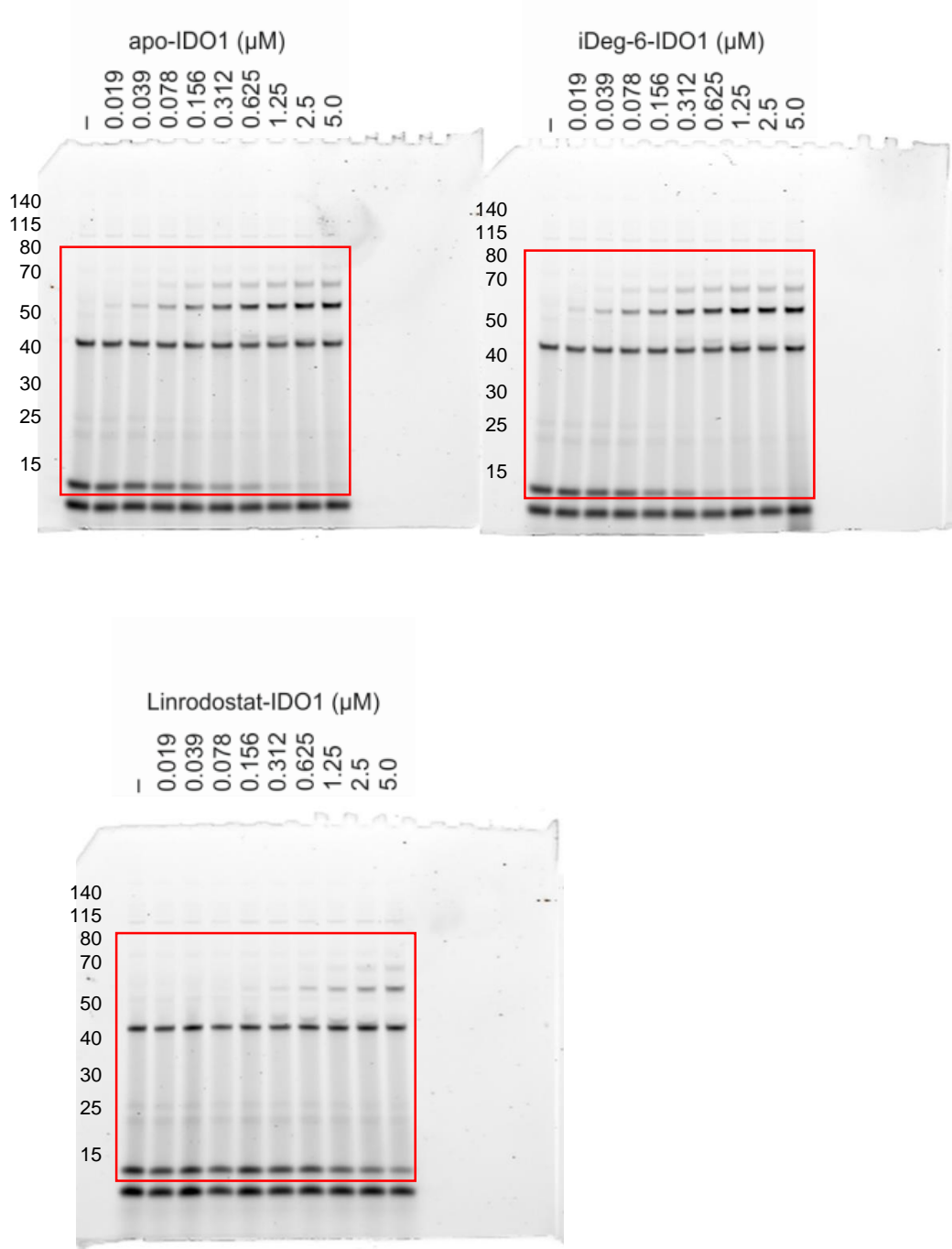

Extended Data Fig. 9e

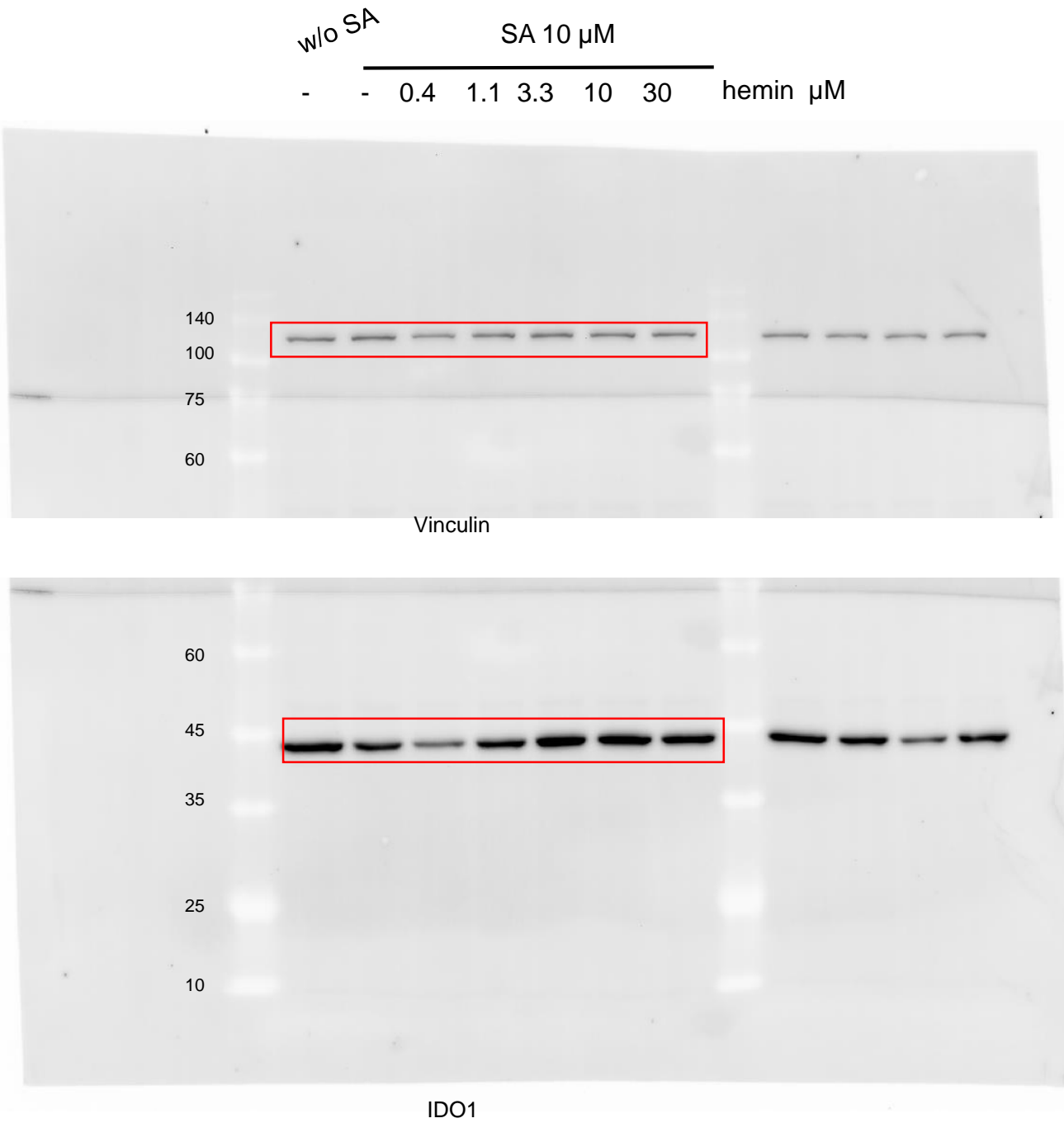

Supplement: Supplementary file 29 — Unprocessed western blots. [file 41557_2025_2021_MOESM29_ESM.pdf]
