# Supplementary material for: Monovalent pseudo-natural products supercharge degradation of IDO1 by its native E3 KLHDC3
Source: Nat Chem. 2026 Jan 7;18(3):585–96. doi: 10.1038/s41557-025-02021-5 (PMC12962974; doi:10.1038/s41557-025-02021-5)

Extended Data Fig. 8b

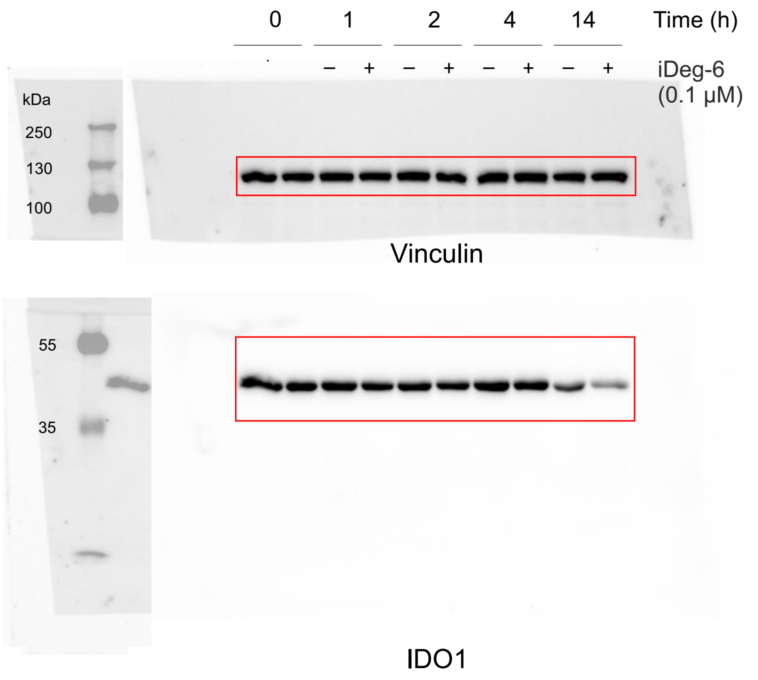

Extended Data Fig. 8d

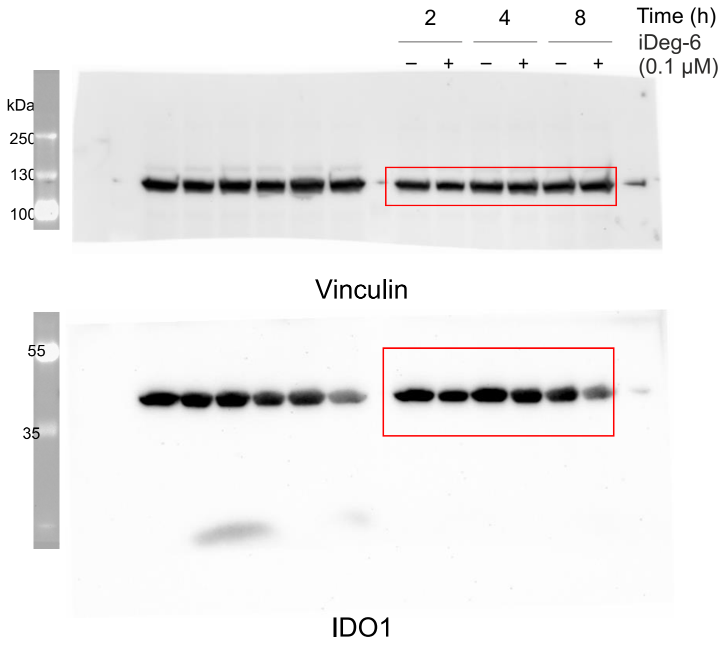

Extended Data Fig. 8f

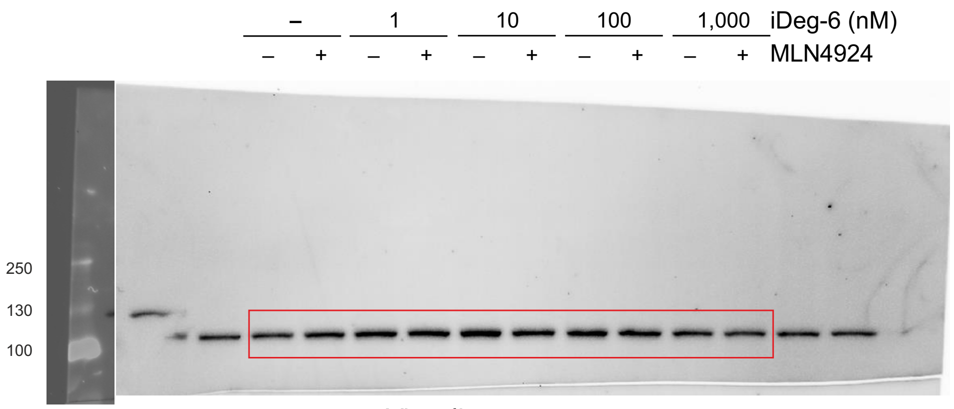

Vinculin

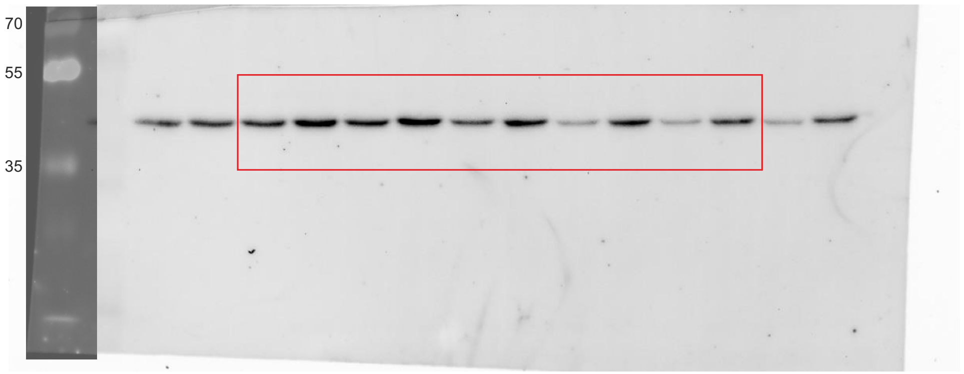

IDO1

Extended Data Fig. 8g

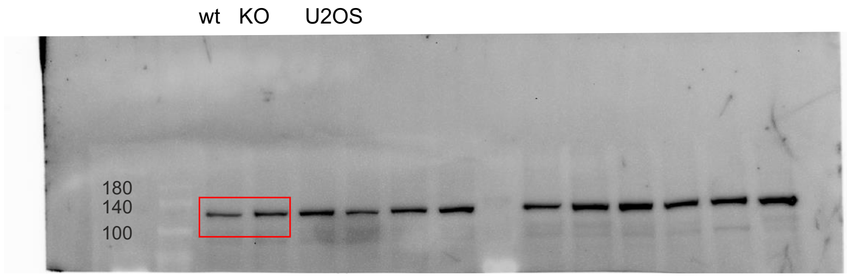

Vinculin

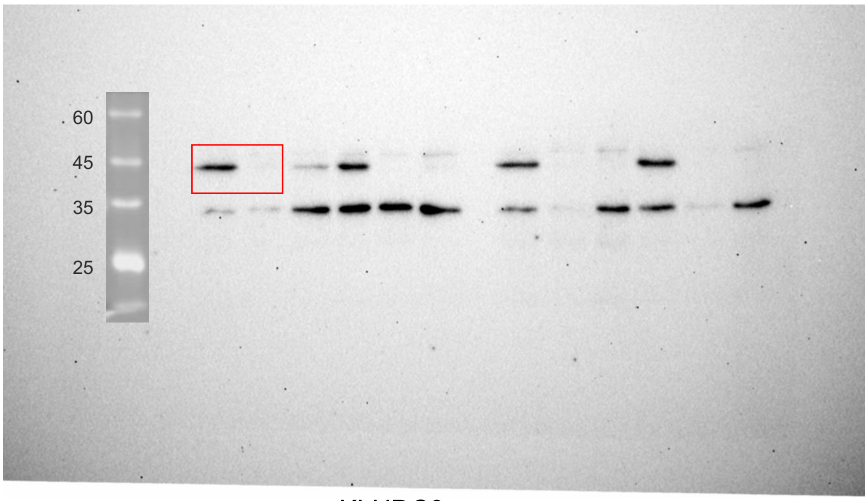

KLHDC3

Extended Data Fig. 8h

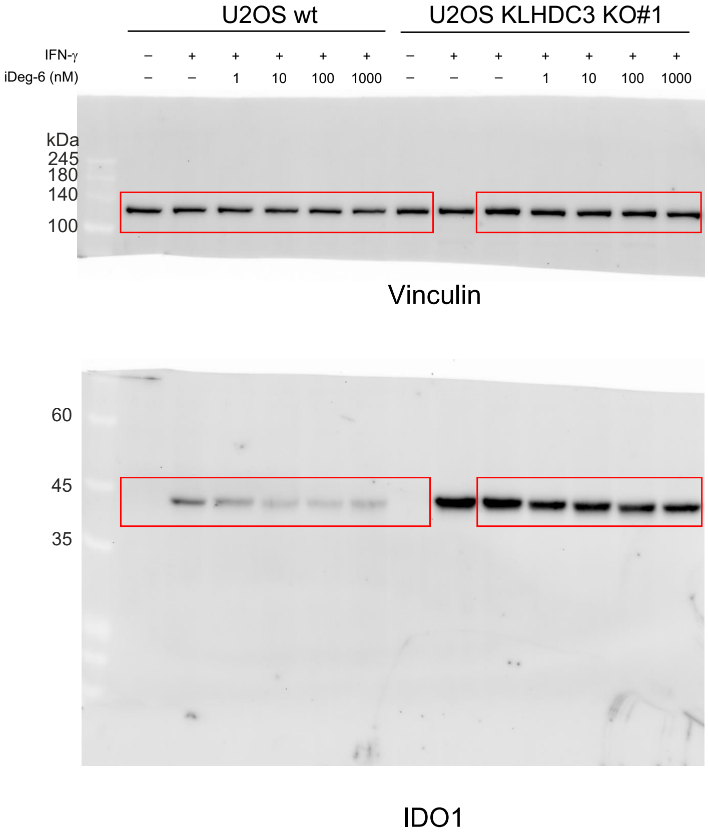

Supplement: Supplementary file 27 — Unprocessed western blots. [file 41557_2025_2021_MOESM27_ESM.pdf]
